# Supplementary material for: Pregnancy physiology pattern prediction study (4P study): protocol of an observational cohort study collecting vital sign information to inform the development of an accurate centile-based obstetric early warning score
Source: BMJ Open. 2017 Sep 1;7(9):e016034. doi: 10.1136/bmjopen-2017-016034 (PMC5589023; doi:10.1136/bmjopen-2017-016034)
Supplement: Supplementary file 1 [file bmjopen-2017-016034supp001.pdf]

# APPENDICES

## Appendix 1: Sites in study

John Radcliffe Hospital, Oxford University Hospitals NHS Foundation Trust, UK

Royal Victoria Infirmary, The Newcastle Upon Tyne Hospitals NHS Foundation Trust, UK

St Thomas' Hospital, Guy's and St Thomas' NHS Foundation Trust, UK

## Appendix 2: Inclusion/Exclusion criteria

Inclusion Criteria

a) ≥16 years of age ( parental consent to be obtained for participants <18 years old)

b) BMI < 35 kg/m2

c) Ultrasound dating scan < 14 weeks

d) Naturally conceived pregnancy

e) Singleton pregnancy

f) Pregnant with an intent to deliver in hospital

g) Able to understand written or spoken English or has their own interpreter willing to translate

h) Willing and able to use the home monitoring equipment

i) Within category 1 of the American Society of Anaesthesiologists' classification of physical status at enrolment ("A normal healthy patient without any clinically important co-morbidity and without a clinically significant past/present medical history"). Women with the following conditions would be included in the 4P study, under the above definition:
   - Obesity
   - Smoking
   - Diabetes
   - Renal disease without hypertension
   - High BP at recruitment (defined as SBP > 140mmHg and DBP > 90mmHg, not previously diagnosed with hypertension)
   - Gestational hypertension or pre-eclampsia diagnosed in a previous pregnancy (even if treated, as long as essential hypertension was not developed)

Exclusion Criteria

Any known medical condition expected by the recruiting clinician to alter maternal vital signs, such as:

- Hypertension (defined as SBP > 140mmHg and DBP > 90mmHg), ever requiring treatment
- Congenital cardiac disease
- Ischaemic cardiac disease

- Bronchiectasis
- Fibrotic lung disease
- Malignant disease

## Appendix 3: Intrapartum case report form (electronic)

Intrapartum data is transcribed from patient notes through electronic case report forms. Screenshots of these intrapartum forms are presented in the following four figures.

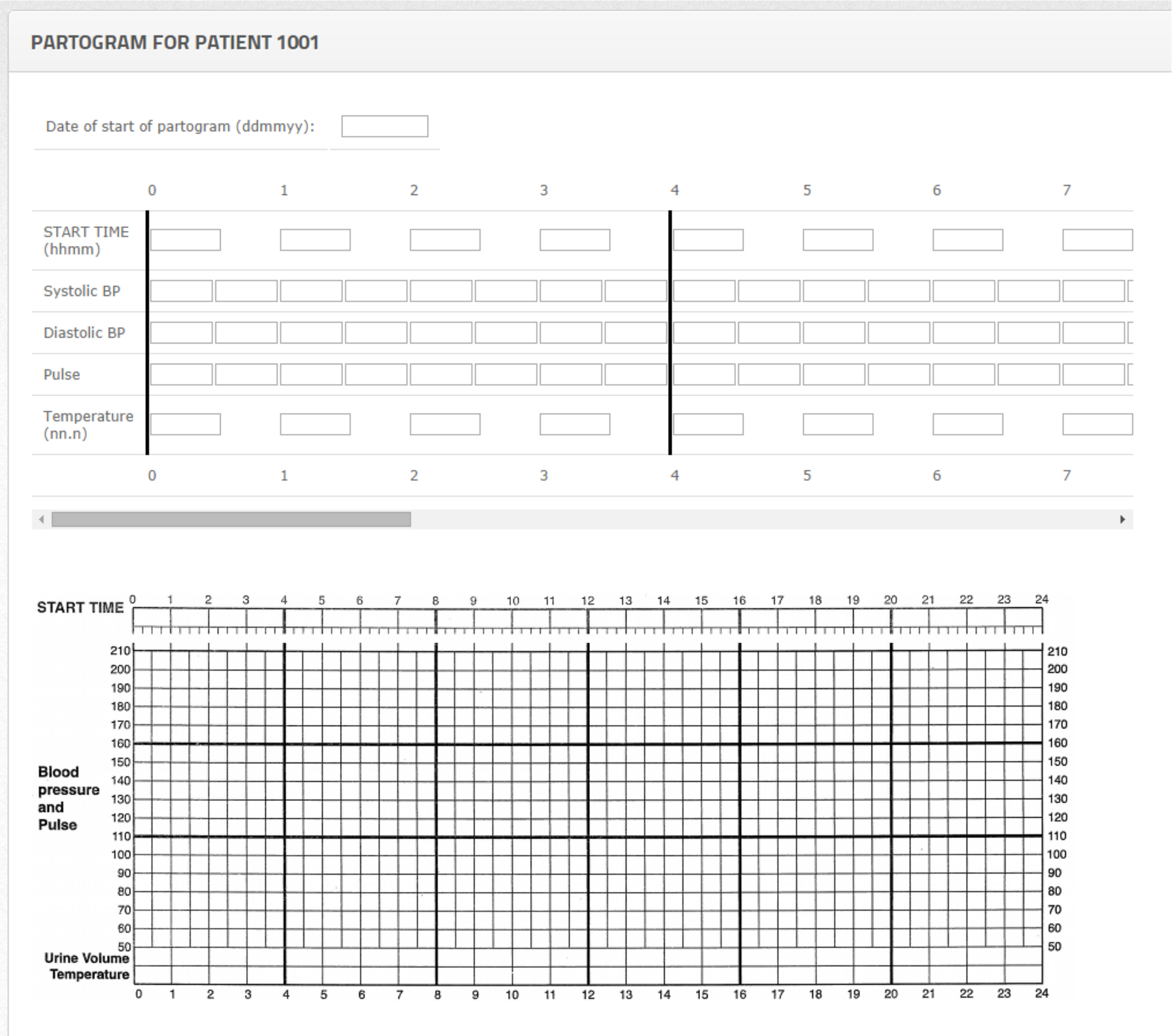
**PARTOGRAM FOR PATIENT 1001**

Date of start of partogram (ddmmyy):

| | 0 | 1 | 2 | 3 | 4 | 5 | 6 | 7 |
|---|---|---|---|---|---|---|---|---|
| START TIME (hhmm) | | | | | | | | |
| Systolic BP | | | | | | | | |
| Diastolic BP | | | | | | | | |
| Pulse | | | | | | | | |
| Temperature (nn.n) | | | | | | | | |
| | 0 | 1 | 2 | 3 | 4 | 5 | 6 | 7 |



*Partogram chart*

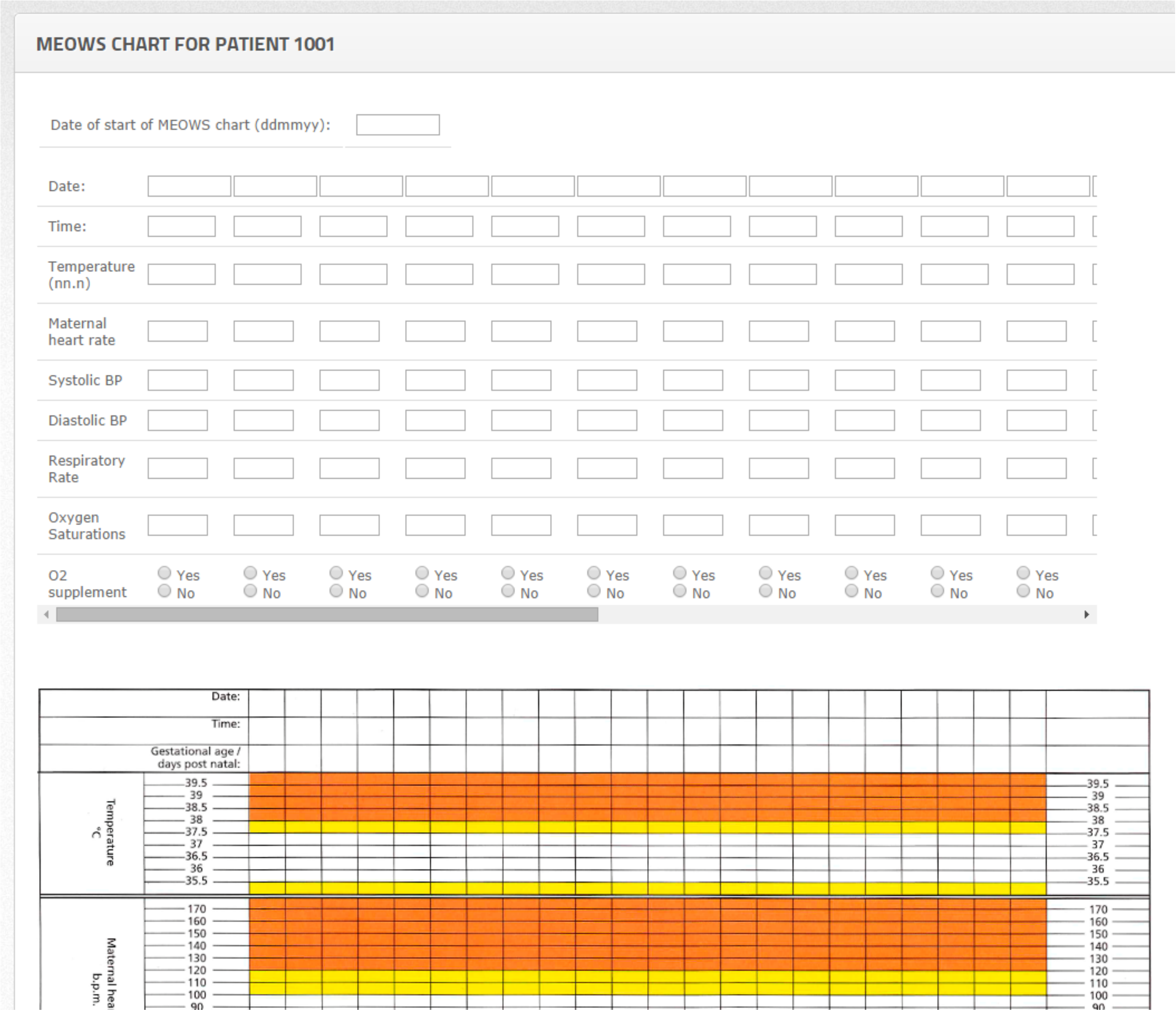


*Maternal early warning score chart (referred to as a MEOWS chart in OUHs).*

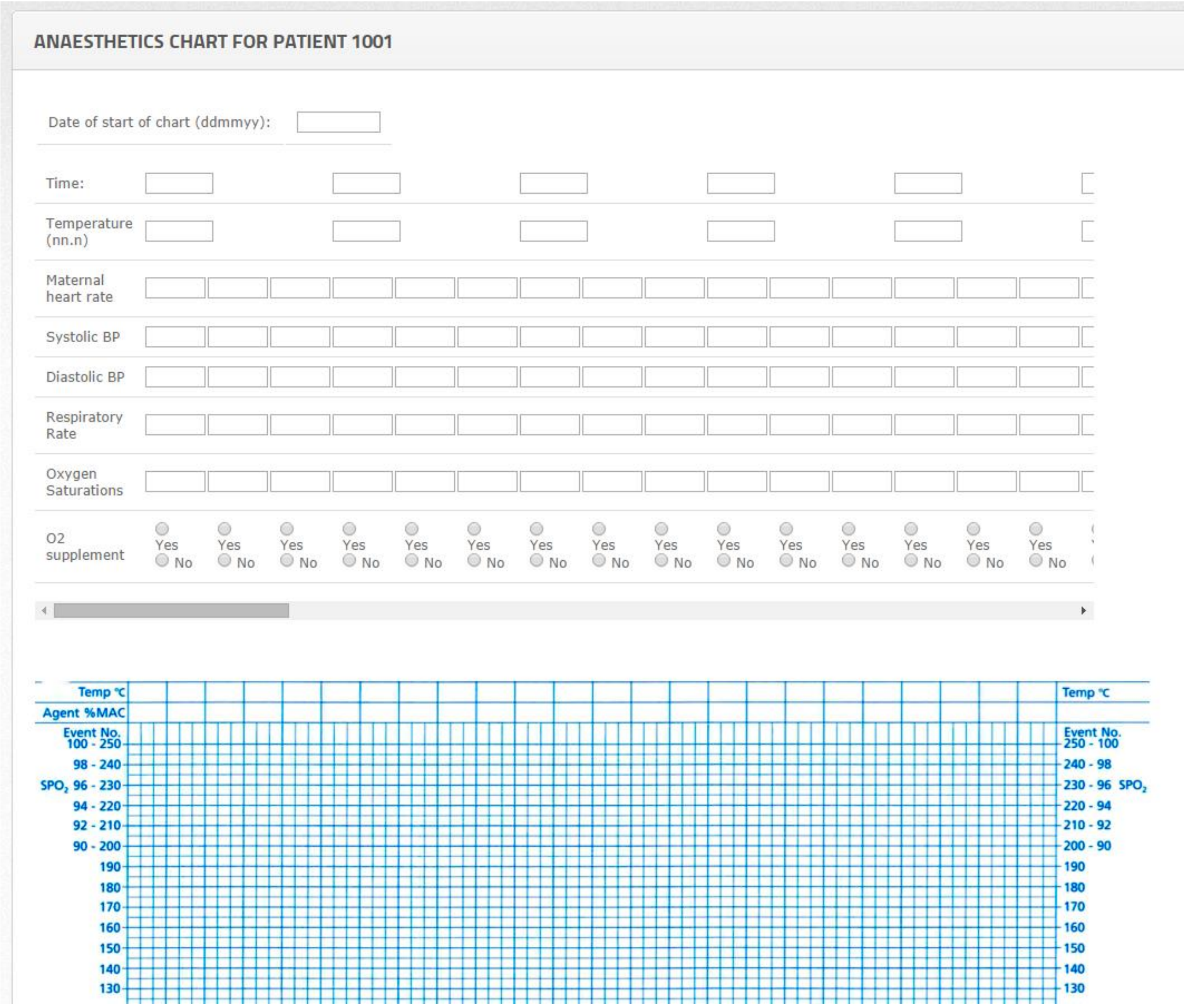


*Anaesthetics chart*

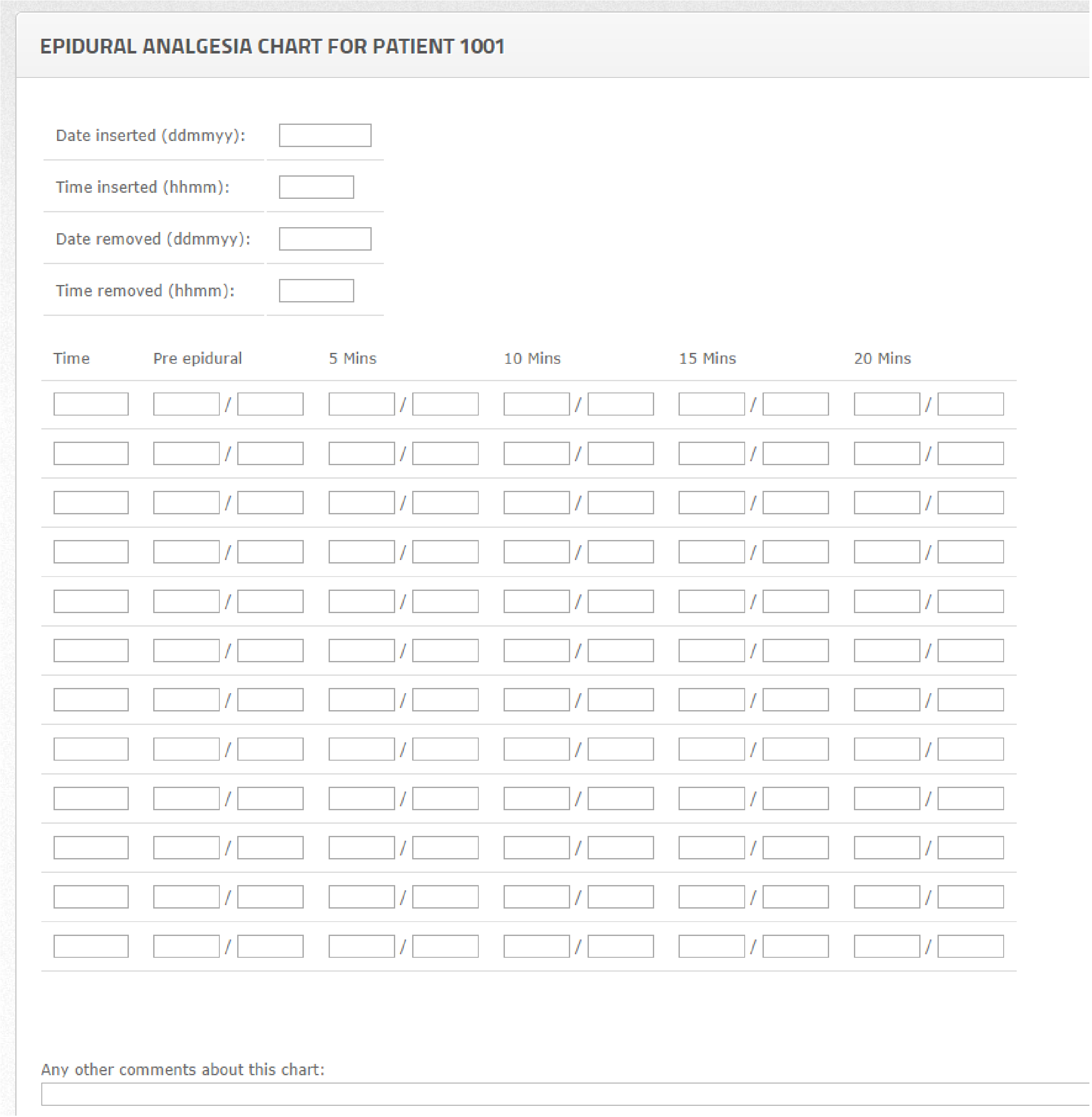

**EPIDURAL ANALGESIA CHART FOR PATIENT 1001**

Date inserted (ddmmyy):

Time inserted (hhmm):

Date removed (ddmmyy):

Time removed (hhmm):

| Time | Pre epidural | 5 Mins | 10 Mins | 15 Mins | 20 Mins |
|---|---|---|---|---|---|
| | / | / | / | / | / |
| | / | / | / | / | / |
| | / | / | / | / | / |
| | / | / | / | / | / |
| | / | / | / | / | / |
| | / | / | / | / | / |
| | / | / | / | / | / |
| | / | / | / | / | / |
| | / | / | / | / | / |
| | / | / | / | / | / |
| | / | / | / | / | / |
| | / | / | / | / | / |

Any other comments about this chart:

*Epidural chart*

## Appendix 4: Case Report Forms

Supporting information are extracted from the participant's notes at each appointment. For example, the form used to collect demographic information, medical and obstetric history, current health status and pregnancy-related health and current medications at the initial baseline assessment, is shown below.

### (1) BASELINE INFORMATION

| | |
|---|---|
| Participant ID: | ___ ___ ___ ___ |
| Date: | ___ ___ - ___ ___ - ___ ___ ___ ___ (D D M M Y Y Y Y) |

**SECTION 1: DEMOGRAPHIC INFORMATION**

| | | | |
|---|---|---|---|
| **1.1** Date of Birth | ___ ___ - ___ ___ - ___ ___ ___ ___ | **1.7** Haemoglobin Level | ___ ___ . ___ g/dl |
| **1.2** Height | ___ ___ ___ . ___ cm | **1.8** OR Haematocrit | ___ ___ . ___ % |
| **1.3** Weight | ___ ___ ___ . ___ kg | **1.9** Date of blood test: | ___ ___ - ___ ___ - ___ ___ ___ ___ |
| **1.4** In the last three months, has she smoked? | ☐ Yes ☐ No | **1.10** Proteinuria (by dipstick) | ☐ 0/trace ☐ + ☐ ++ |
| **1.5** If yes, how many cigarettes/cigars per day? | ___ ___ | ☐ +++ ☐ ++++ | ☐ No urine test available |
| **1.6** Has she lived in the same household as someone who smokes? | ☐ Yes ☐ No | **1.11** Date of urine test: | ___ ___ - ___ ___ - ___ ___ ___ ___ |

| **1.12** Ethnicity | White | Asian or Asian British | Black or Black British |
|---|---|---|---|
| | ☐ A : White - British | ☐ H : Indian | ☐ M : Black - Caribbean |
| | ☐ B : White - Irish | ☐ J : Pakistani | ☐ N : Black - African |
| | ☐ C : Any other White background | ☐ K : Bangladeshi | ☐ P : Any other Black background |
| | Mixed | ☐ L : Any other Asian background | Other ethnic group |
| | ☐ D : Mixed - White and Black Caribbean | | ☐ R : Chinese |
| | ☐ E : Mixed - White and Black African | | ☐ S : Any other Ethnic Group |
| | ☐ F : Mixed - White and Asian | | ☐ Z : Does not want to give Ethnic Group |
| | ☐ G : Any other mixed background | | |

**SECTION 2: MEDICAL HISTORY**

*Has she ever been diagnosed with or treated for any of the following medical conditions?*

| | | | |
|---|---|---|---|
| **2.1** Diabetes | ☐ Yes ☐ No | **2.9** Hepatic disease | ☐ Yes ☐ No |
| **2.2** Thyroid disease | ☐ Yes ☐ No | **2.10** Gastrointestinal disease | ☐ Yes ☐ No |
| **2.3** Other endocrinological condition | ☐ Yes ☐ No | **2.11** Autoimmune conditions | ☐ Yes ☐ No |
| **2.4** Any type of malignancy/cancer (including leukaemia or lymphoma) | ☐ Yes ☐ No | **2.12** Haematological conditions | ☐ Yes ☐ No |
| **2.5** Cardiac disease | ☐ Yes ☐ No | **2.13** Venous thromboembolism (VTE) | ☐ Yes ☐ No |
| **2.6** Hypertension without treatment | ☐ Yes ☐ No | **2.14** Any congenital abnormality or genetic disease | ☐ Yes ☐ No |
| **2.7** A chronic respiratory disease (including chronic asthma) | ☐ Yes ☐ No | If yes, specify: ______________________________ | |
| **2.8** Proteinuria, kidney disease or chronic renal disease | ☐ Yes ☐ No | **2.15** Any other clinically relevant condition | ☐ Yes ☐ No |
| | | If yes, specify: ______________________________ | |

(Continued on next page)

| SECTION 3: OBSTETRIC AND GYNAECOLOGICAL HISTORY | |
|---|---|
| **3.1** Date of first day of last menstrual period (LMP) | ___ ___ - ___ ___ - ___ ___ ___ ___ (D D M M Y Y Y Y) |
| **3.2** Number of previous pregnancies, excluding this pregnancy | ___ ___ |
| **3.3** Number of previous births | ___ ___ |

| SECTION 4: CURRENT PREGNANCY-RELATED HEALTH | |
|---|---|
| **4.1** CRL (Crown Rump Length) measurement: | ___ ___ ___ . ___ mm |
| **4.2** Date of CRL measurement: | ___ ___ - ___ ___ - ___ ___ ___ ___ (D D M M Y Y Y Y) |

*During this pregnancy, has she been diagnosed with or treated for any of the following conditions?*

| | | | |
|---|---|---|---|
| **4.3** Severe vomiting requiring hospitalisation | ☐ Yes ☐ No | **4.5** Anaemia | ☐ Yes ☐ No |
| **4.4** Hypertension | ☐ Yes ☐ No | **4.6** Infection/febrile illness | ☐ Yes ☐ No |

**SECTION 5: CURRENT MEDICATIONS**

*Is she routinely taking any of the following medication?*

| | | | |
|---|---|---|---|
| **5.1** Aspirin | ☐ Yes ☐ No | **5.5** Any other antibiotics/antivirals | ☐ Yes ☐ No |
| **5.2** Non-steroidal anti-inflammatories | ☐ Yes ☐ No | If yes, specify: ______________________ | |
| **5.3** Insulin | ☐ Yes ☐ No | **5.6** Any other treatment | ☐ Yes ☐ No |
| **5.4** Antihypertensives | ☐ Yes ☐ No | If yes, specify: ______________________ | |
| Which antihypertensives? | ☐ ACE inhibitors<br>☐ Beta-blockers<br>☐ Calcium channel blockers | | |

## Appendix 5: Definitions

Definitions of variables collected in the 4P study, mostly taken from the National Institute for Health and Care Excellence (NICE) guidelines and quality standards.

| Baseline information | |
|---|---|
| Ethnicity | Categories from the 2011 UK Census<br>A White - British<br>B White - Irish<br>C Any other White background<br>D Mixed - White and Black Caribbean<br>E Mixed - White and Black African<br>F Mixed - White and Asian<br>G Any other mixed background<br>H Indian<br>J Pakistani<br>K Bangladeshi<br>L Any other Asian background<br>M Black - Caribbean<br>N Black - African<br>P Any other Black background<br>R Chinese<br>S Any other Ethnic Group<br>Z Does not want to give Ethnic Group |
| Weight | Measured in kilograms (kg) to 1 decimal place. |
| Height | Measured in centimetres (cm) to 1 decimal place. |
| Crown Rump Length | Ultrasound measurement of crown rump length in millimetres (mm) to 1 decimal place. |
| Gestational age | Calculated from Crown Rump Length to the nearest day, using the algorithm developed by the Interbio study [1]. |
| Smoker | Has smoked in the three months before recruitment. |
| Proteinuria | Presence of proteinuria in the urine, measured in milligrams/decilitre (mg/dl) to 1 decimal place. |
| **Medical Conditions** | |
| Diabetes | Glucose intolerance. Any type of diabetes is recorded. |
| Thyroid disease | A medical condition impairing the function of the thyroid. For example: hypo- or hyper-thyroidism, arathyroidism (PTH). |

| | |
|---|---|
| Other endocrinological condition | Diseases related to the endocrine glands of the body. For example: Addison's disease, adrenal gland disorders, hypophysitis |
| Malignancy/cancer | A malignant tumour, where cells proliferate out of control and inhibit the normal function of an organ system. Any type of cancer recorded, including leukaemia or lymphoma. |
| Cardiac disease | Disease of the heart or blood vessels. For example: arrhythmias, murmurs, valve diseases, atherosclerosis, atrial fibrillation, sarcoma, pericarditis, or cardiomyopathy. |
| Respiratory disease | Diseases of the airways and other parts of the lung. For example: asthma, chronic obstructive pulmonary disease (COPD), lung cancer, cystic fibrosis, sleep apnoea or occupational lung diseases. |
| Pyelonephritis or kidney disease | Disease of the kidneys. For example: chronic kidney disease, polycystic kidney disease, or kidney failure. |
| Hepatic disease | Disease of the liver. For example: hepatitis, haemochromatosis, or alcohol-related liver disease. |
| Gastrointestinal disease | Diseases involving the gastrointestinal tract (oesophagus, stomach, small intestine, large intestine and rectum), and the accessory organs of digestion (liver, gallbladder, and pancreas). For example: Crohn's disease, coeliac disease, ulcerative colitis or any severe malabsorption condition (requiring special diet) |
| Autoimmune conditions | The body's immune system attacks and destroys healthy body tissue by mistake. There are more than 80 types of autoimmune disorders. For example: Addison's disease, multiple sclerosis, rheumatoid arthritis. |
| Haematological conditions | Disorder of the blood. For example: anaemia, bleeding disorders such as haemophilia, blood clots, and blood cancers such as leukaemia, lymphoma, and myeloma. |
| Venous thromboembolism | A blood clot in a vein. |
| Infection | The invasion and multiplication of microorganisms such as bacteria, viruses, and parasites that are not normally present within the body. |
| Febrile illness | A nonspecific term for any illness of sudden onset accompanied by fever. |
| **Pregnancy complications** | |
| Gestational diabetes | Any degree of glucose intolerance with onset or first recognition during pregnancy. |
| Gestational hypertension | Blood pressure is ≥ 140/90mmHg after 20 weeks gestation in a previously normotensive pregnancy [2] |
| Preeclampsia | Gestational hypertension with the presence of proteinuria (excessive protein substance, chiefly albumin, in the urine) |

| | |
|---|---|
| Severe preeclampsia | Blood pressure is ≥ 160/110mmHg on two occasions, between 4 and 168 hours apart, or if the first measurement was immediately followed by treatment with an antihypertensive, either of these scenarios being associated with the presence of proteinuria. |
| Eclampsia | The occurrence of convulsions and/or coma unrelated to her cerebral conditions in a woman with signs and symptoms of pre-eclampsia. Seizures are of grand mal type and may first appear before labour, during labour, or up to 48 hours postpartum. |
| HELLP syndrome | A group of symptoms that occur in pregnant women who have pre-eclampsia or eclampsia and who also show signs of liver damage and abnormalities in blood clotting. It is characterised by: **H**aemolysis, **EL** (elevated) liver enzymes and **LP** (low platelet) count. |
| Primary postpartum haemorrhage | Blood loss from the genital tract >500mls (vaginal birth) or >1000mls (caesarean birth) within 24 hours of the birth of a baby. [3] |
| Secondary postpartum haemorrhage | Excessive or abnormal bleeding from the genital tract between 24 hours and 6 weeks in the postnatal period. [4] |
| Anaemia | Iron deficiency. Anaemia in pregnancy is defined as first trimester haemoglobin (Hb) less than 110 g/l, second/third trimester Hb less than 105 g/l, and postpartum Hb less than 100 g/l, in line with British Committee for Standards in Haematology (BCSH) guidance. [5] |
| **Delivery and labour** | |
| Spontaneous vaginal delivery | Spontaneous initiation of labour and delivery with no intervention. |
| Vaginal assisted | Delivery assisted with forceps or vacuum. Vacuum extraction uses a suction cup that is placed over the baby's head, which allows the physician to pull the child through the birth canal. |
| Caesarean section | An operative delivery of baby through a small insertion to the mother's abdomen. Including elective (planned, performed at or after 39 weeks), and emergency (unplanned) caesarean sections. |
| Assisted breech or breech extraction | The baby is born feet-first or buttocks first and requires assistance. |
| Preterm delivery | Delivery before 37 weeks. |
| Preterm pre-labour rupture of membranes (PPROM) | Spontaneous rupture of the membranes at less than 37 weeks gestation and prior to onset of labour. |
| Foetal distress | Abnormal foetal heart rate (FHR) or biophysical profile (BPP) (as a reason for labour being induced or caesarean section performed) |

References for Appendix 5:


1. Papageorghiou AT, Kennedy SH, Salomon LJ, Ohuma EO, Chiekh Ismail L, Barros FC, Lambert A, Carvalho, M, Jaffer YA, Bertino E, Gravett MG, Altman DG, Purwar M, Noble JA, Pang R, Victoria CG, Bhutta ZA, Villar J. International standards for early fetal size and pregnancy dating based on ultrasound measurement of crown-rump length in the first trimester of pregnancy. *Ultrasound in Obstetrics and Gynecology* Published Online First: 2 November 2014. doi: 10.1002/uog.13448.
2. National Institute for Health and Care Excellence (NICE). Hypertension in pregnancy: diagnosis and management (Clinical guideline 107). 2010. Retrieved 01-08-2016 from: nice.org.uk/guidance/cg107
3. Mousa HA, Alfirevic Z. Treatment for primary postpartum haemorrhage. *Cochrane Database of Systematic Reviews* Published First Online 13 February 2014. doi: 10.1002/14651858.CD003249.pub3
4. Alexander J,Thomas PW, Sanghera J. Treatments for secondary postpartum haemorrhage. *Cochrane Database of Systematic Reviews* Published Online First: 21 January 2002. doi: 10.1002/14651858.CD002867
5. Royal College of Obstetricians and Gynaecologists (RCOG). Blood transfusion in obstetrics (Green-top guideline 47). 2015. Retrieved 01-08-2016 from: https://www.rcog.org.uk/globalassets/documents/guidelines/gtg-47.pdf

## Appendix 6: Usability survey

Oxford University Hospitals NHS Trust

### The 4P Participants' Usability Survey

| *Please answer each question 1-10 by placing a cross (X) on the scale to indicate your experience of using the 4P m-Health system.* | *Strongly disagree 0* | *1* | *2* | *3* | *Strongly agree 4* |
|---|---|---|---|---|---|
| 1. I think I would like to use the 4P system frequently | ☐ | ☐ | ☐ | ☐ | ☐ |
| 2. I find the 4P system unnecessarily complex | ☐ | ☐ | ☐ | ☐ | ☐ |
| 3. I think the 4P system is easy to use | ☐ | ☐ | ☐ | ☐ | ☐ |
| 4. I think that I would need the ongoing support of a technical person or research midwife to be able to use the 4P system | ☐ | ☐ | ☐ | ☐ | ☐ |
| 5. I find that the various functions within the 4P system are well integrated | ☐ | ☐ | ☐ | ☐ | ☐ |
| 6. I think that there is too much inconsistency in the design of the 4P system | ☐ | ☐ | ☐ | ☐ | ☐ |
| 7. I would imagine that most people would learn to use the 4P system very quickly | ☐ | ☐ | ☐ | ☐ | ☐ |
| 8. I find the 4P system very cumbersome to use | ☐ | ☐ | ☐ | ☐ | ☐ |
| 9. I feel very confident using the 4P system | ☐ | ☐ | ☐ | ☐ | ☐ |
| 10. I needed to learn a lot of things before I could competently use the 4P system | ☐ | ☐ | ☐ | ☐ | ☐ |
| *Additional feedback or comments (optional):* | | | | | |

**Thank you for taking the time to complete this questionnaire**

## Appendix 7: Standard Operating Procedures (SOPs)

Three examples of SOPs used in the 4P study are presented in the following pages

- 001 How participants are taught to use the correct use of all the equipment
- 016 Extraction of patient notes onto the database
- 017 Measurement of each vital sign by midwives

## Appendix 8: Timeline

- 1st August 2012: Stage 1 of 4P study commences (as a sub-study of the Interbio-21st study, amendment approval 6th June 2012).
- 8th July 2015: Stage 2 of 4P study commences (Ethical approval 1st May 2015)
- 31st December 2016: full recruitment.
- Expected study completion (last vital sign data set submitted) September 2017

| | |
|---|---|
| **SOP Number** | **001-V1** |
| **SOP Title** | **Teaching Kit Use To 4P Participants** |

| | NAME | TITLE | SIGNATURE | DATE |
|---|---|---|---|---|
| **Author** | **Jude Kemp** | **Research Midwife** | | **27/02/2013** |
| **Reviewer** | | | | |
| **Authoriser** | | | | |

| | |
|---|---|
| **Effective Date:** | |
| **Review Date:** | |

| **READ BY** | | | |
|---|---|---|---|
| **NAME** | **TITLE** | **SIGNATURE** | **DATE** |
| | | | |
| | | | |
| | | | |
| | | | |
| | | | |
| | | | |


© Copyright: The University of Oxford 2009

## 1. PURPOSE

The purpose of this SOP is to describe a standardised method of teaching the use of the 4P home monitoring equipment.

Training all participants to the same standard will help eliminate user error as much as possible and ensure a high quality data set.

## 2. INTRODUCTION

The 4P study aims to capture 14 days' worth of physiological readings from participating women using a standardised home monitoring equipment kit. The readings consist of resting pulse oximetry, resting blood pressure, pulse and temperature.

The readings are collected onto a tablet computer by the participant and transmitted to the study database (hosted on a secure NHS server) at the end of each recording session. At the end of the 14 days, any un-submitted data are retrieved by 4P engineers and added to the study database for analysis. The data will be used to devise an obstetric early warning system.

It is important to ensure that the 4P participants are confident in using their home monitoring equipment before the start of the home monitoring phase.

The ideal teaching opportunity is at the participant's final two INTERBIO visits at approximately 36 and 38 weeks gestation.

If this is not possible, or the participant delivers prior to these appointments, the teaching should take place prior to discharge from hospital.

## 3. SCOPE

This SOP applies only to the 4P research midwives teaching the use of home monitoring equipment.

## 4. DEFINITIONS

The 4P home monitoring equipment consists of the following items:

- Tablet computer (Samsung Galaxy Tab) with charger and charging lead.
- Pulse oximeter (Nonin 3150 WristOx) with finger sensor (To ease understanding, it will be referred to as a finger meter)
- Thermometer (Genius 2) with a supply of lens caps (36 lens caps per unit)
- Blood pressure monitor (Microlife 3BTO-A(2)) with appropriate size cuff.
- 4P study instruction manual for parents describing set up of equipment.


© Copyright: The University of Oxford 2009

## 5. RESPONSIBILITIES

The 4P midwife is responsible for teaching the correct use of the home monitoring equipment and ensuring the participant feels confident in its use.

## 6. SPECIFIC PROCEDURE

**6.1** Before teaching the use of the home monitoring equipment, the 4P midwife should confirm that the 4P participant is still willing to take part in the 4P study. Where possible, arrange for the participant's partner or relative to be present and teach him/her the use of the equipment too.

**6.2** Teaching should take place in a private room if possible to provide a calm environment for learning and asking questions. If a private room is not available (e.g. on a ward) the curtains should be drawn around the bed to provide privacy.

**6.3** The 4 P midwife must explain the rationale behind the data collection and give the participant an opportunity to ask questions.

**6.4** The 4P midwife will teach the use of the following:

a. Pulse oximeter

b. blood pressure cuff positioning and measurement

c. thermometer use

**To ensure the participant has rested prior to the blood pressure reading, it is important that the recordings are always carried out in the following order:**

**Pulse Oximetry, Blood Pressure, Temperature**


© Copyright: The University of Oxford 2009

### a. Demonstration of correct pulse oximeter use:

Tell the participant:

*It is important that the following advice is always followed in order to obtain a correct reading.*

**Ask the participant to**

- Switch on the Nonin Pulse oximeter and place the grey Nonin pulse oximeter finger meter over the index finger of the left hand with the cable side upper most.
- Place the oximeter on a flat surface and sit comfortably while it is recording.
- Refrain from speaking during the reading.

The pulse oximeter will not work if the participant is wearing false nails or dark nail varnish. Ask the participant to remove nail varnish. If she is wearing or intends to wear false nails, an ear clip will be supplied.

### b. Demonstration of correct blood pressure cuff positioning

The appropriate cuff size should be used:

Standard cuff bladder 12-26 cm for the majority of adult arms (arm circumference 33-50 cm)

Large cuff bladder 12-40 cm for obese arms (arm circumference > 50 cm)

Small cuff bladder 12-18 cm for lean adult arms (arm circumference 23-33 cm)

(Combined European Society/Nice/British Hypertension Society recommendations, 2012) http://www.bhsoc.org/frequently-asked-questions/#BPM4


© Copyright: The University of Oxford 2009

Tell the participant:

*It is important that the following advice is always followed in order to obtain a correct reading.*

### Ask the participant to

- Sit with her arm stretched out and supported, for example on the arm of a chair or a cushion.
- The feet should be flat on the floor.
- Sit quietly and relax.
- Wear clothing with a loose sleeve as she will need to push it up. If the sleeve is tight, ask the participant to slip her arm out of the sleeve.
- Do not push tight sleeves up, as she will not be able to position the cuff correctly.
- Slip her arm into the cuff with the black tubing at the front and move it up the arm, 3cm above the elbow.
- Fasten the Velcro and adjust the position of the cuff if needed.
- Press the 'on' button. Explain that the cuff will go tight, which may be slightly uncomfortable.
- Encourage the participant not to talk during the measurement.
- Enter the BP value on the tablet computer.

### IMPORTANT

- Explain that a high BP reading will cause a message to appear onscreen, asking her to repeat the measurement in half an hour.
- If the measurement is still high, a message will appear, asking her to access medical help via the Maternity Assessment Unit or Day Assessment Unit at the John Radcliffe Hospital or the Horton Hospital in Banbury. ( see SOP 005 for details)
- Explain to the participant that it is her responsibility to access medical assistance and that the data she is recording are NOT monitored by a doctor or midwife.


© Copyright: The University of Oxford 2009

### c. Demonstration of correct use of the thermometer

Tell the participant:

*It is very important that the following advice is always followed in order to obtain a correct temperature reading:*

### Ask the participant to:

- Lift the thermometer out of its cradle. See photo.

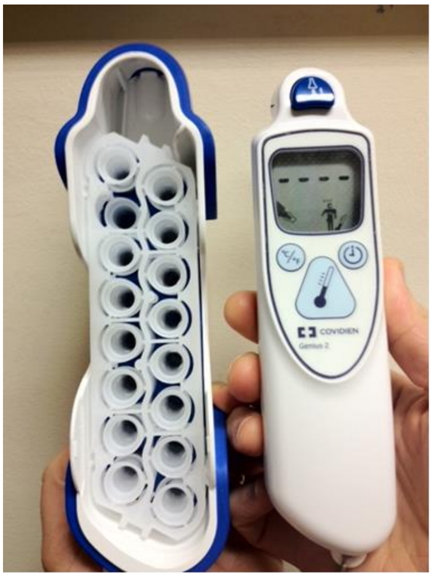


- Push the thermometer tip into a new lens cover from the pack, so that the lens cover clips onto the thermometer.
- Place the tip gently into the ear canal. See photo.

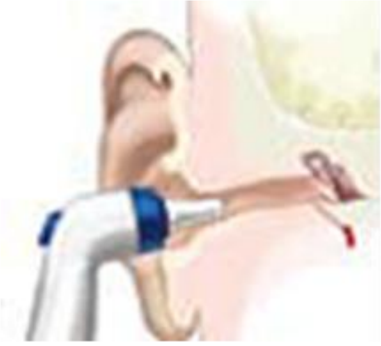

- Press the triangular button on the back of the thermometer.
- Wait for the triple beep before removing from the ear.
- Enter the number into the box on the tablet where indicated.


© Copyright: The University of Oxford 2009

## 7. EXTERNAL REFERENCES

http://www.bhsoc.org/frequently-asked-questions/#BPM4

## 8. CHANGE HISTORY

SOP No 001, version 1

Effective Date: see page 1

Initial Version

Previous version: N/A

| SOP no. | Effective Date | Significant Changes | Previous SOP no. |
|---|---|---|---|
| | | | |
| | | | |
| | | | |
| | | | |


© Copyright: The University of Oxford 2009

| | |
|---|---|
| **SOP Number** | **016-V1** |
| **SOP Title** | **Data Entry** |

| | NAME | TITLE | SIGNATURE | DATE |
|---|---|---|---|---|
| **Author** | Jude Kemp<br>Fiona Kumar | Data Entry | | |
| **Reviewer** | | | | |
| **Authoriser** | | | | |

| | |
|---|---|
| **Effective Date:** | |
| **Review Date:** | |

| **READ BY** | | | |
|---|---|---|---|
| **NAME** | **TITLE** | **SIGNATURE** | **DATE** |
| | | | |
| | | | |
| | | | |
| | | | |
| | | | |
| | | | |


© Copyright: The University of Oxford 2009

## 1. PURPOSE

The purpose of this SOP is to create clear guidelines on entering data from maternal anaesthetic charts, the partogram and MEOWS charts onto the 4P data base. This will help reduce errors in data entry and ensure high quality data is collected.

## 2. INTRODUCTION

The 4P study is collecting vital sign (blood pressure, pulse, oxygen saturations, temperature, and respiratory rate) readings from 1000 women throughout pregnancy, labour and the immediate post-partum period. Antenatally and postnatally the data are submitted to the database via a tablet computer.

The data from labour and delivery are manually entered by the research midwives onto the 4P study data base, copying each value form the handwritten charts used for vital sign recording.

When entering data manually from paper records onto an electronic database, there is scope for error by the person transcribing the data.

Some common causes of errors when transcribing data from paper charts onto electronic databases have been identified by Clifton and Watkinson (2015).

These are:

- The chart had illegible handwriting
- The incorrect row in the chart was transcribed
- The incorrect column in the chart was transcribed
- Vital signs were mismatched on the chart
- Typographical errors
- Errors of some other kind

Illegible handwriting was the most common cause of errors, with errors interpreting the grid like structure of the chart being second and third largest.

Whilst it is not possible to eliminate all errors occurring during the transcription of data, this SOP aims to minimise errors by describing in detail the methods used by the research midwives, with information on how to minimise the most commonly occurring errors described above.


© Copyright: The University of Oxford 2009

## 3. SCOPE

This SOP applies to all 4P Study midwives and research staff who will be entering data from charts used in labour and delivery onto the 4P database.

## 4. DEFINITIONS

Anaesthetic chart: A paper chart used by anaesthetists to document all maternal vital signs throughout surgical procedures

Partogram: A paper chart used by midwives to document the progress of labour, including timings of each stage of labour, rupture of amniotic membranes, cervical dilatation, fetal condition and maternal vital signs.

MEOWS (Modified Obstetric Early Warning System) chart: A paper chart used by midwives and doctors to document maternal vital signs during the in-hospital stay. The vital signs are assigned a score, action on these is decided according to this score.

## 5. RESPONSIBILITIES

It is the responsibility of each research midwife who will be entering data onto the 4P database to familiarise themselves with this SOP and follow the guidance in order to minimise. The SOP must be signed once it has been read.

## 6. SPECIFIC PROCEDURE

### 6.1 Entering an Anaesthetic chart:

On the anaesthetic chart, time is represented by boxes and columns. Each box represents five minutes, with each column representing 15 minutes.

Most anaesthetists enter blood pressure and pulse values onto the chart every 5 minutes.

Blood pressure readings are represented by arrow heads, a downward facing one for the systolic value, an upward facing one for the diastolic value.

Heart rate is represented by a dot on the relevant value.

Adapted from CTRG Template SOP Version 2.1
© Copyright: The University of Oxford 2009

When entering the readings from the chart, the starting time is identified (see photo)

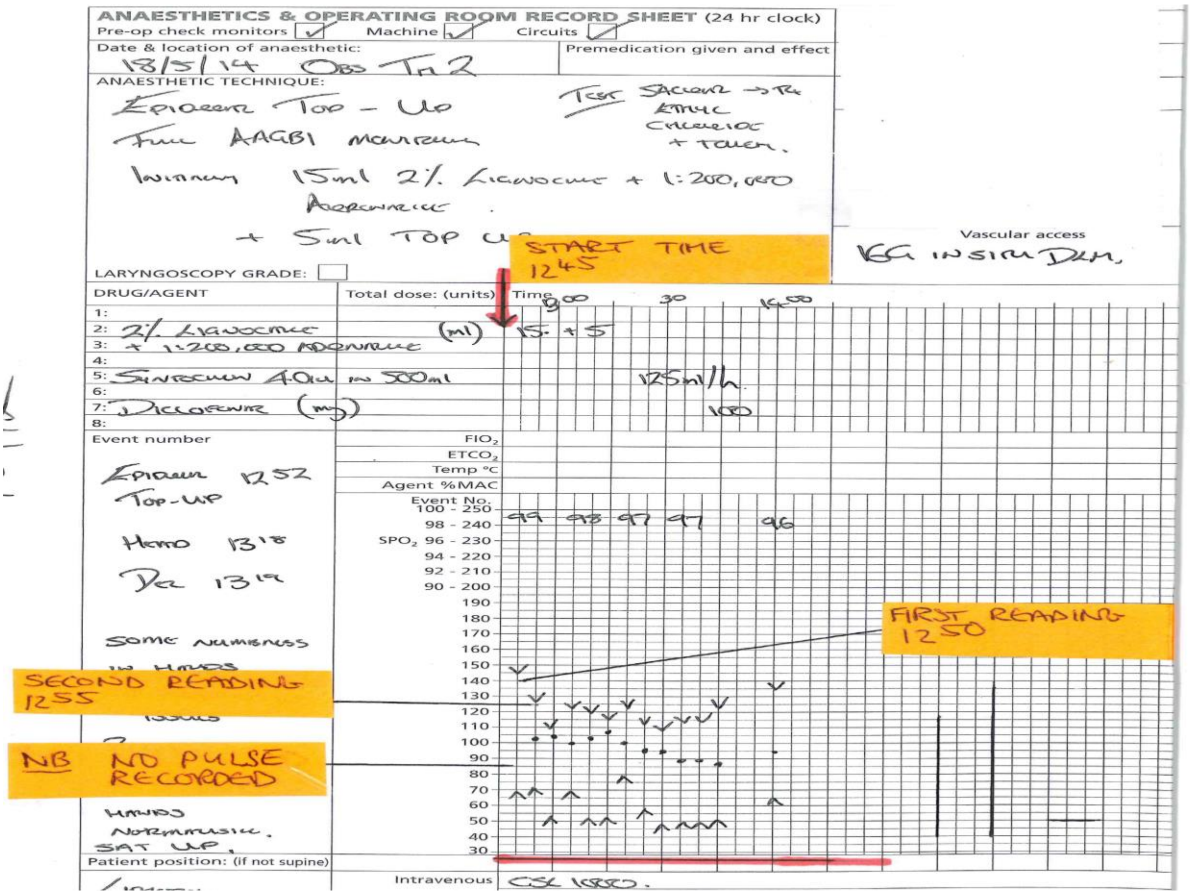


The start time of the chart may not be the time of the first reading to be entered, so it is important to ensure that the value is entered at the correct time, leaving a blank space if necessary.

On the above example, the start time is 12.45, but the first entry by the anaesthetist is 12.50. Readings are then entered every 5 minutes until 13.45, with one last one taken at 14.00.

Adapted from CTRG Template SOP Version 2.1
© Copyright: The University of Oxford 2009

The chart entry webpage through which the anaesthetic chart is entered (see image below) asks for the date and time to be entered first.

Next it asks for maternal heart rates. These are entered into each box at the corresponding time as seen on the chart above.

Note how the first column is left blank, as there is no reading for 12.45 and the first reading is entered at 12.50.

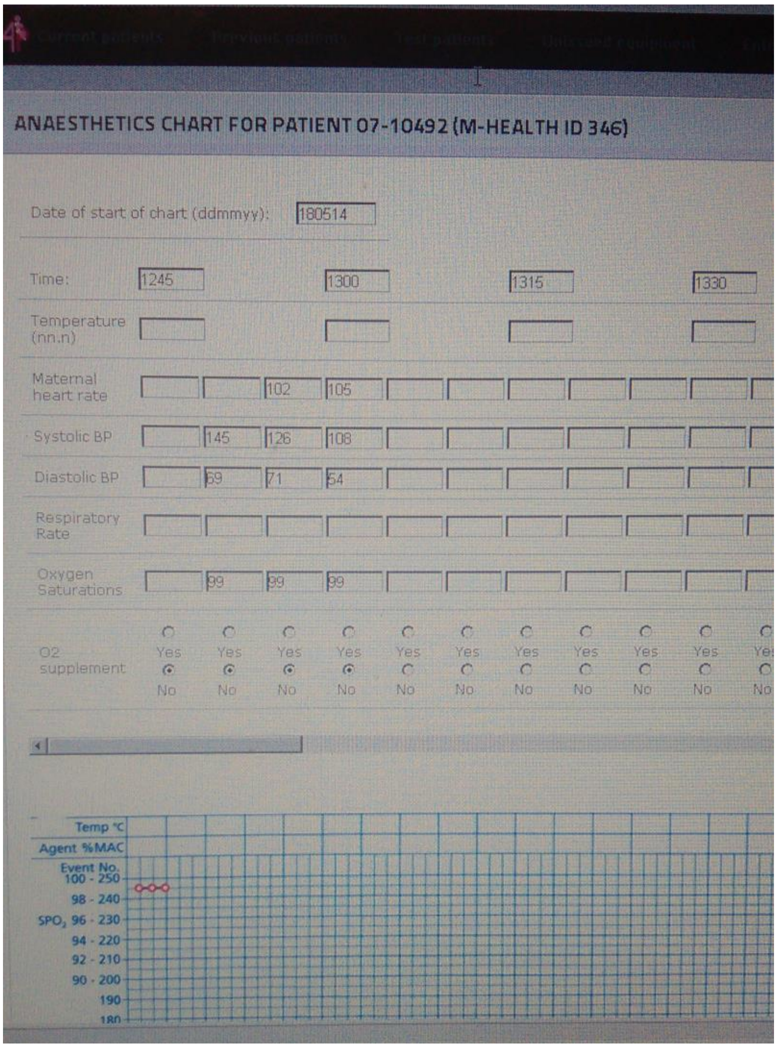


Adapted from CTRG Template SOP Version 2.1
© Copyright: The University of Oxford 2009

In order to avoid errors, it is easiest to enter all maternal pulses in sequence and then to move on to all systolic blood pressure values, before finishing off with the diastolic blood pressures.

The web page creates a visual representation, which aids with spotting errors as the data are entered (see picture below)

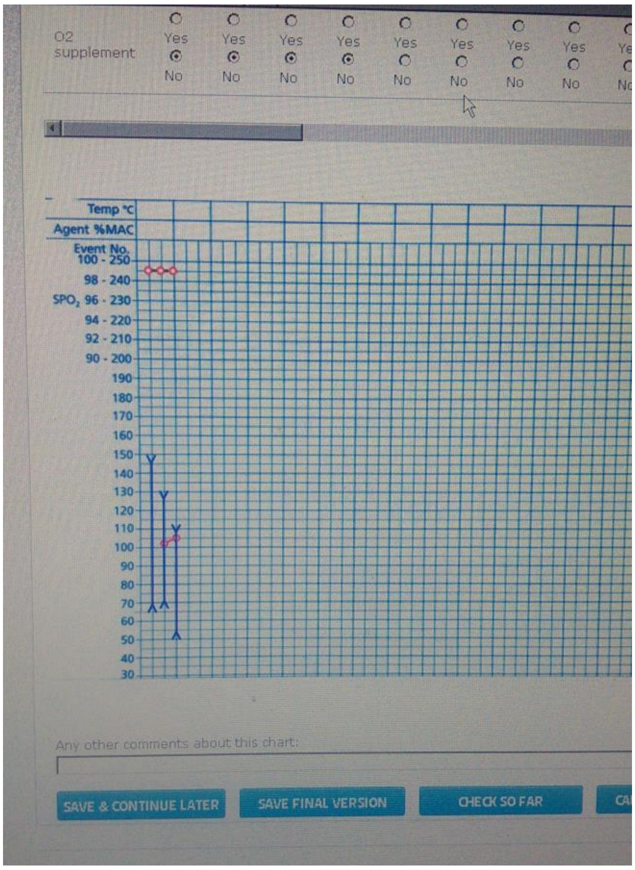


**COMMON ERRORS. WRONG COLUMN:**

On the above example, there is no pulse recorded at 12.50, so it is important to notice this and not enter the 12.55 pulse into the 12.50 box by mistake.

This section is the main text of the SOP. It details the procedure for the task to be performed.

There should be sufficient detail, clearly expressed, to enable a trained person to perform the procedure without supervision.

There should also be sufficient detail to enable a trained person to use the document to train others to perform the task.

The use of flow diagrams may be useful, especially in complex procedures.


© Copyright: The University of Oxford 2009

## 7. FORMS/TEMPLATES TO BE USED

Where Forms/Templates are referenced in the text, the numbers and titles are listed under this section.

## 8. INTERNAL AND EXTERNAL REFERENCES

This section is used to list all controlled internal references (e.g. SOPs) and external references referred to within the text of the SOP only.

### 8.1 Internal References

Insert relevant references as required, sufficient for the user to find the source document.

### 8.2 External References

Insert relevant references as required, sufficient for the user to find the source document. Web references should be included were possible.

## 9. CHANGE HISTORY

Where the SOP is the initial version:

- SOP No: Record the SOP and version number
- Effective Date: Record effective date of the SOP or "see page 1"
- Significant Changes: State, "Initial version" or "new SOP"
- Previous SOP no.: State "NA".

Where replacing a previous SOP:

- SOP No: Record the SOP and new version number
- Effective Date: Record effective date of the SOP or "see page 1"
- Significant Changes: Record the main changes from previous SOP
- Previous SOP no.: Record SOP and previous version number

| SOP no. | Effective Date | Significant Changes | Previous SOP no. |
|---|---|---|---|
| | | | |
| | | | |
| | | | |
| | | | |


© Copyright: The University of Oxford 2009

| | |
|---|---|
| **SOP Number** | **017-V1** |
| **SOP Title** | **Home Data Collection by 4P Midwife** |

| | NAME | TITLE | SIGNATURE | DATE |
|---|---|---|---|---|
| **Author** | **Jude Kemp**<br>**Fiona Kumar** | **Research Midwife** | | **15 April 2015** |
| **Reviewer** | | | | |
| **Authoriser** | | | | |

| | |
|---|---|
| **Effective Date:** | |
| **Review Date:** | |

**READ BY**

| NAME | TITLE | SIGNATURE | DATE |
|---|---|---|---|
| | | | |
| | | | |
| | | | |
| | | | |
| | | | |
| | | | |

Adapted from CTRG Template SOP Version 2.1
© Copyright: The University of Oxford 2009

## 1. PURPOSE

The purpose of this SOP is to ensure that the 4P midwives are carrying out home visits to 4P participants in a standardised way. This is to ensure that a full set of high quality data is collected.

## 2. INTRODUCTION

Research Midwives will be recording medical and obstetric history as well as measuring participant's heart rate, blood pressure, oxygen saturation, respiratory rate and temperature at the booking visit, follow-up visits and one week and two weeks post-delivery.

## 3. SCOPE

This SOP applies to the 4P research midwives visiting the 4P participants to obtain a full data set.

## 4. DEFINITIONS

Full data set taken by 4P midwife:

- Medical and obstetric history
- Blood pressure
- Pulse
- Temperature using 4P midwives' 'gold standard' thermometer (Genius 2)
- Pulse oximetry
- Respiration count using accelerometer in smart phone and chest wall movement count

## 5. RESPONSIBILITIES

It is the responsibility of the 4P midwives to become familiar with the content of this SOP as they will be recording data during home visits.


© Copyright: The University of Oxford 2009

## 6. SPECIFIC PROCEDURE

- Access the 4P website via 3/4G or wifi and open the electronic crf that corresponds with the participant's ID to complete the relevant medical and obstetric history sections
- Exit the application and open the android app for 4P data input and enter the participant's ID
- Connect the participant's finger to the pulse oximeter.
- Press the 'connect to finger probe' button on the app and ensure that the Nonin pulse oximeter is connected to the tablet.
- Explain to participant that her heart rate will be taken by palpating the radial pulse for 60 seconds.
- Palpate participant's radial pulse for 60 seconds and enter the measurement into the appropriate box on the app.
- Explain that the respiratory rate will be obtained using a smart phone placed on the participant's chest.
- Open the breathing rate app on the smart phone, enter the participant ID, attach the smart phone around the participant's neck and ask her to sit back in a chair so that the phone is resting on her chest.
- Ask participant to refrain from speaking during the measurement.
- Press the 'start counting breaths' button on the smartphone. A bleep will be heard.
- Wait for a second bleep. The word 'count' will appear on the screen. Press the 'count respirations' button on the tablet.
- For every breath, at the peak of inspiration, press the on-screen button on the tablet. The count takes 60 seconds.
- Count the participant's chest wall movements until a third bleep is heard from the smartphone and the word 'recording' appears on the screen.
- Press 'stop recording' on the screen and remove the smart phone from the participant's neck.
- Take the participant's temperature using the 4P Genius thermometer and enter it onto the tablet
- Take the participant's blood pressure;


© Copyright: The University of Oxford 2009

The appropriate cuff size should be used:

Standard cuff bladder 12-26 cm for the majority of adult arms (arm circumference 33-50 cm)

Large cuff bladder 12-40 cm for obese arms (arm circumference > 50 cm)

Small cuff bladder 12-18 cm for lean adult arms (arm circumference 23-33 cm)

(Combined European Society/Nice/British Hypertension Society recommendations, 2012) http://www.bhsoc.org/frequently-asked-questions/#BPM4

Ask the participant to

- Sit with her arm stretched out and supported, for example on the arm of a chair or a cushion.
- The feet should be flat on the floor.
- Sit quietly and relax.
- Wear clothing with a loose sleeve as she will need to push it up. If the sleeve is tight, ask the participant to slip her arm out of the sleeve.
- Do not push tight sleeves up, as she will not be able to position the cuff correctly.
- Slip her arm into the cuff with the black tubing at the front and move it up the arm, 3cm above the elbow.
- Fasten the Velcro and adjust the position of the cuff if needed.
- Press the ‘on’ button. Explain that the cuff will go tight, which may be slightly uncomfortable.
- Encourage the participant not to talk during the measurement.
- Enter the BP value on the tablet computer.

**IMPORTANT**

Explain that a high BP reading will cause a message to appear onscreen, asking her to repeat the measurement.

If the measurement is still high, a message will appear, asking her to access medical help

Explain to the participant that it is her responsibility to access medical assistance and that the data she is recording are NOT monitored by a doctor or midwife.


© Copyright: The University of Oxford 2009

- Enter the respiration count onto the tablet.
- Explain to the participant that the measurements will be taken a second time and carry them out as above, beginning with the palpation of the radial pulse.

## 7. CHANGE HISTORY

Where the SOP is the initial version:

- SOP No: 0017
- Effective Date: see page 1
- Significant Changes: Initial version
- Previous SOP no.: NA

| SOP no. | Effective Date | Significant Changes | Previous SOP no. |
|---|---|---|---|
| | | | |
| | | | |
| | | | |
| | | | |

Adapted from CTRG Template SOP Version 2.1
© Copyright: The University of Oxford 2009
