# Supplementary material for: Pregnancy physiology pattern prediction study (4P study): protocol of an observational cohort study collecting vital sign information to inform the development of an accurate centile-based obstetric early warning score
Source: BMJ Open. 2017 Sep 1;7(9):e016034. doi: 10.1136/bmjopen-2017-016034 (PMC5589023; doi:10.1136/bmjopen-2017-016034)
Supplement: Author's manuscript [file bmjopen-2017-016034.draft_revisions.pdf]

BMJ Open is committed to open peer review. As part of this commitment we make the peer review history of every article we publish publicly available.

When an article is published we post the peer reviewers' comments and the authors' responses online. We also post the versions of the paper that were used during peer review. These are the versions that the peer review comments apply to.

The versions of the paper that follow are the versions that were submitted during the peer review process. They are not the versions of record or the final published versions. They should not be cited or distributed as the published version of this manuscript.

BMJ Open is an open access journal and the full, final, typeset and author-corrected version of record of the manuscript is available on our site with no access controls, subscription charges or pay-per-view fees (http://bmjopen.bmj.com).

If you have any questions on BMJ Open's open peer review process please email editorial.bmjopen@bmj.com

BMJ Open

The Pregnancy Physiology Pattern Prediction Study (4P Study): protocol of an observational cohort study collecting vital sign information to inform the development of an accurate centile-based obstetric early warning score

Journal:	*BMJ Open*
Manuscript ID	bmjopen-2017-016034
Article Type:	Protocol
Date Submitted by the Author:	27-Jan-2017
Complete List of Authors:	Kumar, Fiona; Oxford University Hospitals NHS Foundation Trust, Nuffield Department of Obstetrics and Gynaecology<br>Kemp, Jude; Oxford University Hospitals NHS Foundation Trust, Nuffield Department of Obstetrics and Gynaecology<br>Edwards, Clare; Oxford University Hospitals NHS Foundation Trust, Nuffield Department of Obstetrics and Gynaecology<br>Pullon, Rebecca; University of Oxford, Engineering Science<br>Loerup, Lise; University of Oxford, Engineering Science<br>Triantafyllidis, Andreas; University of Oxford, Engineering Science<br>Salvi, Dario; University of Oxford, Engineering Science<br>Gibson, Oliver; University of Oxford, Engineering Science<br>Gerry, Stephen; University of Oxford, Centre for Statistics in Medicine<br>MacKillop, Lucy; Oxford University Hospitals NHS Trust, Nuffield Department of Obstetrics & Gynaecology<br>Tarassenko, Lionel; University of Oxford, Engineering Science<br>Watkinson, Peter; Oxford University Hospitals NHS Trust, Nuffield Department of Clinical Neurosciences
**Primary Subject Heading**:	Obstetrics and gynaecology
Secondary Subject Heading:	Evidence based practice
Keywords:	pregnancy, vital signs, early warning score

SCHOLARONE™ Manuscripts

The Pregnancy Physiology Pattern Prediction Study (4P Study): protocol of an observational cohort study collecting vital sign information to inform the development of an accurate centile-based obstetric early warning score

Fiona Kumar[1], Jude Kemp[1], Clare Edwards[1], Rebecca M. Pullon[2], Lise Loerup[2], Andreas Triantafyllidis[2], Dario Salvi[2], Oliver Gibson[2], Stephen Gerry[3], Lucy H. Mackillop[1], Lionel Tarassenko[2] and Peter J. Watkinson[4]

[1]Nuffield Department of Obstetrics & Gynaecology, Oxford University Hospitals NHS Trust, Oxford, UK

[2]Department of Engineering Science, University of Oxford, Oxford, UK

[3]Centre for Statistics in medicine, Nuffield Department of Orthopaedics, Rheumatology and Musculoskeletal Sciences, University of Oxford, Oxford, UK

[4] Kadoorie Centre for Critical Care Research and Education, Nuffield Department of Clinical Neurosciences, Oxford University Hospitals NHS Trust, Oxford, UK

Corresponding author:

Rebecca M. Pullon

Postal address: Institute of Biomedical Engineering, Old Road Campus Research Building, Off Roosevelt Drive, Headington, Oxford, OX3 7DQ, UK

Email: Rebecca.pullon@eng.ox.ac.uk

Phone: 01865 617720

Key words: pregnancy, vital signs, early warning score

Word count: 3300 (main text, excluding references and appendices)

For peer review only

ABSTRACT

Introduction: Successive Confidential Enquiries into Maternal Deaths in the UK have identified an urgent need to develop a national Early Warning Score (EWS) specifically for pregnant or recently pregnant women to aid more timely recognition, referral and treatment of women who are developing life-threatening complications in pregnancy or the puerperium. Although many local EWS are in use in obstetrics, most have been developed heuristically. No current obstetric EWS has defined the thresholds at which an alert should be triggered using evidence-based normal ranges, nor do they reflect the changing physiology that occurs with gestation during pregnancy.

Methods and analysis: An observational cohort study involving 1000 participants across three UK sites in Oxford, London and Newcastle. Pregnant women will be recruited at approximately 14 weeks gestation and have their vital signs (heart rate, blood pressure, respiratory rate, oxygen saturation and temperature) measured at 4-6 week intervals during pregnancy. Vital signs recorded during labour and delivery will be extracted from hospital records. After delivery, participants will measure and record their own vital signs daily for two weeks. During the antenatal and postnatal periods, vital signs will be recorded on an Android tablet computer through a custom software application and transferred via mobile internet connection to a secure database. The data collected will be used to define reference ranges of vital signs across normal pregnancy, labour and the immediate post-natal period. This will inform the design of an evidence-based obstetric early warning score.

Ethics and dissemination: The study has been approved by the NRES committee South East Coast – Brighton and Sussex (14/LO/1312) and is registered with the ISRCTN (10838017). All participants will provide written informed consent and can withdraw from the study at any point. All data collected will be managed anonymously. The findings will be disseminated in international peer-reviewed journals and through research conferences.

For peer review only

STRENGTHS AND LIMITATIONS OF THIS STUDY

- This will be the first study to describe normal ranges of all five vital signs (blood pressure, heart rate, temperature, respiratory rate and oxygen saturation) gathered contemporaneously across gestation in the antepartum, intrapartum and in the postpartum periods.
- Participants will all be within UK healthcare systems. This may limit translation to other healthcare settings.

For peer review only

BACKGROUND

Early warning scoring systems (EWS) for obstetric patients assign increasing scores (or colours) to each individual physiological measurement (blood pressure, heart rate, temperature, respiratory rate and oxygen saturation) as they become more abnormal. If either the sum of scores for several physiological measurements, or a score for a single physiological measurement exceed set thresholds, an alert is triggered. Successive Confidential Enquiries into Maternal Deaths in the UK have highlighted an urgent need to develop a national obstetric EWS to aid the more timely recognition, treatment and referral of women who are developing life-threatening complications of pregnancy[1, 2, 3]. This recommendation was strongly supported by a national survey of obstetric anaesthetists[4]. It is acknowledged that recognising physiological deterioration is complicated by the normal changes in maternal physiology that occur both during pregnancy and immediately after delivery[2].

An evidence-based approach is required for widespread acceptance of a national obstetric early warning scoring system[5]. Recognising normality is the first step to understanding abnormality[6]. To understand normal ranges at different gestational ages of "normal" pregnancy, the intrapartum and the post-partum period, large numbers of measurements at each point are required. These will allow normal ranges and their associated centiles throughout pregnancy to be defined in the modern era. It is important that these observations are measured at the same time to allow their interactions to be understood. Longitudinal data recorded from the same patients, where possible with the same equipment, across the entire pregnancy and the first two weeks after delivery is particularly important to ensure that step-changes between the stages of pregnancy are not missed.

Large amounts of longitudinal data for blood pressure during pregnancy already exist (for example from the longitudinal AVON[7] and BOSHI[8] studies). However much of this is now more than two decades old. We found less data for heart rate in pregnancy (for example[9, 10, 11]). Relatively few data have been collected during the intrapartum period or for the first two weeks postpartum (for example if postnatal data are collected, this only occurs at one or two occasions several weeks after delivery, such as at six weeks in[12, 13], and 6 months in[14]). Furthermore, there are very few data available for temperature (for example[15, 16]), respiratory rate (for example[17, 18]) and oxygen saturation (for example[17, 19]) in pregnancy, intrapartum or postpartum, from which to define modern normal-range centiles. The data that are available suggest clear trends occur during pregnancy which may need to be taken into account to ensure appropriate identification of abnormality. For example systolic blood pressure appears to change by around 6mmHg over the course of pregnancy[7, 8]. However, while clear vital sign trends are apparent, the range of expected vital sign values during pregnancy are not known, and have not been able to be determined by synthesis of available data sets.

For peer review only

A centile-based approach to identifying abnormality will allow experts to choose which women should be reviewed, based on strong evidence of how far one or more of their vital-sign observations lie from normality. The approach is particularly suited to the analysis of data recorded during pregnancy, as well as intrapartum and postpartum, during which the event rate is relatively low. We have previously used such an approach for both the recognition of physiological deterioration in hospitalised adults [6] and the maintenance of safety in the aerospace industry [20].

The primary objective of this study is to develop a database of vital sign measurements from pregnancy, labour and the postpartum period (Table 1). Estimates of population distributions and associated centiles can be derived from this database. The secondary objective of this study is to use this information to develop a centile-based early warning scoring system for pregnancy, labour and the postpartum period, that will guide clinical experts to provide the most appropriate care.

	**Objectives**	**Endpoints**
**Primary**	• To develop a database of vital sign measurements during pregnancy, labour and the postpartum period	• Estimates of distributions and associated centiles for five vital signs throughout pregnancy, labour and the postpartum period
**Secondary**	• To develop a centile-based early-warning scoring system for pregnancy, labour and the post-partum period<br>• To investigate new patterns within vital sign data in pregnancy	• Centile-based thresholds for alerting for five vital signs throughout pregnancy, labour and the postpartum period<br>• Trend analyses of five vital signs and inter-relations between these trends

Table 1: A summary of the primary and secondary objectives and endpoints

For peer review only

METHODS AND ANALYSIS

We prepared this protocol following the Strobe and SPIRIT guidelines, where appropriate.

Study design: A UK three-centre longitudinal observational study. We will commence the study in August 2012. We will continue the study until 1000 women contribute vital sign data and the last participant is 14 days post-partum. Women will be approached to take part when they attend the ultrasound department or antenatal clinics at three university hospitals (Appendix 1). In Oxford, women may be approached when they are recruited to take part in the Interbio study[21].

Study population: We aim to recruit 1000 women aged 16 or over, with a singleton pregnancy of less than 20 weeks gestation, who fall within category 1 of the American Society of Anaesthesiologists' classification of physical status at enrolment ("A normal healthy patient without any clinically important co-morbidity and without a clinically significant past/present medical history"). Gestational age was determined using a measurement of crown-rump length obtained prior to 14 weeks of gestation[22]. We will exclude women with a known condition expected by the recruiting clinician to alter maternal vital signs. The full inclusion and exclusion criteria are detailed in Appendix 2.

Vital sign data collected: Five vital signs will be collected: blood pressure, heart rate, oxygen saturation, temperature and respiratory rate. Blood pressure will be measured with a blood pressure monitor approved for use in pregnancy (WatchBP Home, Microlife, Taipei, Taiwan). Heart rate and oxygen saturation will be measured with a Bluetooth-enabled pulse oximeter (WristOx2 3150, Nonin Medical, Minnesota, USA). Temperature is measured with a tympanic thermometer (Genius™ 2, Covidien (Medtronic), Dublin, Ireland). In a subgroup, temperature will be measured with an additional Bluetooth thermometer (Fora IRb 20b, ForaCare, Taipei, Taiwan). The device allows for automatic transfer of readings (thus eliminating the risk of user input errors) but is not commonly used in UK clinical practice. Trained midwives will estimate respiratory rate by observing chest wall movement following a standard operating procedure. A software application on an Android smartphone will also be used to estimate respiratory rate through the detection of chest-wall movements with the in-built accelerometer and gyroscope. The equipment used for data collection is shown in Figure 1.

Vital sign data will be entered (or in the case of Bluetooth-enabled devices, automatically transmitted), onto a tablet computer (Samsung Galaxy Tab 4.0) and sent to a secure database on the NHS network using 3G/4G. The tablet computer will run a custom Android application that will guide the user through the measurement sequence. Once sent, the data will be automatically deleted from the tablet. All data will be entered, transferred and stored using a unique study number.

For peer review only

Vital sign data recorded during delivery will be extracted retrospectively from the participants' hospital notes once they have been discharged from hospital. All vital signs recorded on existing obstetric EWS charts, anaesthetic charts and labour charts (partogram) will be entered on to the secure 4P study database for analysis. Examples of the intrapartum case report form are included in Appendix 3.

Other data collected: In addition, relevant medical and obstetric history will be extracted from the participants' notes to support analysis of the data. We will collect demographic information (age, height, weight, ethnicity, number of previous pregnancies, smoking status), medical and obstetric history, current health status, pregnancy-related health and current medications, at the initial assessment. At each follow up appointment we will collect assessments of smoking status, current health status, pregnancy-related health and current medications. The information will be collected onto standardized case report forms (for example, see Appendix 4). Where required, our definitions of variables included in the 4P study are detailed in Appendix 5.

At the discharge appointment, participants will be asked to fill out the standardised System Usability Survey (SUS)[23] to assess the usability of the 4P mobile system for home monitoring (Appendix 6).

Follow-up: In the antenatal period, the participants will be followed up at four to six weekly intervals. If they are co-enrolled in the Interbio study, antenatal data will be collected by Interbio staff. During the immediate postpartum period (days 0-14 after delivery), participants will be asked to undertake daily home monitoring. Additional postnatal data will be collected by research midwives on 2-3 occasions. If participants do not respond to contact by research midwives during the study, they will be treated as withdrawn after the third failed contact attempt. Data already collected from withdrawn participants will be included in the final analysis, unless explicitly requested otherwise. If participants complete the study they will receive a £20 gift voucher. A participant timeline for the study is shown in Figure 2.

Personnel: Trained research midwives will undertake all antenatal observation sets, and 1-3 postnatal observation sets, following study Standard Operating Procedures (SOPs) (for example, see Appendix 7). Research midwives will provide training in the use of home monitoring equipment at a suitable time around delivery, as described by SOPs. If abnormal vital signs are identified at the home visits, the research midwife will refer the participant to her usual midwife, GP or local maternity hospital assessment unit. All participants will be made aware that the information collected during the home-monitoring phase is not reviewed in real-time and that it remains their responsibility to seek assistance if they feel unwell. The research midwives will extract all hospital data from the notes and observation charts into an electronic study database, following study SOPs. Research midwives will perform frequent site visits to carry out midwife training and address any recruitment and equipment issues.

For peer review only

Sample size: In order to determine the sample size we considered the precision of the data in estimating specific centiles. We assumed data are normally distributed at each gestational age. The standard error of the Pth centile is given using the standard formula[24]:

$$SE_P = SD\sqrt{\left(1+\frac{1}{2}Z_P^2\right)/n}$$

where SE is the standard error, SD is the standard deviation of the measurement of interest (which may change according to gestational age), Z_p is the value of the standard normal distribution corresponding to the Pth centile, and n is the sample size. To create an evidence based early warning score we desired a 95% confidence interval with a standard error of less than 0.10*SD at the boundaries. A sample size of 1000 women will achieve a standard error of 0.05*SD at the 2.5th and 97.5th centiles, and even greater precision at the less extreme centiles. Adequate precision is also met for any subgroup analysis; for example a sample size of 300 women will achieve a standard error of 0.1*SD at the 2.5th and 97.5th centiles. We have been conservative in these estimates as we have not considered the effect of serial measurements from the same women[25]. Using Royston's design factor of 2.3, a longitudinal study of 1000 women could have equivalent precision to a cross sectional study of around 2300 women[26].

Data Management: A custom designed website will be the main portal for manual data entry and vital sign review (Figure 3). Access will be restricted by username and 8 digit password to staff directly involved in the study. The 4P database and interfaces will be held within a secure virtual server protected with an NHS network firewall. For manual chart entry, automatic checks will be performed to ensure sequential chart entry and reasonable vital sign values.

Data quality: Duplicate measurements will be recorded for a subset of participant visits to document the effect of using only the first measurement (as in clinical practice). To minimise participant inconvenience, duplicate measurements will be reviewed by study statisticians to decide when an adequate number of duplicate measurements have been collected. Research midwives will undertake observation sets in the home to allow comparison between participant-taken and midwife-taken observations.

Temperature will be measured with both Bluetooth enabled and non-Bluetooth enabled devices to allow method comparison. Quality of the pulse oximeter recordings (heart rate and oxygen saturation) will be assessed in real time by observation of the photoplethysmograph waveform (only visible to midwives). Additionally, the electronic heart rate measurement will be checked with a 60 second pulse palpitation[27].

A small proportion of notes will be dual entered at site visits to check error rates.

For peer review only

Data analysis: Statistical methods to generate gestational age-specific centiles for maternal vital signs will closely follow those methods described and used in the INTERGROWTH-21st Project for foetal growth measurements[28]. Pre-defined exclusion criteria will be applied to make sure only those with a normal pregnancy are included in the analysis. We will explore different statistical methods in order to achieve the best fit to the data, whilst being as simple as possible. In brief, these methods will include: mean and SD using fractional polynomials[29]; Cole's lambda, mu and sigma (LMS) method[30]; the lambda, mu, sigma and Box-Cox t distribution (LMST) method[31]); the lambda, mu, sigma and Box-Cox power exponential (LMSP) method[32]; and multi-level models. To present curves, we will use different smoothing techniques including: fractional polynomial[32]; cubic splines[33], and penalised splines[34]. The INTERGROWTH-21st Project concluded that there was no evidence of a non-normal distribution and therefore no benefit of the more complex modelling techniques and opted to use the mean and SD method[28]. We therefore anticipate that values of each vital sign will be normally distributed at each stage of pregnancy, and that we will also be able to choose the simpler mean and SD method, as the best option. In this case, we will build the model in a multi-level framework, taking account of the correlation in repeated measures within participants.

Goodness of fit will be assessed by visual inspection of empirical centiles versus fitted centiles, quantile-quantile plots of the residuals, plots of residuals versus fitted values, and the distribution of fitted Z scores across gestational ages.

It is expected that some participants will become lost to follow-up or will have some missing measurements. These participants will still be included in the analysis, unless all data are missing. Missing data will be implicitly imputed using the multilevel modelling techniques under the missing at random assumption (MAR).

Centiles will be derived in the following pre-defined subgroups: BMI, maternal age, parity, smoking status, ethnicity; and will be visually compared to assess differences between subgroup strata. Sensitivity analyses will be performed to assess the robustness of the final model to the various assumptions made, including assumptions around missing data, and the choice of participants included in the analysis.

For peer review only

ETHICS AND DISSEMINATION

Ethical considerations: Informed written consent will be sought for all participants and participants can withdraw from the study at any time. The study is non-invasive and presents no significant risk to participants. Data are managed in accordance with the Data Protection Act (1998).

Participants in the 4P study will be observed more frequently than they would be as per usual maternity care. However, the opportunity to meet with health professionals more frequently could be considered beneficial. During home visits, either antenatally or postnatally, the research midwife will refer participants to their usual care team if any abnormal vital sign values are recorded. Participants are asked to monitor their own vital signs at home. There is the possibility that some measurements are abnormal. If elevated blood pressure values are recorded, the software on the Android tablet advise study participants to contact their local maternity unit (a list of telephone numbers is provided). Participants are advised that the research team does not monitor the data sent from home and that they are responsible for acting on the software instructions whenever any abnormally high or low values of vital signs are recorded.

Dissemination: Results from the study will be disseminated through conferences and published in international peer-reviewed journals. Participants will be able to access results through a link on the OSPREA (Oxford Safer Pregnancy Alliance) website. Important protocol modifications will be communicated on the ISRCTN where the study is registered (No. 10838017). The data will be analysed by the University of Oxford (Institute of Biomedical Engineering) in the first instance. Anonymised data will be made available to other researchers on application.

CONCLUSIONS

An evidence-based early warning system will enable clinicians to identify more quickly the ill or deteriorating woman in a hospital setting at any time during pregnancy, delivery and the immediate post-partum period. Currently there are a number of EWS charts for pregnant women being used in the UK with different thresholds for alerts, none of which are evidence-based. The 4P study will enable a standardised evidence-based obstetric EWS chart to be developed from a database of vital signs for gestation of pregnancy, delivery and two weeks postpartum.

For peer review only

Funding: The 4P study is supported by Biomedical Informatics & Technology (BIT) theme of the NIHR Oxford Biomedical Research Centre. RP and LL acknowledge the support of the RCUK Digital Economy Programme grant number EP/G036861/1 (Oxford Centre for Doctoral Training in Healthcare Innovation). LL is also supported by the Clarendon Fund.

Study sponsor: University of Oxford. Contact Heather House, Joint Research Office, Block 60, Churchill Hospital, Old Road, Oxford, OX3 7LE. Email: ctrg@admin.ox.ac.uk.

Competing interests: None.

Ethics approval: NRES committee South East Coast – Brighton and Sussex (Ref: 14/LO/1312)

Contributors: PW is the principal investigator. PW, LM, LL, LT, OG, JK and FK made substantial contributions to the conception and design of the project. SG provided advice on statistical methodology. JK, FK and CE are involved in data collection. OG, AT, DS, LL and RP are involved in software design and data analysis. FK wrote the first draft of the present manuscript. RP and PW reviewed and updated the draft. All authors read and approved the final manuscript.

Figure captions:

Figure 1: Equipment used for vital sign data collection

Figure 2: Timeline of follow up visits during the antenatal and postnatal periods.

Figure 3: Review of vital sign data for participant 0009 on the 4P website (example).

For peer review only

REFERENCES

1. Saving Mothers' Lives: Reviewing maternal deaths to make motherhood safer - 2003-2005. The Seventh Report of the Confidential Enquiries into Maternal Deaths in the United Kingdom. 2007.
2. Saving Mothers' Lives: Reviewing maternal deaths to make motherhood safer: 2006–2008. The Eighth Report of the Confidential Enquiries into Maternal Deaths in the United Kingdom. 2011.
3. Saving Lives, Improving Mothers' Care. Lessons learned to inform future maternity care from the UK and Ireland. Confidential Enquiries into Maternal Deaths and Morbidity 2009-2012
4. Swanton RD, Al-Rawi S, Wee MY. A national survey of obstetric early warning systems in the United Kingdom. *International Journal of Obstetric Anesthesia* 2009;18:253-7.
5. Smith GB, Prytherch DR, Meredith P, Schmidt PE, Featherstone PI. The ability of the National Early Warning Score (NEWS) to discriminate patients at risk of early cardiac arrest, unanticipated intensive care unit admission, and death. *Resuscitation* 2013;84:465-70.
6. Tarassenko L, Clifton DA, Pinsky MR, Hravnak MT, Woods JR, Watkinson PJ. Centile-based early warning scores derived from statistical distributions of vital signs. *Resuscitation* 2011;82:1013-8.
7. Macdonald-Wallis C, Lawlor DA, Fraser A, May M, Nelson SM, Tilling K. Blood pressure change in normotensive, gestational hypertensive, preeclamptic, and essential hypertensive pregnancies. *Hypertension* 2012;59:1241-8.
8. Magriples U, Boynton MH, Kershaw TS, Duffany KO, Rising SS, Ickovics JR. Blood pressure changes during pregnancy: impact of race, body mass index, and weight gain. *American Journal of Perinatology* 2013;30:415-24.
9. Mahendru AA, Everett TR, Wilkinson IB, Lees CC, McEniery CM. A longitudinal study of maternal cardiovascular function from preconception to the postpartum period. *Journal of Hypertension*, 2014;32(4):849-856. doi: 10.1097/HJH.0000000000000090
10. Gu H, Zhang S, Qiao Y, Luo Z, Zeng Y, Wang Q. A study of maternal hemodynamic change during healthy pregnancy and women with gestation hypertension. *Bio-Medical Materials & Engineering*. 2006;16(1):77-82.
11. van Oppen ACC, van der Tweel I, Alsback GPJ, Heetharr RM, Bruinse HW. A Longitudinal study of maternal hemodynamics during normal pregnancy. *Obstetrics and Gynecology*, 1996;88(1):40-46
12. Halligan A, O'Brien E, O'Malley K, Mee F, Atkins N, Conroy R, Walshe JJ, Darling M. Twenty-four-hour ambulatory blood pressure measurement in a primigravid population. *Journal of Hypertension* 1993;11:869-873
13. Blake MJ, Martin A, Manktelow BN, Armstrong C, Halligan AW, Panerai RB, et al. Changes in baroreceptor sensitivity for heart rate during normotensive pregnancy and the puerperium. *Clinical Science* 2000;98(3):259-68.
14. Grindheim G, Estensen ME, Langesaeter E, Rosseland LA, Toska K. Changes in blood pressure during healthy pregnancy: a longitudinal cohort study. *Journal of Hypertension* 2012;30(2):342-50.
15. Clapp JF, Capeless E. Cardiovascular Function before, during and after the first and subsequent pregnancies. *The American Journal of Cardiology* 1997;80:1469-1473.
16. Tuffnell DJ, Buchan PC, Albert D, Tyndale-Biscoe S. Fetal heart rate responses to maternal exercise, increased maternal temperature and maternal circadian variation. *Journal of Obstetrics and Gynaecology* 1990;10(5):387-91.
17. Templeton A, Kelman GR. Maternal blood-gasses, physiological shunt and VD/VT in normal pregnancy. *British Journal of Anaethesiology* 1976;48:1001-1004
18. Ekholm EMK, Erkkola RU, Piha SJ, Jaolnen JO, Metsala TH, Antila KJ. Changes in autonomic cardiovascular control in mid-pregnancy. *Clinical Physiology* 1992;12:527-536
19. Deckhardt R, Fembacher PM, Schneider KTM, Graeff H. Maternal arterial oxygen saturation during labour and delivery: Pain-dependent alterations and effects on the newborn. Obstetrics and Gynecology. 1987;70(1):21-5.
20. King S, Bannister PR, Clifton DA, Tarassenko L. Probabilistic Approaches to Condition Monitoring of Aerospace Engines. *Journal of Aerospace Engineering* 2009;223:533-41.
21. INTERBIO-21st, 2016. Retreived 15-06-2016 from www.interbio21.org.uk

For peer review only

22. Papageorghiou AT, Kennedy SH, Salomon LJ, Ohuma EO, Cheikh Ismail L, Barros FC, Lambert A, Carvalho M, Jaffer YA, Bertino E, Gravett MG, Altman DG, Purwar M, Noble JA, Pang R, Victoria CG, Bhutta ZA, Villar J. International standards for early fetal size and pregnancy dating based on ultrasound measurement of crown-rump length in the first trimester of pregnancy. *Ultrasound Obstetrics and Gynecology* 2014;44(6):641-648
23. Brooke. SUS – A quick and dirty usability scale. *Usability evaluation in industry* 1996;189(194):4-7
24. Healy MJ. Notes on the statistics of growth standards. *Annals of Human Biology* 1974;1(1):41-7
25. Altman DG, Ohuma EO. Statistical considerations for the development of prescriptive fetal and newborn growth standards in the INTERGROWTH-21st Project. *British Journal of Obstetrics and Gynaecology* 2013;120 Suppl 2:71-6
26. Royston P. Calculation of unconditional and conditional reference intervals for foetal size and growth from longitudinal measurements. *Statistics in Medicine* 1995;14(13):1417-36.
27. Royal College od Nursing. Standards for assessing, measuring and monitoring vital signs in infants, children and young people. Retrieved 12-09-2016 from: https://www2.rcn.org.uk/__data/assets/pdf_file/0004/114484/003196.pdf
28. Papageorghiou AT, Ohuma EO, Altman DG et al International standards for fetal growth based on serial ultrasound measurements: the Fetal Growth Longitudinal Study of the INTERGROWTH-21st Project. *Lancet* 2014;384:869-79
29. Royston P, Altman DG. Regression using fractional polynomials of continuous covariates: parsimonious parametric modelling. *Journal of the Royal Statistical Society* 1994;43:429–67.
30. Cole TJ, Green PJ. Smoothing reference centile curves: the LMS method and penalized likelihood. *Statistics in Medicine* 1992;11:1305–19.
31. Rigby RA, Stasinopoulos DM. Using the Box-Cox *t* distribution in GAMLSS to model skewness and kurtosis. *Statiscial Modelling* 2006;6**:** 209–29.
32. Rigby RA, Stasinopoulos DM. Smooth centile curves for skew and kurtotic data modelled using the Box–Cox power exponential distribution. *Statistics in Medicine* 2004;23:3053–76.
33. Green PJ, Silverman BW. Nonparametric regression and generalized linear models: a roughness penalty approach. London:Chapman & Hall, 1994.
34. Eilers PHC, Marx BD. Flexible smoothing with B-splines and penalties. *Statistical Science* 1996;11:89–158.

For peer review only

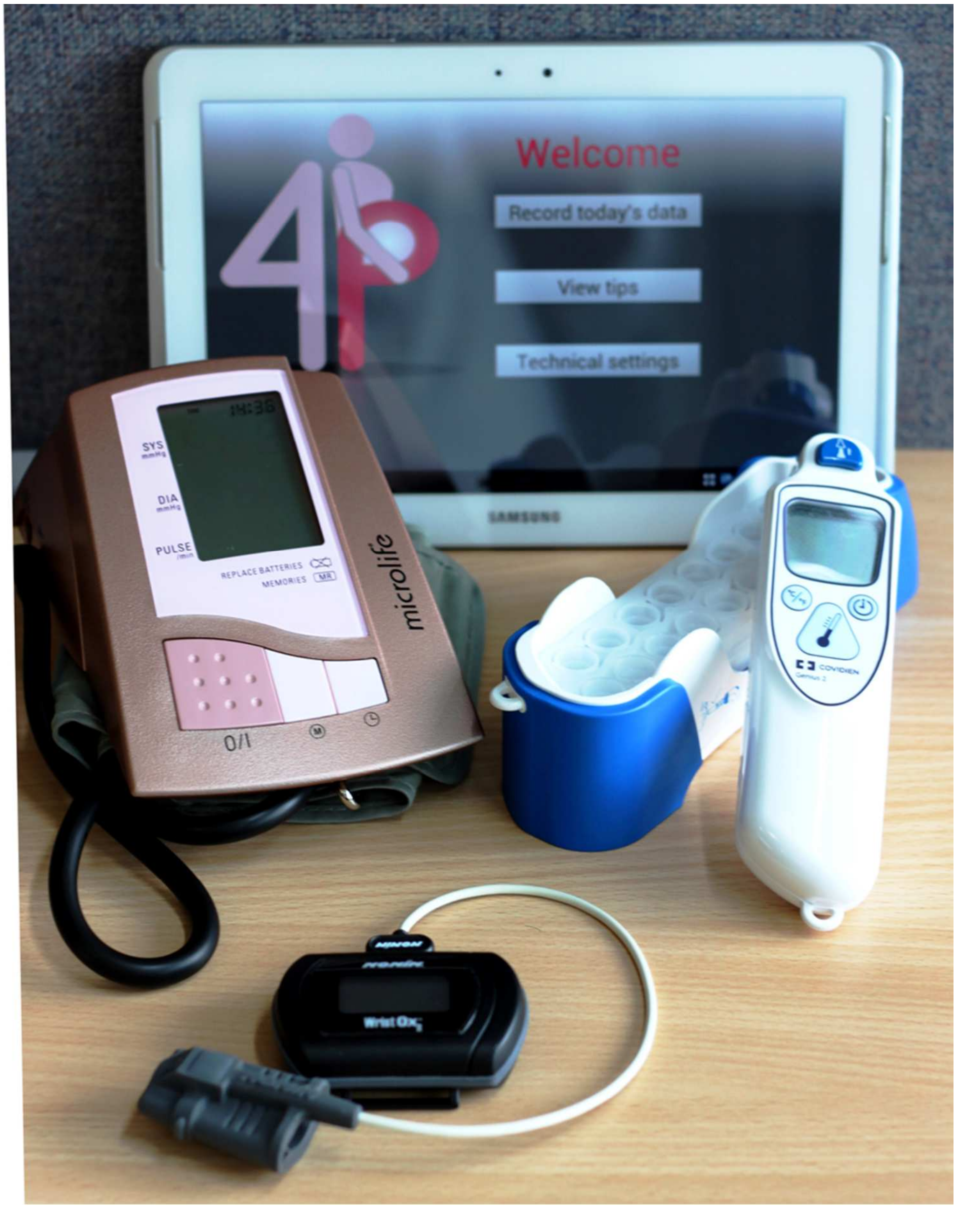

Equipment used for vital sign data collection

92x117mm (300 x 300 DPI)

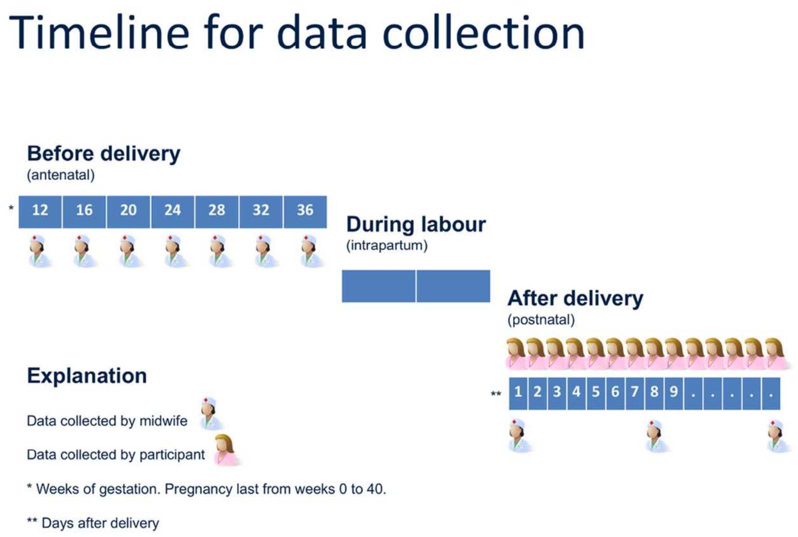

Timeline of follow up visits during the antenatal and postnatal periods.

121x85mm (300 x 300 DPI)

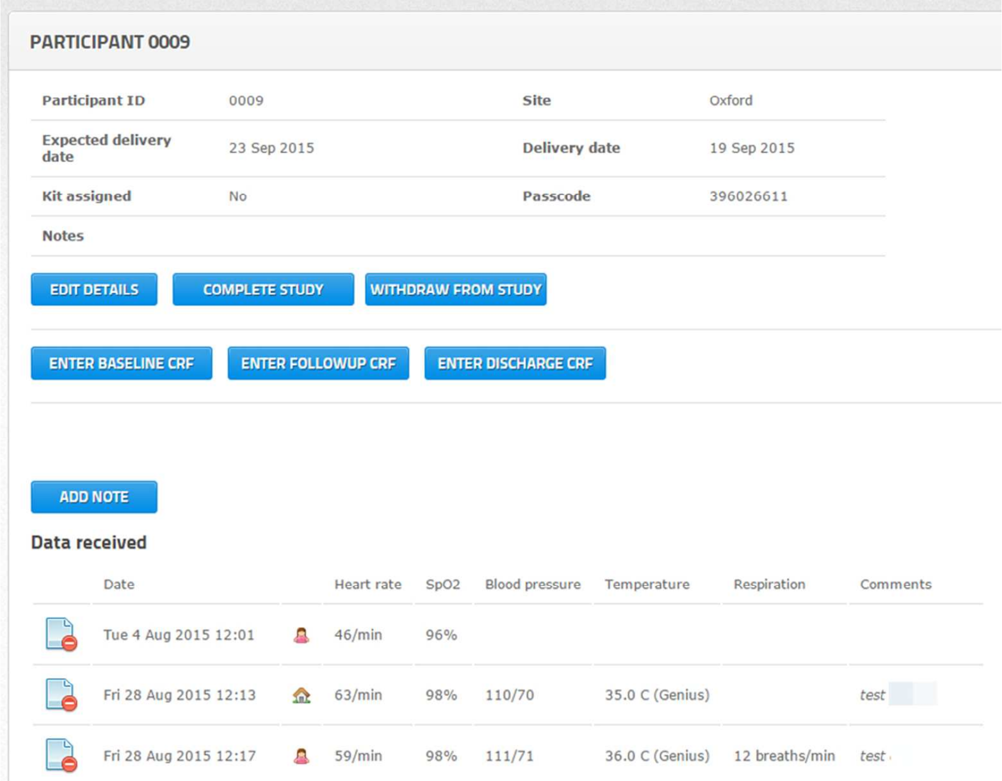

Review of vital sign data for participant 0009 on the 4P website (example).

87x67mm (300 x 300 DPI)

APPENDICES

Appendix 1: Sites in study

John Radcliffe Hospital, Oxford University Hospitals NHS Foundation Trust, UK

Royal Victoria Infirmary, The Newcastle Upon Tyne Hospitals NHS Foundation Trust, UK

St Thomas' Hospital, Guy's and St Thomas' NHS Foundation Trust, UK

Appendix 2: Inclusion/Exclusion criteria

Inclusion Criteria

a) ≥16 years of age (parental consent to be obtained for participants <18 years old)

b) BMI < 35 kg/m2

c) Ultrasound dating scan < 14 weeks

d) Naturally conceived pregnancy

e) Singleton pregnancy

f) Pregnant with an intent to deliver in hospital

g) Able to understand written or spoken English or has their own interpreter willing to translate

h) Willing and able to use the home monitoring equipment

i) Within category 1 of the American Society of Anaesthesiologists' classification of physical status at enrolment ("A normal healthy patient without any clinically important co-morbidity and without a clinically significant past/present medical history"). Women with the following conditions would be included in the 4P study, under the above definition:

- Obesity
- Smoking
- Diabetes
- Renal disease without hypertension
- High BP at recruitment (defined as SBP > 140mmHg and DBP > 90mmHg, not previously diagnosed with hypertension)
- Gestational hypertension or pre-eclampsia diagnosed in a previous pregnancy (even if treated, as long as essential hypertension was not developed)

Exclusion Criteria

Any known medical condition expected by the recruiting clinician to alter maternal vital signs, such as:

- Hypertension (defined as SBP > 140mmHg and DBP > 90mmHg), ever requiring treatment
- Congenital cardiac disease
- Ischaemic cardiac disease

For peer review only

- Bronchiectasis
- Fibrotic lung disease
- Malignant disease

Appendix 3: Intrapartum case report form (electronic)

Intrapartum data is transcribed from patient notes through electronic case report forms. Screenshots of these intrapartum forms are presented in the following four figures.

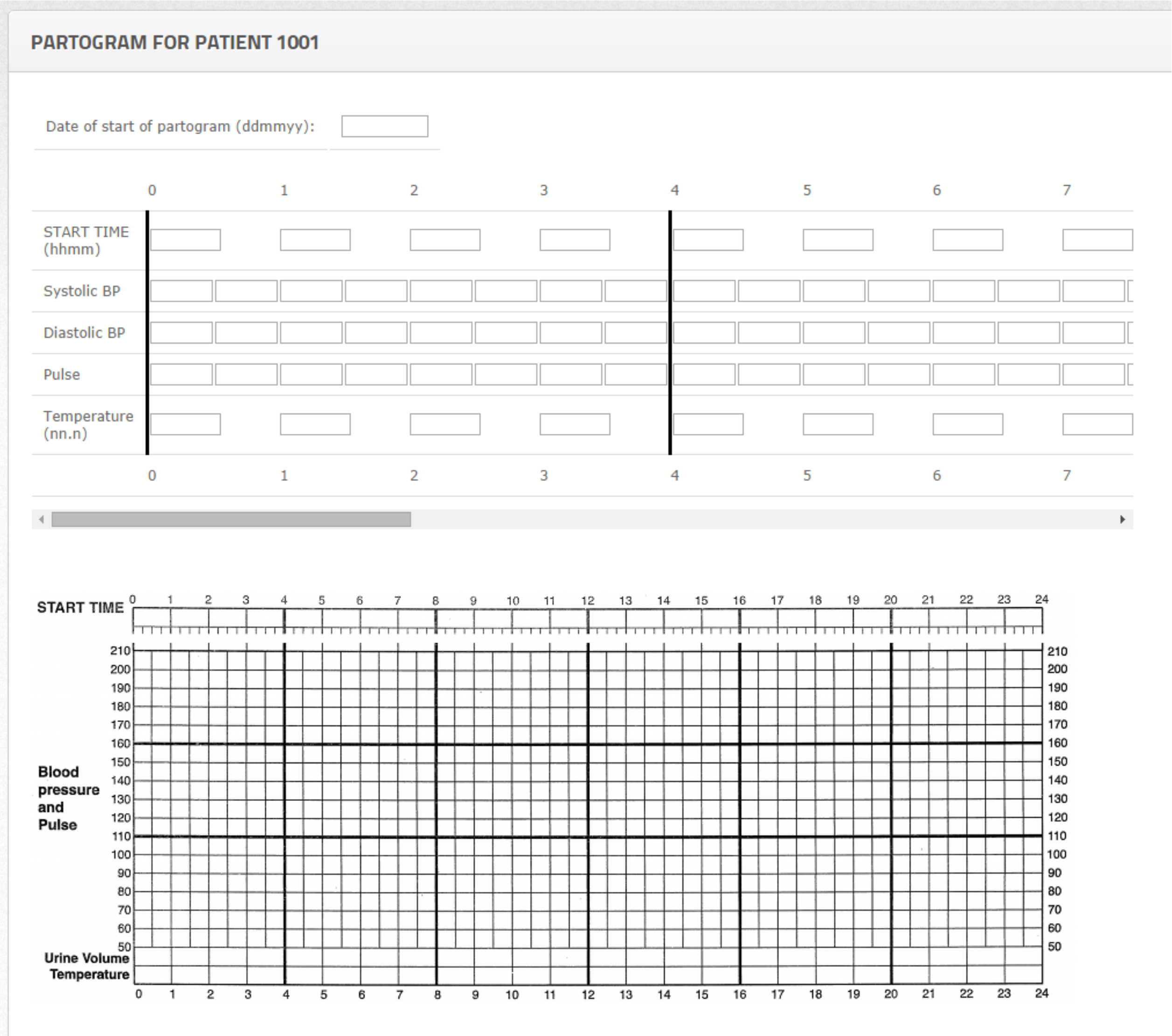

Partogram chart

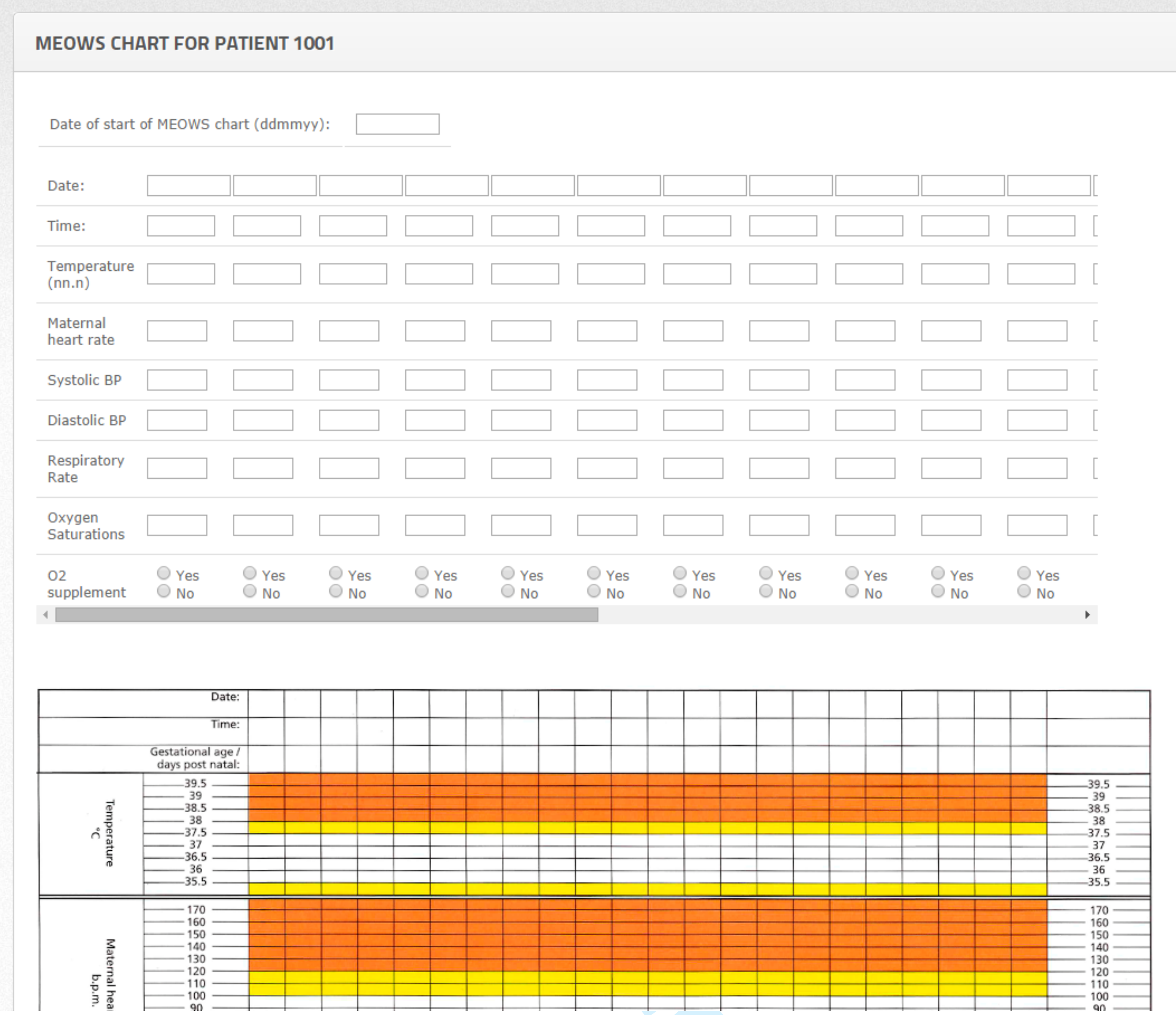

Maternal early warning score chart (referred to as a MEOWS chart in OUHs).

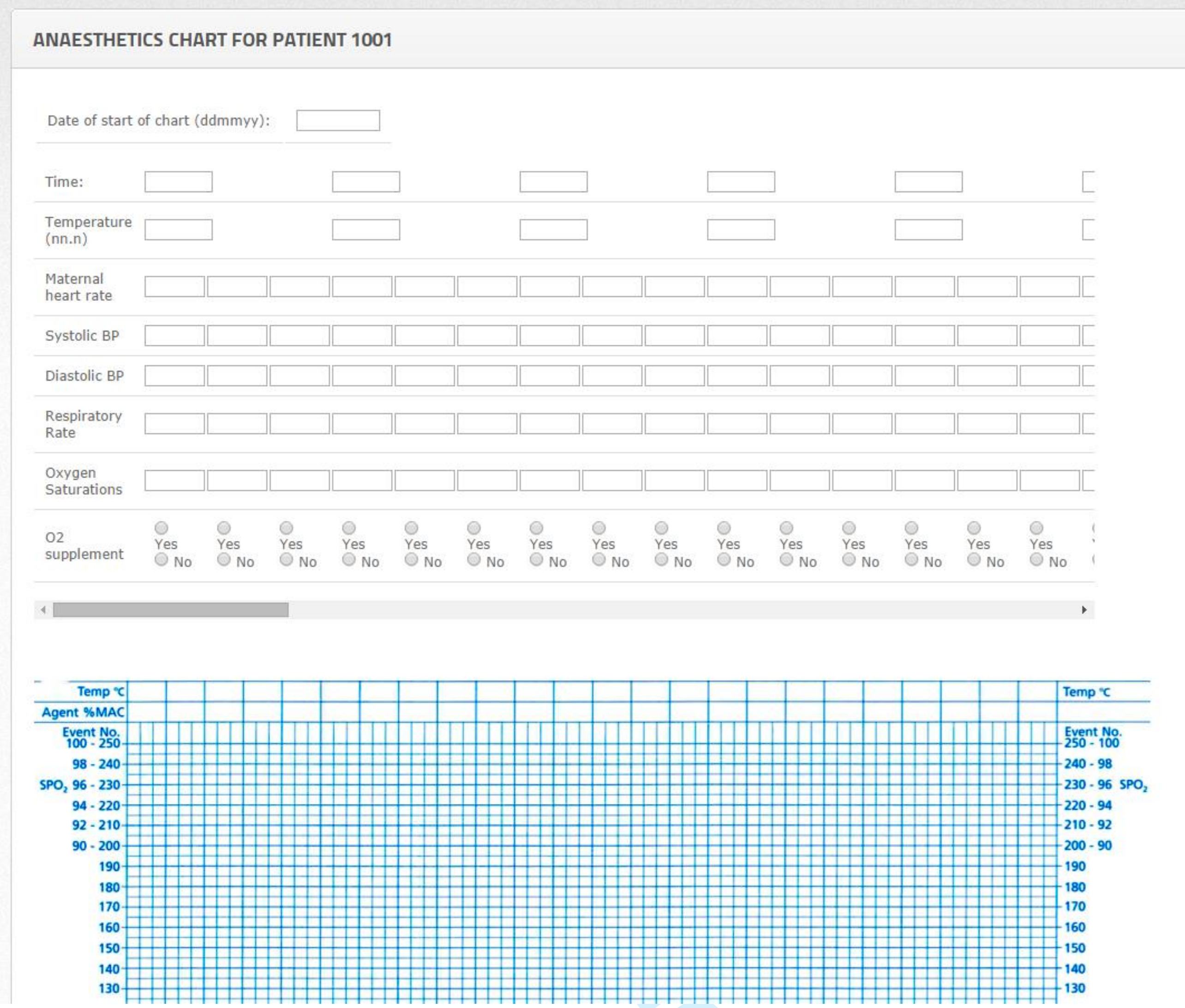

Anaesthetics chart

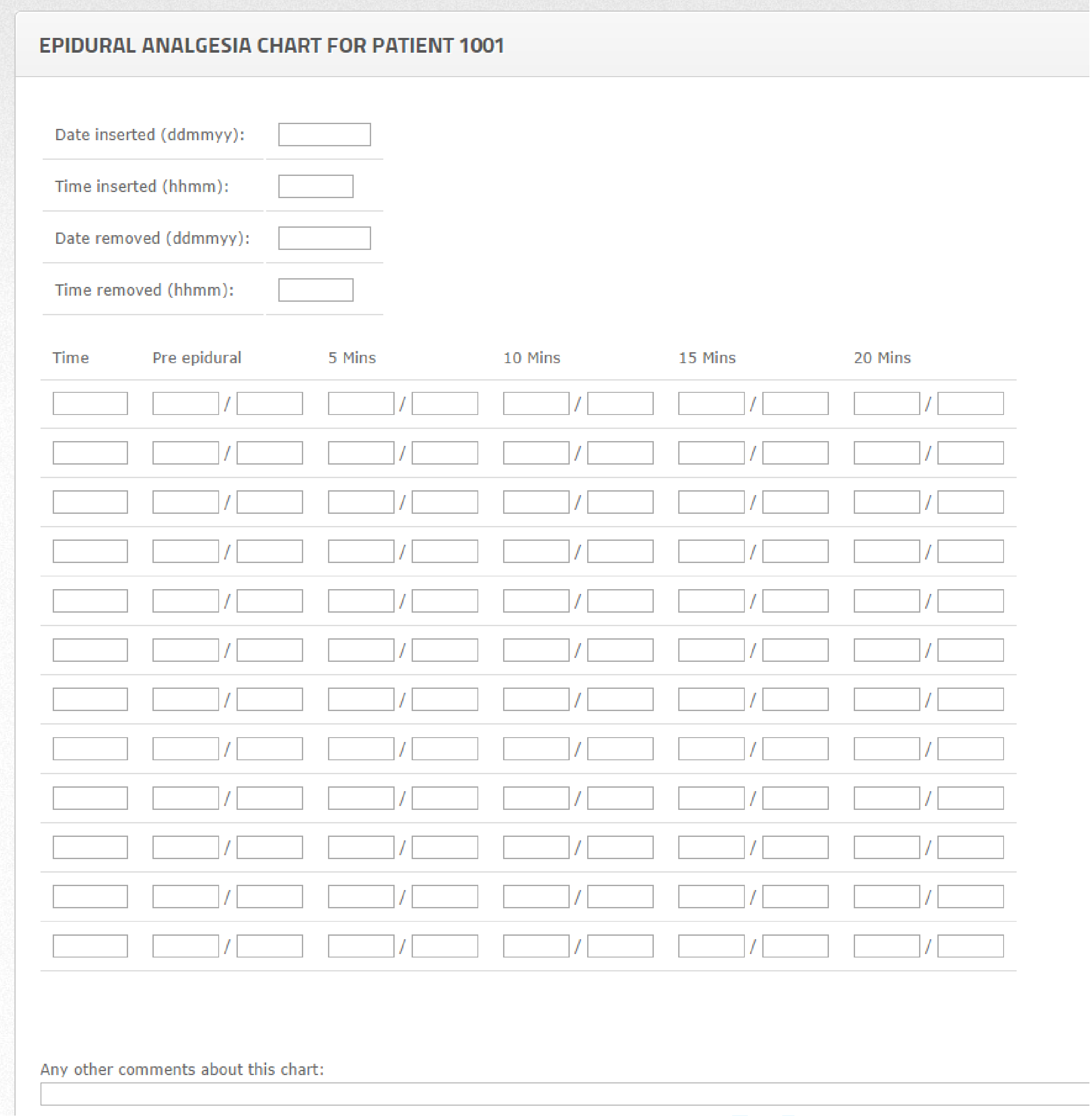

EPIDURAL ANALGESIA CHART FOR PATIENT 1001

Date inserted (ddmmyy):	
Time inserted (hhmm):	
Date removed (ddmmyy):	
Time removed (hhmm):	

Time	Pre epidural	5 Mins	10 Mins	15 Mins	20 Mins
	/	/	/	/	/
	/	/	/	/	/
	/	/	/	/	/
	/	/	/	/	/
	/	/	/	/	/
	/	/	/	/	/
	/	/	/	/	/
	/	/	/	/	/
	/	/	/	/	/
	/	/	/	/	/
	/	/	/	/	/
	/	/	/	/	/

Any other comments about this chart:

Epidural chart

Appendix 4: Case Report Forms

Supporting information are extracted from the participant's notes at each appointment. For example, the form used to collect demographic information, medical and obstetric history, current health status and pregnancy-related health and current medications at the initial baseline assessment, is shown below.

(1) BASELINE INFORMATION

Participant ID:	__ __ __ __
Date:	__ __ - __ __ - __ __ __ __ (D D M M Y Y Y Y)

SECTION 1: DEMOGRAPHIC INFORMATION

**1.1** Date of Birth	__ __ - __ __ - __ __ __ __	**1.7** Haemoglobin Level	__ __ . __ g/dl
**1.2** Height	__ __ __ . __ cm	**1.8** OR Haematocrit	__ __ . __ %
**1.3** Weight	__ __ __ . __ kg	**1.9** Date of blood test:	__ __ - __ __ - __ __ __ __
**1.4** In the last three months, has she smoked?	☐ Yes ☐ No	**1.10** Proteinuria (by dipstick)	☐ 0/trace ☐ + ☐ ++
**1.5** If yes, how many cigarettes/cigars per day?	__ __	☐ +++ ☐ ++++	☐ No urine test available
**1.6** Has she lived in the same household as someone who smokes?	☐ Yes ☐ No	**1.11** Date of urine test:	__ __ - __ __ - __ __ __ __

**1.12** Ethnicity	White	Asian or Asian British	Black or Black British
	☐ A : White - British	☐ H : Indian	☐ M : Black - Caribbean
	☐ B : White - Irish	☐ J : Pakistani	☐ N : Black - African
	☐ C : Any other White background	☐ K : Bangladeshi	☐ P : Any other Black background
	Mixed	☐ L : Any other Asian background	Other ethnic group
	☐ D : Mixed - White and Black Caribbean		☐ R : Chinese
	☐ E : Mixed - White and Black African		☐ S : Any other Ethnic Group
	☐ F : Mixed - White and Asian		☐ Z : Does not want to give Ethnic Group
	☐ G : Any other mixed background		

SECTION 2: MEDICAL HISTORY

Has she ever been diagnosed with or treated for any of the following medical conditions?

**2.1** Diabetes	☐ Yes ☐ No	**2.9** Hepatic disease	☐ Yes ☐ No
**2.2** Thyroid disease	☐ Yes ☐ No	**2.10** Gastrointestinal disease	☐ Yes ☐ No
**2.3** Other endocrinological condition	☐ Yes ☐ No	**2.11** Autoimmune conditions	☐ Yes ☐ No
**2.4** Any type of malignancy/cancer (including leukaemia or lymphoma)	☐ Yes ☐ No	**2.12** Haematological conditions	☐ Yes ☐ No
**2.5** Cardiac disease	☐ Yes ☐ No	**2.13** Venous thromboembolism (VTE)	☐ Yes ☐ No
**2.6** Hypertension without treatment	☐ Yes ☐ No	**2.14** Any congenital abnormality or genetic disease	☐ Yes ☐ No
**2.7** A chronic respiratory disease (including chronic asthma)	☐ Yes ☐ No	If yes, specify: ________________	
**2.8** Proteinuria, kidney disease or chronic renal disease	☐ Yes ☐ No	**2.15** Any other clinically relevant condition	☐ Yes ☐ No
		If yes, specify: ________________	

(Continued on next page)

For peer review only - http://bmjopen.bmj.com/site/about/guidelines.xhtml

SECTION 3: OBSTETRIC AND GYNAECOLOGICAL HISTORY

3.1 Date of first day of last menstrual period (LMP) ___ ___ - ___ ___ - ___ ___ ___ ___ (D D M M Y Y Y Y)

3.2 Number of previous pregnancies, excluding this pregnancy ___ ___

3.3 Number of previous births ___ ___

SECTION 4: CURRENT PREGNANCY-RELATED HEALTH

4.1 CRL (Crown Rump Length) measurement: ___ ___ ___ . ___ mm

4.2 Date of CRL measurement: ___ ___ - ___ ___ - ___ ___ ___ ___ (D D M M Y Y Y Y)

During this pregnancy, has she been diagnosed with or treated for any of the following conditions?

**4.3** Severe vomiting requiring hospitalisation	☐ Yes ☐ No	**4.5** Anaemia	☐ Yes ☐ No
**4.4** Hypertension	☐ Yes ☐ No	**4.6** Infection/febrile illness	☐ Yes ☐ No

SECTION 5: CURRENT MEDICATIONS

Is she routinely taking any of the following medication?

5.1 Aspirin ☐ Yes ☐ No

5.2 Non-steroidal anti-inflammatories ☐ Yes ☐ No

5.3 Insulin ☐ Yes ☐ No

5.4 Antihypertensives ☐ Yes ☐ No

Which antihypertensives?
- ☐ ACE inhibitors
- ☐ Beta-blockers
- ☐ Calcium channel blockers

5.5 Any other antibiotics/antivirals ☐ Yes ☐ No

If yes, specify: ____________________

5.6 Any other treatment ☐ Yes ☐ No

If yes, specify: ____________________

For peer review only - http://bmjopen.bmj.com/site/about/guidelines.xhtml

Appendix 5: Definitions

Definitions of variables collected in the 4P study, mostly taken from the National Institute for Health and Care Excellence (NICE) guidelines and quality standards.

Baseline information	
Ethnicity	Categories from the 2011 UK Census<br>A White - British<br>B White - Irish<br>C Any other White background<br>D Mixed - White and Black Caribbean<br>E Mixed - White and Black African<br>F Mixed - White and Asian<br>G Any other mixed background<br>H Indian<br>J Pakistani<br>K Bangladeshi<br>L Any other Asian background<br>M Black - Caribbean<br>N Black - African<br>P Any other Black background<br>R Chinese<br>S Any other Ethnic Group<br>Z Does not want to give Ethnic Group
Weight	Measured in kilograms (kg) to 1 decimal place.
Height	Measured in centimetres (cm) to 1 decimal place.
Crown Rump Length	Ultrasound measurement of crown rump length in millimetres (mm) to 1 decimal place.
Gestational age	Calculated from Crown Rump Length to the nearest day, using the algorithm developed by the Interbio study [1].
Smoker	Has smoked in the three months before recruitment.
Proteinuria	Presence of proteinuria in the urine, measured in milligrams/decilitre (mg/dl) to 1 decimal place.
**Medical Conditions**	
Diabetes	Glucose intolerance. Any type of diabetes is recorded.
Thyroid disease	A medical condition impairing the function of the thyroid. For example: hypo- or hyper-thyroidism, arathyroidism (PTH).

For peer review only

Other endocrinological condition	Diseases related to the endocrine glands of the body. For example: Addison's disease, adrenal gland disorders, hypophysitis
Malignancy/cancer	A malignant tumour, where cells proliferate out of control and inhibit the normal function of an organ system. Any type of cancer recorded, including leukaemia or lymphoma.
Cardiac disease	Disease of the heart or blood vessels. For example: arrhythmias, murmurs, valve diseases, atherosclerosis, atrial fibrillation, sarcoma, pericarditis, or cardiomyopathy.
Respiratory disease	Diseases of the airways and other parts of the lung. For example: asthma, chronic obstructive pulmonary disease (COPD), lung cancer, cystic fibrosis, sleep apnoea or occupational lung diseases.
Pyelonephritis or kidney disease	Disease of the kidneys. For example: chronic kidney disease, polycystic kidney disease, or kidney failure.
Hepatic disease	Disease of the liver. For example: hepatitis, haemochromatosis, or alcohol-related liver disease.
Gastrointestinal disease	Diseases involving the gastrointestinal tract (oesophagus, stomach, small intestine, large intestine and rectum), and the accessory organs of digestion (liver, gallbladder, and pancreas). For example: Crohn's disease, coeliac disease, ulcerative colitis or any severe malabsorption condition (requiring special diet)
Autoimmune conditions	The body's immune system attacks and destroys healthy body tissue by mistake. There are more than 80 types of autoimmune disorders. For example: Addison's disease, multiple sclerosis, rheumatoid arthritis.
Haematological conditions	Disorder of the blood. For example: anaemia, bleeding disorders such as haemophilia, blood clots, and blood cancers such as leukaemia, lymphoma, and myeloma.
Venous thromboembolism	A blood clot in a vein.
Infection	The invasion and multiplication of microorganisms such as bacteria, viruses, and parasites that are not normally present within the body.
Febrile illness	A nonspecific term for any illness of sudden onset accompanied by fever.
**Pregnancy complications**	
Gestational diabetes	Any degree of glucose intolerance with onset or first recognition during pregnancy.
Gestational hypertension	Blood pressure is ≥ 140/90mmHg after 20 weeks gestation in a previously normotensive pregnancy [2]
Preeclampsia	Gestational hypertension with the presence of proteinuria (excessive protein substance, chiefly albumin, in the urine)

For peer review only

Severe preeclampsia	Blood pressure is ≥ 160/110mmHg on two occasions, between 4 and 168 hours apart, or if the first measurement was immediately followed by treatment with an antihypertensive, either of these scenarios being associated with the presence of proteinuria.
Eclampsia	The occurrence of convulsions and/or coma unrelated to her cerebral conditions in a woman with signs and symptoms of pre-eclampsia. Seizures are of grand mal type and may first appear before labour, during labour, or up to 48 hours postpartum.
HELLP syndrome	A group of symptoms that occur in pregnant women who have pre-eclampsia or eclampsia and who also show signs of liver damage and abnormalities in blood clotting. It is characterised by: **H**aemolysis, **EL** (elevated) liver enzymes and **LP** (low platelet) count.
Primary postpartum haemorrhage	Blood loss from the genital tract >500mls (vaginal birth) or >1000mls (caesarean birth) within 24 hours of the birth of a baby. [3]
Secondary postpartum haemorrhage	Excessive or abnormal bleeding from the genital tract between 24 hours and 6 weeks in the postnatal period. [4]
Anaemia	Iron deficiency. Anaemia in pregnancy is defined as first trimester haemoglobin (Hb) less than 110 g/l, second/third trimester Hb less than 105 g/l, and postpartum Hb less than 100 g/l, in line with British Committee for Standards in Haematology (BCSH) guidance. [5]
**Delivery and labour**	
Spontaneous vaginal delivery	Spontaneous initiation of labour and delivery with no intervention.
Vaginal assisted	Delivery assisted with forceps or vacuum. Vacuum extraction uses a suction cup that is placed over the baby's head, which allows the physician to pull the child through the birth canal.
Caesarean section	An operative delivery of baby through a small insertion to the mother's abdomen. Including elective (planned, performed at or after 39 weeks), and emergency (unplanned) caesarean sections.
Assisted breech or breech extraction	The baby is born feet-first or buttocks first and requires assistance.
Preterm delivery	Delivery before 37 weeks.
Preterm pre-labour rupture of membranes (PPROM)	Spontaneous rupture of the membranes at less than 37 weeks gestation and prior to onset of labour.
Foetal distress	Abnormal foetal heart rate (FHR) or biophysical profile (BPP) (as a reason for labour being induced or caesarean section performed)

For peer review only

References for Appendix 5:

1. Papageorghiou AT, Kennedy SH, Salomon LJ, Ohuma EO, Chiekh Ismail L, Barros FC, Lambert A, Carvalho, M, Jaffer YA, Bertino E, Gravett MG, Altman DG, Purwar M, Noble JA, Pang R, Victoria CG, Bhutta ZA, Villar J. International standards for early fetal size and pregnancy dating based on ultrasound measurement of crown-rump length in the first trimester of pregnancy. *Ultrasound in Obstetrics and Gynecology* Published Online First: 2 November 2014. doi: 10.1002/uog.13448.
2. National Institute for Health and Care Excellence (NICE). Hypertension in pregnancy: diagnosis and management (Clinical guideline 107). 2010. Retrieved 01-08-2016 from: nice.org.uk/guidance/cg107
3. Mousa HA, Alfirevic Z. Treatment for primary postpartum haemorrhage. *Cochrane Database of Systematic Reviews* Published First Online 13 February 2014. doi: 10.1002/14651858.CD003249.pub3
4. Alexander J,Thomas PW, Sanghera J. Treatments for secondary postpartum haemorrhage. *Cochrane Database of Systematic Reviews* Published Online First: 21 January 2002. doi: 10.1002/14651858.CD002867
5. Royal College of Obstetricians and Gynaecologists (RCOG). Blood transfusion in obstetrics (Green-top guideline 47). 2015. Retrieved 01-08-2016 from: https://www.rcog.org.uk/globalassets/documents/guidelines/gtg-47.pdf

For peer review only

Appendix 6: Usability survey

Oxford University Hospitals NHS
NHS Trust

University of Oxford

The 4P Participants' Usability Survey

Please answer each question 1-10 by placing a cross (X) on the scale to indicate your experience of using the 4P m-Health system.	*Strongly disagree 0*	*1*	*2*	*3*	*Strongly agree 4*
1. I think I would like to use the 4P system frequently	☐	☐	☐	☐	☐
2. I find the 4P system unnecessarily complex	☐	☐	☐	☐	☐
3. I think the 4P system is easy to use	☐	☐	☐	☐	☐
4. I think that I would need the ongoing support of a technical person or research midwife to be able to use the 4P system	☐	☐	☐	☐	☐
5. I find that the various functions within the 4P system are well integrated	☐	☐	☐	☐	☐
6. I think that there is too much inconsistency in the design of the 4P system	☐	☐	☐	☐	☐
7. I would imagine that most people would learn to use the 4P system very quickly	☐	☐	☐	☐	☐
8. I find the 4P system very cumbersome to use	☐	☐	☐	☐	☐
9. I feel very confident using the 4P system	☐	☐	☐	☐	☐
10. I needed to learn a lot of things before I could competently use the 4P system	☐	☐	☐	☐	☐
Additional feedback or comments (optional):					

Thank you for taking the time to complete this questionnaire

Appendix 7: Standard Operating Procedures (SOPs)

Three examples of SOPs used in the 4P study are presented in the following pages

- 001 How participants are taught to use the correct use of all the equipment
- 016 Extraction of patient notes onto the database
- 017 Measurement of each vital sign by midwives

For peer review only

STANDARD OPERATING PROCEDURE

Nuffield Department of Obstetrics and Gynaecology

SOP No: 001-V1

SOP Title: Teaching Kit Use To 4P Participants

**SOP Number**	**001-V1**
**SOP Title**	**Teaching Kit Use To 4P Participants**

	NAME	TITLE	SIGNATURE	DATE
**Author**	**Jude Kemp**	**Research Midwife**		**27/02/2013**
**Reviewer**				
**Authoriser**				

**Effective Date:**	
**Review Date:**	

READ BY

NAME	TITLE	SIGNATURE	DATE

Adapted from CTRG Template SOP Version 2.1
© Copyright: The University of Oxford 2009

For peer review only - http://bmjopen.bmj.com/site/about/guidelines.xhtml

1. PURPOSE

The purpose of this SOP is to describe a standardised method of teaching the use of the 4P home monitoring equipment.

Training all participants to the same standard will help eliminate user error as much as possible and ensure a high quality data set.

2. INTRODUCTION

The 4P study aims to capture 14 days' worth of physiological readings from participating women using a standardised home monitoring equipment kit. The readings consist of resting pulse oximetry, resting blood pressure, pulse and temperature.

The readings are collected onto a tablet computer by the participant and transmitted to the study database (hosted on a secure NHS server) at the end of each recording session. At the end of the 14 days, any un-submitted data are retrieved by 4P engineers and added to the study database for analysis. The data will be used to devise an obstetric early warning system.

It is important to ensure that the 4P participants are confident in using their home monitoring equipment before the start of the home monitoring phase.

The ideal teaching opportunity is at the participant's final two INTERBIO visits at approximately 36 and 38 weeks gestation.

If this is not possible, or the participant delivers prior to these appointments, the teaching should take place prior to discharge from hospital.

3. SCOPE

This SOP applies only to the 4P research midwives teaching the use of home monitoring equipment.

4. DEFINITIONS

The 4P home monitoring equipment consists of the following items:

- Tablet computer (Samsung Galaxy Tab) with charger and charging lead.
- Pulse oximeter (Nonin 3150 WristOx) with finger sensor (To ease understanding, it will be referred to as a finger meter)
- Thermometer (Genius 2) with a supply of lens caps (36 lens caps per unit)
- Blood pressure monitor (Microlife 3BTO-A(2)) with appropriate size cuff.
- 4P study instruction manual for parents describing set up of equipment.

Adapted from CTRG Template SOP Version 2.1
© Copyright: The University of Oxford 2009

For peer review only - http://bmjopen.bmj.com/site/about/guidelines.xhtml

5. RESPONSIBILITIES

The 4P midwife is responsible for teaching the correct use of the home monitoring equipment and ensuring the participant feels confident in its use.

6. SPECIFIC PROCEDURE

6.1 Before teaching the use of the home monitoring equipment, the 4P midwife should confirm that the 4P participant is still willing to take part in the 4P study. Where possible, arrange for the participant's partner or relative to be present and teach him/her the use of the equipment too.

6.2 Teaching should take place in a private room if possible to provide a calm environment for learning and asking questions. If a private room is not available (e.g. on a ward) the curtains should be drawn around the bed to provide privacy.

6.3 The 4 P midwife must explain the rationale behind the data collection and give the participant an opportunity to ask questions.

6.4 The 4P midwife will teach the use of the following:

a. Pulse oximeter

b. blood pressure cuff positioning and measurement

c. thermometer use

To ensure the participant has rested prior to the blood pressure reading, it is important that the recordings are always carried out in the following order:

Pulse Oximetry, Blood Pressure, Temperature

Adapted from CTRG Template SOP Version 2.1
© Copyright: The University of Oxford 2009

For peer review only - http://bmjopen.bmj.com/site/about/guidelines.xhtml

a. Demonstration of correct pulse oximeter use:

Tell the participant:

It is important that the following advice is always followed in order to obtain a correct reading.

Ask the participant to

- Switch on the Nonin Pulse oximeter and place the grey Nonin pulse oximeter finger meter over the index finger of the left hand with the cable side upper most.
- Place the oximeter on a flat surface and sit comfortably while it is recording.
- Refrain from speaking during the reading.

The pulse oximeter will not work if the participant is wearing false nails or dark nail varnish. Ask the participant to remove nail varnish. If she is wearing or intends to wear false nails, an ear clip will be supplied.

b. Demonstration of correct blood pressure cuff positioning

The appropriate cuff size should be used:

Standard cuff bladder 12-26 cm for the majority of adult arms (arm circumference 33-50 cm)

Large cuff bladder 12-40 cm for obese arms (arm circumference > 50 cm)

Small cuff bladder 12-18 cm for lean adult arms (arm circumference 23-33 cm)

(Combined European Society/Nice/British Hypertension Society recommendations, 2012) http://www.bhsoc.org/frequently-asked-questions/#BPM4

Adapted from CTRG Template SOP Version 2.1
© Copyright: The University of Oxford 2009

For peer review only - http://bmjopen.bmj.com/site/about/guidelines.xhtml

Tell the participant:

It is important that the following advice is always followed in order to obtain a correct reading.

Ask the participant to

- Sit with her arm stretched out and supported, for example on the arm of a chair or a cushion.
- The feet should be flat on the floor.
- Sit quietly and relax.
- Wear clothing with a loose sleeve as she will need to push it up. If the sleeve is tight, ask the participant to slip her arm out of the sleeve.
- Do not push tight sleeves up, as she will not be able to position the cuff correctly.
- Slip her arm into the cuff with the black tubing at the front and move it up the arm, 3cm above the elbow.
- Fasten the Velcro and adjust the position of the cuff if needed.
- Press the 'on' button. Explain that the cuff will go tight, which may be slightly uncomfortable.
- Encourage the participant not to talk during the measurement.
- Enter the BP value on the tablet computer.

IMPORTANT

- Explain that a high BP reading will cause a message to appear onscreen, asking her to repeat the measurement in half an hour.
- If the measurement is still high, a message will appear, asking her to access medical help via the Maternity Assessment Unit or Day Assessment Unit at the John Radcliffe Hospital or the Horton Hospital in Banbury. (see SOP 005 for details)
- Explain to the participant that it is her responsibility to access medical assistance and that the data she is recording are NOT monitored by a doctor or midwife.

Adapted from CTRG Template SOP Version 2.1
© Copyright: The University of Oxford 2009

For peer review only - http://bmjopen.bmj.com/site/about/guidelines.xhtml

c. Demonstration of correct use of the thermometer

Tell the participant:

It is very important that the following advice is always followed in order to obtain a correct temperature reading:

Ask the participant to:

- Lift the thermometer out of its cradle. See photo.

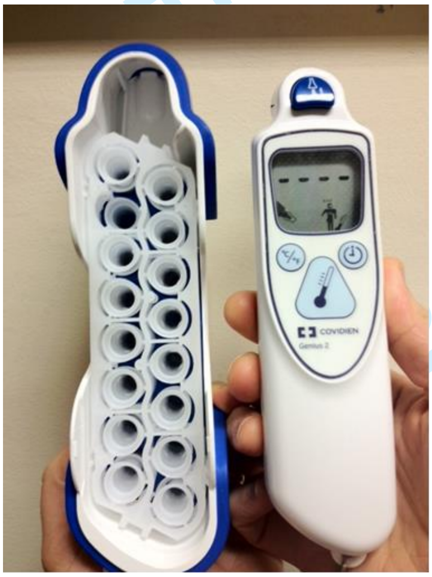

- Push the thermometer tip into a new lens cover from the pack, so that the lens cover clips onto the thermometer.
- Place the tip gently into the ear canal. See photo.

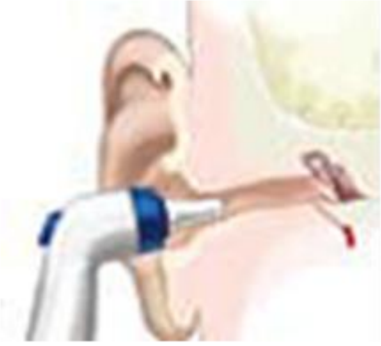

- Press the triangular button on the back of the thermometer.
- Wait for the triple beep before removing from the ear.
- Enter the number into the box on the tablet where indicated.

Adapted from CTRG Template SOP Version 2.1
© Copyright: The University of Oxford 2009

For peer review only - http://bmjopen.bmj.com/site/about/guidelines.xhtml

For peer review only

7. EXTERNAL REFERENCES

http://www.bhsoc.org/frequently-asked-questions/#BPM4

8. CHANGE HISTORY

SOP No 001, version 1

Effective Date: see page 1

Initial Version

Previous version: N/A

SOP no.	Effective Date	Significant Changes	Previous SOP no.

For peer review only

© Copyright: The University of Oxford 2009

For peer review only - http://bmjopen.bmj.com/site/about/guidelines.xhtml

STANDARD OPERATING PROCEDURE

Nuffield Department of Obstetrics and Gynaecology

SOP No: 016-V1

SOP Title: Data Entry

**SOP Number**	**016-V1**
**SOP Title**	**Data Entry**

	NAME	TITLE	SIGNATURE	DATE
**Author**	Jude Kemp<br>Fiona Kumar	Data Entry		
**Reviewer**				
**Authoriser**				

**Effective Date:**	
**Review Date:**	

READ BY

NAME	TITLE	SIGNATURE	DATE

Adapted from CTRG Template SOP Version 2.1
© Copyright: The University of Oxford 2009

For peer review only - http://bmjopen.bmj.com/site/about/guidelines.xhtml

STANDARD OPERATING PROCEDURE

Nuffield Department of Obstetrics and Gynaecology

SOP No: 016-V1

SOP Title: Data Entry

1. PURPOSE

The purpose of this SOP is to create clear guidelines on entering data from maternal anaesthetic charts, the partogram and MEOWS charts onto the 4P data base. This will help reduce errors in data entry and ensure high quality data is collected.

2. INTRODUCTION

The 4P study is collecting vital sign (blood pressure, pulse, oxygen saturations, temperature, and respiratory rate) readings from 1000 women throughout pregnancy, labour and the immediate post-partum period. Antenatally and postnatally the data are submitted to the database via a tablet computer.

The data from labour and delivery are manually entered by the research midwives onto the 4P study data base, copying each value form the handwritten charts used for vital sign recording.

When entering data manually from paper records onto an electronic database, there is scope for error by the person transcribing the data.

Some common causes of errors when transcribing data from paper charts onto electronic databases have been identified by Clifton and Watkinson (2015).

These are:

- The chart had illegible handwriting
- The incorrect row in the chart was transcribed
- The incorrect column in the chart was transcribed
- Vital signs were mismatched on the chart
- Typographical errors
- Errors of some other kind

Illegible handwriting was the most common cause of errors, with errors interpreting the grid like structure of the chart being second and third largest.

Whilst it is not possible to eliminate all errors occurring during the transcription of data, this SOP aims to minimise errors by describing in detail the methods used by the research midwives, with information on how to minimise the most commonly occurring errors described above.

Adapted from CTRG Template SOP Version 2.1
© Copyright: The University of Oxford 2009

For peer review only - http://bmjopen.bmj.com/site/about/guidelines.xhtml

STANDARD OPERATING PROCEDURE

Nuffield Department of Obstetrics and Gynaecology

SOP No: 016-V1

SOP Title: Data Entry

3. SCOPE

This SOP applies to all 4P Study midwives and research staff who will be entering data from charts used in labour and delivery onto the 4P database.

4. DEFINITIONS

Anaesthetic chart: A paper chart used by anaesthetists to document all maternal vital signs throughout surgical procedures

Partogram: A paper chart used by midwives to document the progress of labour, including timings of each stage of labour, rupture of amniotic membranes, cervical dilatation, fetal condition and maternal vital signs.

MEOWS (Modified Obstetric Early Warning System) chart: A paper chart used by midwives and doctors to document maternal vital signs during the in-hospital stay. The vital signs are assigned a score, action on these is decided according to this score.

5. RESPONSIBILITIES

It is the responsibility of each research midwife who will be entering data onto the 4P database to familiarise themselves with this SOP and follow the guidance in order to minimise. The SOP must be signed once it has been read.

6. SPECIFIC PROCEDURE

6.1 Entering an Anaesthetic chart:

On the anaesthetic chart, time is represented by boxes and columns. Each box represents five minutes, with each column representing 15 minutes.

Most anaesthetists enter blood pressure and pulse values onto the chart every 5 minutes.

Blood pressure readings are represented by arrow heads, a downward facing one for the systolic value, an upward facing one for the diastolic value.

Heart rate is represented by a dot on the relevant value.

For peer review only

© Copyright: The University of Oxford 2009

For peer review only - http://bmjopen.bmj.com/site/about/guidelines.xhtml

STANDARD OPERATING PROCEDURE

When entering the readings from the chart, the starting time is identified (see photo)

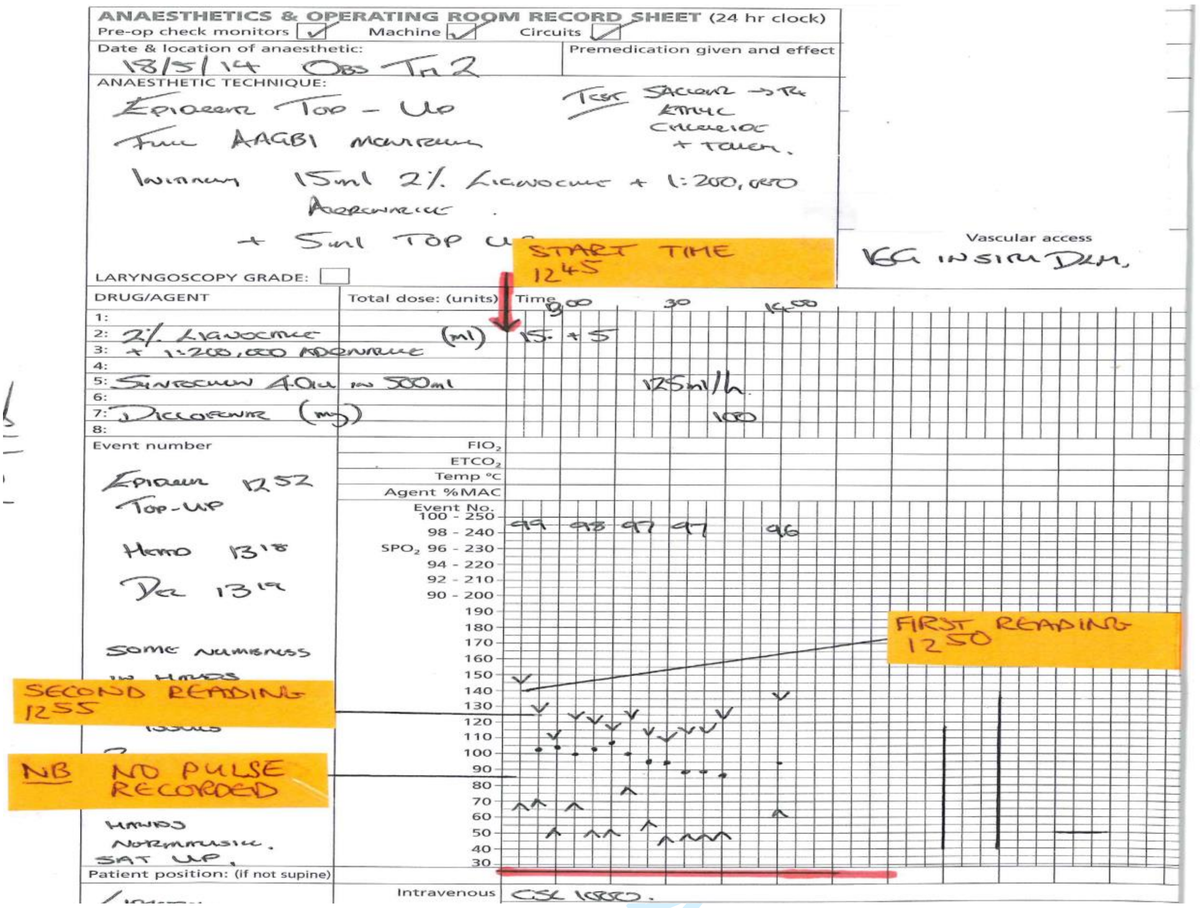

The start time of the chart may not be the time of the first reading to be entered, so it is important to ensure that the value is entered at the correct time, leaving a blank space if necessary.

On the above example, the start time is 12.45, but the first entry by the anaesthetist is 12.50. Readings are then entered every 5 minutes until 13.45, with one last one taken at 14.00.

© Copyright: The University of Oxford 2009

For peer review only - http://bmjopen.bmj.com/site/about/guidelines.xhtml

STANDARD OPERATING PROCEDURE

Nuffield Department of Obstetrics and Gynaecology

SOP No: 016-V1

SOP Title: Data Entry

The chart entry webpage through which the anaesthetic chart is entered (see image below) asks for the date and time to be entered first.

Next it asks for maternal heart rates. These are entered into each box at the corresponding time as seen on the chart above.

Note how the first column is left blank, as there is no reading for 12.45 and the first reading is entered at 12.50.

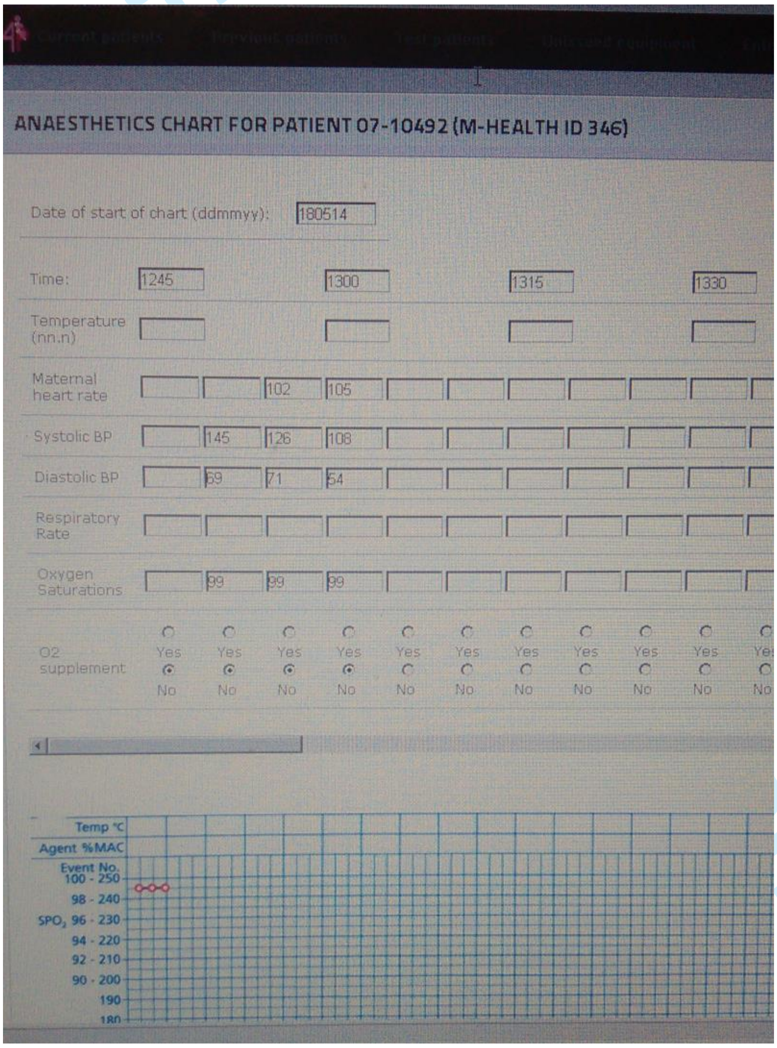

Adapted from CTRG Template SOP Version 2.1
© Copyright: The University of Oxford 2009

For peer review only - http://bmjopen.bmj.com/site/about/guidelines.xhtml

In order to avoid errors, it is easiest to enter all maternal pulses in sequence and then to move on to all systolic blood pressure values, before finishing off with the diastolic blood pressures.

The web page creates a visual representation, which aids with spotting errors as the data are entered (see picture below)

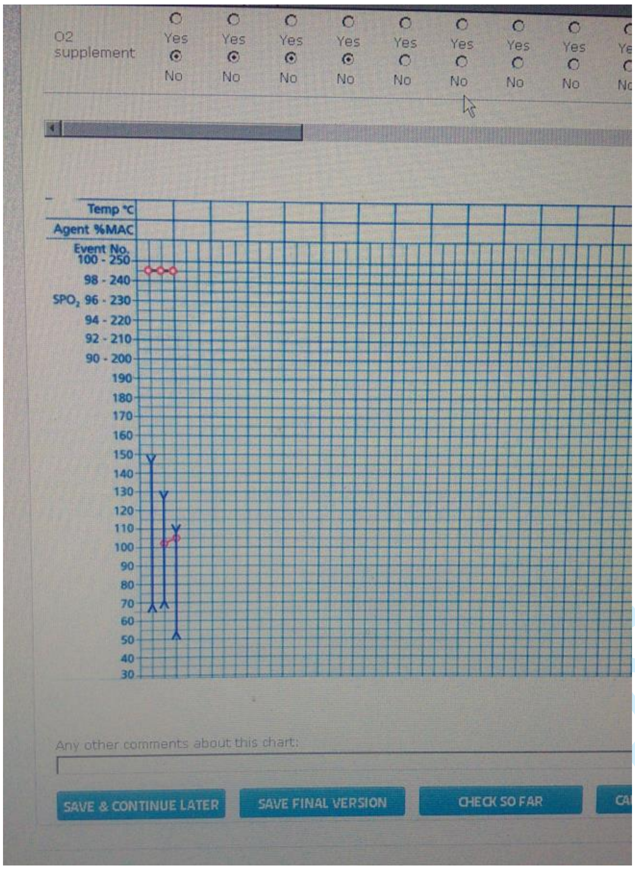

COMMON ERRORS. WRONG COLUMN:

On the above example, there is no pulse recorded at 12.50, so it is important to notice this and not enter the 12.55 pulse into the 12.50 box by mistake.

This section is the main text of the SOP. It details the procedure for the task to be performed.

There should be sufficient detail, clearly expressed, to enable a trained person to perform the procedure without supervision.

There should also be sufficient detail to enable a trained person to use the document to train others to perform the task.

The use of flow diagrams may be useful, especially in complex procedures.

© Copyright: The University of Oxford 2009

For peer review only - http://bmjopen.bmj.com/site/about/guidelines.xhtml

7. FORMS/TEMPLATES TO BE USED

Where Forms/Templates are referenced in the text, the numbers and titles are listed under this section.

8. INTERNAL AND EXTERNAL REFERENCES

This section is used to list all controlled internal references (e.g. SOPs) and external references referred to within the text of the SOP only.

8.1 Internal References

Insert relevant references as required, sufficient for the user to find the source document.

8.2 External References

Insert relevant references as required, sufficient for the user to find the source document. Web references should be included were possible.

9. CHANGE HISTORY

Where the SOP is the initial version:

- SOP No: Record the SOP and version number
- Effective Date: Record effective date of the SOP or "see page 1"
- Significant Changes: State, "Initial version" or "new SOP"
- Previous SOP no.: State "NA".

Where replacing a previous SOP:

- SOP No: Record the SOP and new version number
- Effective Date: Record effective date of the SOP or "see page 1"
- Significant Changes: Record the main changes from previous SOP
- Previous SOP no.: Record SOP and previous version number

SOP no.	Effective Date	Significant Changes	Previous SOP no.

Adapted from CTRG Template SOP Version 2.1
© Copyright: The University of Oxford 2009

For peer review only - http://bmjopen.bmj.com/site/about/guidelines.xhtml

STANDARD OPERATING PROCEDURE

Nuffield Department of Obstetrics and Gynaecology

SOP No: 017-V1

SOP Title: Home Visit Data Collection by 4P Midwife

**SOP Number**	**017-V1**
**SOP Title**	**Home Data Collection by 4P Midwife**

	NAME	TITLE	SIGNATURE	DATE
**Author**	**Jude Kemp Fiona Kumar**	**Research Midwife**		**15 April 2015**
**Reviewer**				
**Authoriser**				

**Effective Date:**	
**Review Date:**	

READ BY

NAME	TITLE	SIGNATURE	DATE

Adapted from CTRG Template SOP Version 2.1
© Copyright: The University of Oxford 2009

For peer review only - http://bmjopen.bmj.com/site/about/guidelines.xhtml

STANDARD OPERATING PROCEDURE

Nuffield Department of Obstetrics and Gynaecology

SOP No: 017-V1

SOP Title: Home Visit Data Collection by 4P Midwife

1. PURPOSE

The purpose of this SOP is to ensure that the 4P midwives are carrying out home visits to 4P participants in a standardised way. This is to ensure that a full set of high quality data is collected.

2. INTRODUCTION

Research Midwives will be recording medical and obstetric history as well as measuring participant's heart rate, blood pressure, oxygen saturation, respiratory rate and temperature at the booking visit, follow-up visits and one week and two weeks post-delivery.

3. SCOPE

This SOP applies to the 4P research midwives visiting the 4P participants to obtain a full data set.

4. DEFINITIONS

Full data set taken by 4P midwife:

- Medical and obstetric history
- Blood pressure
- Pulse
- Temperature using 4P midwives' 'gold standard' thermometer (Genius 2)
- Pulse oximetry
- Respiration count using accelerometer in smart phone and chest wall movement count

5. RESPONSIBILITIES

It is the responsibility of the 4P midwives to become familiar with the content of this SOP as they will be recording data during home visits.

Adapted from CTRG Template SOP Version 2.1
© Copyright: The University of Oxford 2009

For peer review only - http://bmjopen.bmj.com/site/about/guidelines.xhtml

6. SPECIFIC PROCEDURE

- Access the 4P website via 3/4G or wifi and open the electronic crf that corresponds with the participant's ID to complete the relevant medical and obstetric history sections
- Exit the application and open the android app for 4P data input and enter the participant's ID
- Connect the participant's finger to the pulse oximeter.
- Press the 'connect to finger probe' button on the app and ensure that the Nonin pulse oximeter is connected to the tablet.
- Explain to participant that her heart rate will be taken by palpating the radial pulse for 60 seconds.
- Palpate participant's radial pulse for 60 seconds and enter the measurement into the appropriate box on the app.
- Explain that the respiratory rate will be obtained using a smart phone placed on the participant's chest.
- Open the breathing rate app on the smart phone, enter the participant ID, attach the smart phone around the participant's neck and ask her to sit back in a chair so that the phone is resting on her chest.
- Ask participant to refrain from speaking during the measurement.
- Press the 'start counting breaths' button on the smartphone. A bleep will be heard.
- Wait for a second bleep. The word 'count' will appear on the screen. Press the 'count respirations' button on the tablet.
- For every breath, at the peak of inspiration, press the on-screen button on the tablet. The count takes 60 seconds.
- Count the participant's chest wall movements until a third bleep is heard from the smartphone and the word 'recording' appears on the screen.
- Press 'stop recording' on the screen and remove the smart phone from the participant's neck.
- Take the participant's temperature using the 4P Genius thermometer and enter it onto the tablet
- Take the participant's blood pressure;

Adapted from CTRG Template SOP Version 2.1
© Copyright: The University of Oxford 2009

For peer review only - http://bmjopen.bmj.com/site/about/guidelines.xhtml

The appropriate cuff size should be used:

Standard cuff bladder 12-26 cm for the majority of adult arms (arm circumference 33-50 cm)

Large cuff bladder 12-40 cm for obese arms (arm circumference > 50 cm)

Small cuff bladder 12-18 cm for lean adult arms (arm circumference 23-33 cm)

(Combined European Society/Nice/British Hypertension Society recommendations, 2012) http://www.bhsoc.org/frequently-asked-questions/#BPM4

Ask the participant to

- Sit with her arm stretched out and supported, for example on the arm of a chair or a cushion.
- The feet should be flat on the floor.
- Sit quietly and relax.
- Wear clothing with a loose sleeve as she will need to push it up. If the sleeve is tight, ask the participant to slip her arm out of the sleeve.
- Do not push tight sleeves up, as she will not be able to position the cuff correctly.
- Slip her arm into the cuff with the black tubing at the front and move it up the arm, 3cm above the elbow.
- Fasten the Velcro and adjust the position of the cuff if needed.
- Press the 'on' button. Explain that the cuff will go tight, which may be slightly uncomfortable.
- Encourage the participant not to talk during the measurement.
- Enter the BP value on the tablet computer.

IMPORTANT

Explain that a high BP reading will cause a message to appear onscreen, asking her to repeat the measurement.

If the measurement is still high, a message will appear, asking her to access medical help

Explain to the participant that it is her responsibility to access medical assistance and that the data she is recording are NOT monitored by a doctor or midwife.

© Copyright: The University of Oxford 2009

For peer review only - http://bmjopen.bmj.com/site/about/guidelines.xhtml

- Enter the respiration count onto the tablet.
- Explain to the participant that the measurements will be taken a second time and carry them out as above, beginning with the palpation of the radial pulse.

7. CHANGE HISTORY

Where the SOP is the initial version:

- SOP No: 0017
- Effective Date: see page 1
- Significant Changes: Initial version
- Previous SOP no.: NA

SOP no.	Effective Date	Significant Changes	Previous SOP no.

Adapted from CTRG Template SOP Version 2.1
© Copyright: The University of Oxford 2009

For peer review only - http://bmjopen.bmj.com/site/about/guidelines.xhtml

Study Title: The Pregnancy Physiology Pattern Prediction Study (4P study): an observational cohort study.

Internal Reference Number / Short title: 4P

Ethics Ref: 14/LO/1312

Date and Version No: 14 September 2015, Version 3

Chief Investigator: Peter Watkinson

Intensive Care Consultant

Kadoorie Centre for Critical Care Research and Education

John Radcliffe Hospital

Headley Way

Headington

Oxford, OX3 9DU

Investigators: Professor Lionel Tarassenko

Professorial Fellow in Electrical and Electronic Engineering

Institute of Biomedical Engineering

Old Road Campus Research Building

University of Oxford

OX3 7DQ

Oxford

Dr Lucy Mackillop

Consultant in Obstetric Medicine

Oxford University NHS Trust

Oxford

OX3 9DU

For peer review only

For peer review only - http://bmjopen.bmj.com/site/about/guidelines.xhtml
© Copyright: The University of Oxford and Oxford University Hospitals NHS Trust 2013

Professor Stephen Kennedy

Head of Department, Professor of Reproductive Medicine & Honorary Consultant

Nuffield Department of Obstetrics and Gynaecology

Oxford

OX3 9DU

Sponsor: University of Oxford

Funder: Oxford: Oxford Biomedical Research Centre

Investigator Signatures

Peter Watkinson

Lionel Tarassenko

Lucy Mackillop

Stephen Kennedy

No conflicts of interest declared by any investigator.

Confidentiality Statement

This document contains confidential information that must not be disclosed to anyone other than the Sponsor, the Investigator Team, host organisation, and members of the Research Ethics Committee, unless authorised to do so.

For peer review only

TABLE OF CONTENTS

1. SYNOPSIS 5
2. ABBREVIATIONS 6
3. BACKGROUND AND RATIONALE 6
4. OBJECTIVES AND OUTCOME MEASURES/ENDPOINTS 9
5. STUDY DESIGN 9
6. PARTICIPANT IDENTIFICATION 10
   6.1. Study Participants 10
   6.2. Inclusion Criteria 10
   6.3. Exclusion Criteria 10
7. STUDY PROCEDURES 10
   7.1. Screening and Recruitment 10
   7.2. Informed Consent 11
   7.3. Baseline Assessments .... 127.4.
   Subsequent Visits throughout pregnancy 12
   7.5. Discontinuation/Withdrawal of Participants from Study 16
   7.6. Definition of End of Study 16
8. STATISTICS AND ANALYSIS 16
   8.1. The Number of Participants 16
   8.2. Description of Statistical Methods 16
   8.3. Analysis of Outcome Measures/Endpoints 17
9. DATA MANAGEMENT 17
   9.1. Access to Data 17
   9.2. Data Recording and Record Keeping 17
10. QUALITY ASSURANCE PROCEDURES 20 1
11. ETHICAL AND REGULATORY CONSIDERATIONS 201
   11.1. Declaration of Helsinki 212
   11.2. Guidelines for Good Clinical Practice 212
   11.3. Approvals 212
   11.4. Reporting 212
   11.5. Participant Confidentiality 212
   11.6. Expenses and Benefits 22
   11.7. Other Ethical Considerations .... **Error! Bookmark not defined.**

For peer review only

For peer review only - http://bmjopen.bmj.com/site/about/guidelines.xhtml
© Copyright: The University of Oxford and Oxford University Hospitals NHS Trust 2013

12. FINANCE AND INSURANCE 223
12.1. Funding 223
12.2 Insurance 223
13. PUBLICATION POLICY 223
14. REFERENCES 234
15. APPENDIX A: STUDY FLOW CHART 245
16. APPENDIX B: SCHEDULE OF STUDY PROCEDURES 256
17. APPENDIX C: AMENDMENT HISTORY 267

For peer review only

For peer review only - http://bmjopen.bmj.com/site/about/guidelines.xhtml
© Copyright: The University of Oxford and Oxford University Hospitals NHS Trust 2013

1. SYNOPSIS

The 4P study is a multi-centre study which will collect standard physiological data and relevant associated information from pregnant women from their booking appointment through and including delivery and for the first two weeks post-partum. This data will be used to produce nomograms for the physiology of these women, from which a suggested National Maternal Obstetric Early Warning score will be produced.

**Study Title**	The Pregnancy Physiology Pattern Prediction Study (4P study)	
**Internal ref. no. / short title**	4P	
**Study Design**	Prospective Observational Cohort Study	
**Study Participants**	Pregnant women without significant cardiorespiratory comorbidities	
**Planned Sample Size**	1000	
**Planned Study Period**	2 years	
	**Objectives**	**Endpoints**
**Primary**	To develop a database of physiology of pregnancy and the postpartum period	Estimates of distributions and associated centiles for five vital signs throughout pregnancy and the postpartum period
**Secondary**	To develop a centile based early warning score for pregnancy and the post-partum period<br><br>To investigate new patterns within vital signs data in pregnancy	Centile-based thresholds for five vital signs throughout pregnancy<br><br>Trend analyses of five vital signs and inter-relations between these trends

For peer review only

For peer review only - http://bmjopen.bmj.com/site/about/guidelines.xhtml
© Copyright: The University of Oxford and Oxford University Hospitals NHS Trust 2013

2. ABBREVIATIONS

Define all unusual or 'technical' terms related to the project. Add or delete as appropriate to your study. Maintain alphabetical order for ease of reference.

4P Study	The Pregnancy Physiology Pattern Prediction
CI	Chief Investigator
CRF	Case Report Form
CTRG	Clinical Trials & Research Governance, University of Oxford
GCP	Good Clinical Practice
GP	General Practitioner
ICF	Informed Consent Form
ICH	International Conference of Harmonisation
MEOWS	Modified Obstetric Early Warning System
NHS	National Health Service
NRES	National Research Ethics Service
PI	Principal Investigator
PIL	Participant/ Patient Information Leaflet
R&D	NHS Trust R&D Department
REC	Research Ethics Committee
SOP	Standard Operating Procedure

3. BACKGROUND AND RATIONALE

Modified Early Obstetric Warning Charts (MEOWS) assign increasing scores (or colours) to each individual physiological measurement (blood pressure, heart rate, temperature, respiratory rate and oxygen saturation) as they become more abnormal. If either the sum of scores for several physiological measurements, or a score for a single physiological measurement exceed set thresholds, an alert is triggered. The two most recent Confidential Enquiries into Maternal Deaths in the UK have highlighted an urgent need to develop a national Modified Early Obstetric Warning System to aid the more timely recognition, treatment and referral of women who are developing life-threatening complications of pregnancy[1,2]. This recommendation was one of the ten key recommendations to emerge from the 2011 enquiry, and was strongly supported by a national survey of obstetric anaesthetists[3]. The enquiry

For peer review only

For peer review only - http://bmjopen.bmj.com/site/about/guidelines.xhtml
© Copyright: The University of Oxford and Oxford University Hospitals NHS Trust 2013

acknowledged that recognising physiological deterioration is complicated by the normal changes in maternal physiology that occur both during pregnancy and immediately after delivery.

Although several[1,3] local Modified Obstetric Early Warning Systems have been developed, they have all been developed heuristically. One published early warning score[4] used the association of vital signs after admission to intensive care with mortality following admission to inform the development of a heuristic MEOWS. None of these systems have taken an evidence-based approach to defining the alerting thresholds from knowledge of the expected distributions of each vital sign, nor do they take account of the physiological trends that are thought to occur during pregnancy.

An evidence-based approach to identifying when women have become physiologically abnormal for their stage of pregnancy is required, to achieve widespread acceptance of a national MEOWS chart[5]. To achieve this, large numbers of systolic and diastolic blood pressures, temperatures, respiratory rates, oxygen saturations and heart rate observations from women at each stage of "normal" pregnancy, intrapartum and the post-partum period are required. These will allow normal ranges and their associated centiles throughout pregnancy to be defined in the modern era. Recognising normality is the first step to understanding abnormality.

A systematic review of the availability of this data is underway. Large amounts of longitudinal data for blood pressure during pregnancy already exist (for example from the longitudinal AVON[6] and BOSHI[7] studies), although much of this is now more than a decade old. Heart rate data is often recorded in these papers, so the spread of heart rate data availability is similar. These data suggest that clear trends occur during pregnancy, (for example with changes of around 6mmHg in systolic blood pressure over the course of pregnancy[6,7]), which need to be taken into account to ensure appropriate identification of abnormality for the stage. However, even for blood pressure, the distribution of the outer centiles (which are those required to identify abnormality using our evidence based approach) and the variability of these centiles throughout pregnancy, the intrapartum and post-partum periods are not normally published. Little data exists for blood pressure during the intrapartum period and particularly for the first two weeks post-partum. There are also very little data for temperature, respiratory rate and oxygen saturations in pregnancy, the intrapartum or post-partum periods from which to define modern normal range centiles.

A centile-based approach to identifying abnormality will allow experts to choose when women should be reviewed, based on strong evidence of how far their observations lie from normality. The approach is particularly suited to areas like pregnancy, intrapartum and the post-partum period where the event rate is relatively low; "events" (or deteriorations) are poorly defined and interventions will occur in the study population as a result of abnormal physiology, preventing "events" from occurring. We have previously

For peer review only - http://bmjopen.bmj.com/site/about/guidelines.xhtml
© Copyright: The University of Oxford and Oxford University Hospitals NHS Trust 2013

used this approach both for the recognition of deterioration in hospitalised adults[8] and in the maintenance of safety in the aerospace industry[9].

As there is insufficient data to develop centile-based early warning scores for pregnancy, intrapartum or the post-partum period for any vital sign, a large database of these vital signs is urgently required to provide the data to underlie a national Obstetric Early Warning System. The availability and cost-effectiveness of modern technology now enables women to take measurements in the comfort of their own homes and longitudinal data taken from the same patients where possible with the same equipment across these periods is particularly important to ensure that step-changes between stages are not missed.

The proposed study follows on from identical work undertaken within the InterBio study in Oxford, which will form part of the final database. The proposed study will ensure that findings are replicable in other centres and that the database contains data from sufficient women to ensure accurate estimation of centiles in the distribution tails, where accuracy is important to allow recognition of abnormality.

For peer review only - http://bmjopen.bmj.com/site/about/guidelines.xhtml
© Copyright: The University of Oxford and Oxford University Hospitals NHS Trust 2013

4. OBJECTIVES AND OUTCOME MEASURES/ENDPOINTS

Objectives	Outcome Measures/Endpoints
**Primary Objective** To develop a database of physiology of pregnancy and the postpartum period	Database containing up to 1000 women's physiological variables
**Secondary Objective** To develop a centile based early warning score for pregnancy and the post-partum period	Centile-based distributions for each of systolic blood pressure, diastolic blood pressure, temperature, respiratory rate, heart rate and oxygen saturations

5. STUDY DESIGN

4P is a prospective observational cohort study involving 1000 participants across the UK with measurements taken in a home and hospital setting.

Participants will have their vital signs (blood pressure, heart rate, temperature, oxygen saturation levels and respiratory rate) measured by a research midwife at selected points throughout pregnancy .

After pregnancy they will be required to carry out home monitoring of their own vital signs using a set of home monitoring equipment for approximately 2 weeks.

The anticipated duration of participant involvement is from around 14 weeks pregnant to around 14 days postnatal (maximum 30 weeks to allow for women who deliver at 42 weeks).

Vital signs data will be collected from consented patients on a monthly basis from recruitment (at the first routine antenatal appointment or the nuchal scan) to two weeks post-partum. This will be a combination of data recorded at regular ante-natal visits, any in-hospital stay during pregnancy and immediately after birth, and data recorded by home monitors in the two weeks immediately after birth. Resting observations for the standard physiological variables used in the current MEOWS charts will be recorded from each mother. Demographic (including ethnicity), laboratory and medication data which may be related to vital signs will also be collected. As a result, a large database of maternal and post-partum physiology will be assembled, from which an evidence-based, national MEOWS will be developed for all stages of pregnancy, the intrapartum and post-partum periods. A multi-parameter, pregnancy-stage specific alerting system will be created for use in monitored areas and hospital environments.

For peer review only

For peer review only - http://bmjopen.bmj.com/site/about/guidelines.xhtml
© Copyright: The University of Oxford and Oxford University Hospitals NHS Trust 2013

Data for inclusion in the 4P database and subsequent analysis will also include participants from the Interbio study, who provide identical observations using the same methodologies as the 4P study, but within the Interbio study, which has its own ethical approval (08/H0606/139) and protocol. The dataflow from the Interbio study into the 4P database is included in appendix A for completeness, but is not otherwise discussed.

Usability of the 4P system will be assessed using a standard questionnaire adapted for our purpose

For peer review only

6. PARTICIPANT IDENTIFICATION

6.1. Study Participants

Pregnant women without significant cardiorespiratory comorbidity

6.2. Inclusion Criteria

a) ≥16 years of age (parental consent to be obtained for participants <18 years old)
b) Pregnant with an intent to deliver in hospital
c) Able to understand written or spoken English or has their own interpreter willing to translate
d) Willing and able to use the home monitoring equipment
e) Within category 1 of the American Society of Anaesthesiologists' classification of physical status at enrolment ("A normal healthy patient without any clinically important co-morbidity and without a clinically significant past/present medical history").

6.3. Exclusion Criteria

Any known medical condition expected by the recruiting clinician, at the recruitment visit, to alter maternal vital signs

7. STUDY PROCEDURES

7.1. Screening and Recruitment

For peer review only - http://bmjopen.bmj.com/site/about/guidelines.xhtml
© Copyright: The University of Oxford and Oxford University Hospitals NHS Trust 2013

Currently, in Oxford, women are recruited into the 4P study by the INTERBIO-21st study (REC ref. 08/H0606/139). Participants are recruited antenatally by the INTERBIO Research Midwife and join the 4P part of the study for the postnatal phase. There will be an overlap until March 2015, where some women are recruited through the INTERBIO study and remaining women through 4P.

In other sites, participants will be approached for recruitment when they attend their first ultrasound appointment at the hospital at around 12-14 weeks gestation. Maternal notes will be assessed for inclusion/exclusion criteria by a member of their direct care team OR a research midwife where ethical approval for this approach has already been granted, such as in Oxford (Oxford REC ref. 08/H060/139 version 3.9).

Women apparently meeting the criteria will then be approached by either a member of their usual care team or the research midwife at their first routine antenatal appointment or scan appointment. They will be given a 4P Study Information Leaflet and will be asked if they would mind talking to research staff who will be able to explain the study in more detail and take consent.

7.2. Informed Consent

The participant must personally sign and date the latest approved version of the Informed Consent form before any study specific procedures are performed.

Written and verbal versions of the Participant Information and Informed Consent will be presented to the participants detailing no less than: the exact nature of the study; what it will involve for the participant; the implications and constraints of the protocol; the known side effects and any risks involved in taking part. It will be clearly stated that the participant is free to withdraw from the study at any time for any reason without prejudice to future care, and with no obligation to give the reason for withdrawal.

The participant will be allowed as much time as wished to consider the information, and the opportunity to question the Investigator, their GP or other independent parties to decide whether they will participate in the study. Written Informed Consent will then be received by means of participant (or parental if under 18) dated signature and dated signature of the person who presented and obtained the Informed Consent. The person who received the consent must be suitably qualified and experienced, and have been authorised to do so by the Chief Investigator or one of their nominated local Principal Investigators. A copy of the signed Informed Consent will be given to the participant. A second copy will be sent to the co-ordinating centre in Oxford and the original signed form will be retained at the study site.

Recruitment of women between 16-18 years

Where young people under the age of 18 are involved in research, it is good practice to obtain consent from a parent or carer with parental responsibility in addition to obtaining consent from the young person. If a competent young person agrees to take part but specifically asks that her parents or guardians are not informed or involved in the discussion, and it is not possible to persuade them otherwise, then this should be respected. 4P has a parental consent form which may be used in conjunction with the participant consent form where the recruited participant is between 16-18 years of age.

For peer review only - http://bmjopen.bmj.com/site/about/guidelines.xhtml
© Copyright: The University of Oxford and Oxford University Hospitals NHS Trust 2013

7.3. Baseline Assessments

At the first visit (approximately 14-16 weeks gestation) between participant and research midwife, the following baseline assessments will be taken:

- Height *
- Weight *
- Date of birth *
- Ethnicity
- Haemoglobin concentration (where measured in clinical practice)*
- Number of previous pregnancies*
- Blood pressure, heart rate, temperature, oxygen saturations, respiratory rate.
    - Heart rate will be measured by palpating the radial pulse for one minute as well as using pulse oximetry. The manual count provides a cross-check for the electronic data as recommended by the Royal College of Nursing[12]
    - Respiratory rate will be recorded manually and by extrapolation from the accelerometry trace from a mobile device placed on the participant's chest whilst reclining. The mobile device will display software allowing the research midwife to record the time over which manual estimation of respiratory rate is made .Using the appropriate application on the tablet computer, the midwife will tap the tablet screen every time she counts a breath. This will provide an accurate date and time stamp of the participant's respiration.
    - All vital signs will be recorded twice as suggested by national measurement guidelines (British Hypertension Society)[13]
- Current medications*
- Smoking status*
- Medical History*

*Extracted from maternity notes. This information is collected as it will affect maternal vital signs.

For peer review only - http://bmjopen.bmj.com/site/about/guidelines.xhtml
© Copyright: The University of Oxford and Oxford University Hospitals NHS Trust 2013

7.4. Subsequent Visits throughout pregnancy

Participants will be seen at approximately 4-6 week intervals throughout pregnancy when they attend their usual ante-natal clinic appointments. This will add approximately 15 minutes to each visit. In the case where appointments are more than six weeks apart, or the study observations could not be taken at the usual appointment, a qualified midwife will arrange to meet at the participants' convenience.

- At these visits the research midwives will take a full data set: Blood pressure, heart rate, temperature, oxygen saturations and respiratory rate.
    - Respiratory rate will be recorded manually and by extrapolation from the accelerometry trace from a mobile device placed on the participant's chest whilst reclining. The mobile device will display software allowing the research midwife to record the time over which manual estimation of respiratory rate is made.
- Medical diagnoses since last visit will be recorded, for example:
    - Diabetes, thyroid disease or any other endocrinological condition
    - Respiratory Disease
    - Renal Disease including pyelonephritis
    - Hepatic Disease
    - Gastrointestinal disease
    - Autoimmune conditions
    - Haematological conditions
    - Infection requiring antibiotics treatment
- Pregnancy-related Complications will be recorded
    - Severe vomiting requiring hospital admission
    - Gestational diabetes
    - Pregnancy-induced hypertension (blood pressure>140/90, no proteinuria)
    - Pre-eclampsia (blood pressure >140/90 AND >/= to 2+ proteinuria or >30mg/dl)
    - Severe Pre-eclampsia/Eclampsia/HELLP syndrome
- Medication changes will be recorded

This information will be added to the study data base via a tablet computer.

If any abnormal vital signs are identified during the follow up visits, the research midwife will refer the participant to their usual midwife, GP or local maternity hospital assessment unit.

7.5 Intrapartum

- Pregnancy-related complications as above will be **retrospectively** extracted from the patient's notes
- Blood pressure, heart rate, temperature, oxygen saturations and respiratory rates and oxygen supplementation recorded during the in-hospital intra-partum period will be retrospectively extracted from the patient's notes

For peer review only - http://bmjopen.bmj.com/site/about/guidelines.xhtml
© Copyright: The University of Oxford and Oxford University Hospitals NHS Trust 2013

For peer review only

- Dates and times of stages of labour and birth will be retrospectively extracted from the patient's notes
- Uterotonic drugs given in the third stage of labour will be retrospectively extracted from the patient's notes
- Episodes of post-partum haemorrhage, blood transfusion and surgical intervention will be retrospectively extracted from the patient's notes
- Mode of delivery will be recorded (extracted from notes)
- Epidural use including timings will be recorded (extracted from notes)
- Type of anaesthesia (epidural, spinal, general, etc) (extracted from notes)

This clinical information is gathered as it will affect maternal vital signs.

7.6 Postnatal

- The date of discharge will be recorded
- The lowest measured postnatal haemoglobin prior to discharge will be retrospectively extracted from the patient's notes
- Post-natal blood transfusions (number of units and timings)
- Medications on discharge, including antihypertensive agents and antibiotics will be retrospectively extracted from the patient's notes

Home Monitoring

After delivery participants will be issued with a home monitoring kit in order to monitor their own vital signs once a day at any time, for approximately 2 weeks (see below).

Clinical information is gathered as it will affect vital signs.

The 4P home monitoring kit

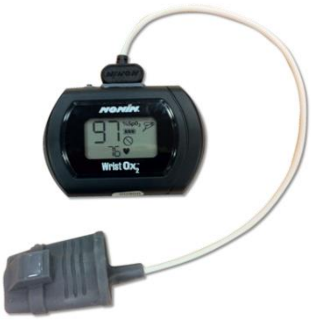

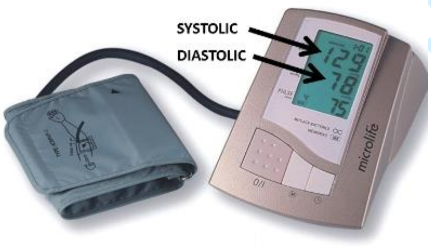

PULSE OXIMETER FINGER PROBE (DEVICE AND SENSOR) BLOOD PRESSURE METER

For peer review only

For peer review only - http://bmjopen.bmj.com/site/about/guidelines.xhtml
© Copyright: The University of Oxford and Oxford University Hospitals NHS Trust 2013

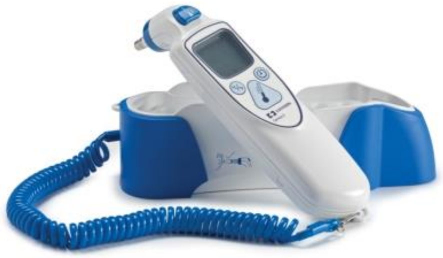

TYMPANIC (EAR) THERMOMETER

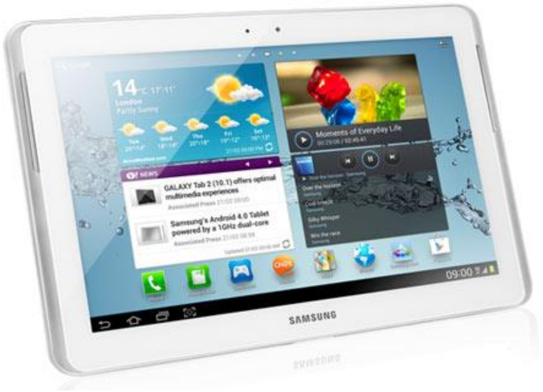

TABLET

Participants will be taught the correct use of all the equipment, using a Standard Operating Procedure developed by the 4P midwives (SOP NO.1 –Teaching Kit Use to Study Participants) at a visit near the time of delivery.

The tablet software will instruct the participant on the use of the equipment and the sequence of measurements and allow participant feedback.

The tablet will contain the hospital advice sheets normally available to women within the service. Information will be specific to the centre and will have been approved by the centres standard processes (for example in Oxford, these will include advice on blood clot prevention, breastfeeding, bottle feeding, safe sleeping, prevention of cot death, easing perineal discomfort, postnatal depression, smoking cessation, healthy diet, pelvic floor exercises and exercise after child birth. The information has been taken from the hospital trust's internet site where the information has been approved by the hospital's local policy and is freely available to the general public).

The tablet allows participants to send study-related queries to the research midwives.

The vital signs will be securely transferred either via Wi-Fi or 3G/4G to the study database. Transferred vital signs will be linked to the woman's study ID number, but will not contain any patient-identifiable information.

Research midwives will review the data and contact the participant by telephone if data is not being received. The research midwives will visit the participant at home to resolve any equipment difficulties.

The research midwife will, where possible, arrange to visit at least once during the two week period to undertake a vital sign set and re-enforce measurement techniques.

For peer review only - http://bmjopen.bmj.com/site/about/guidelines.xhtml
© Copyright: The University of Oxford and Oxford University Hospitals NHS Trust 2013

A high blood pressure reading will cause a message to appear onscreen, asking the participant to repeat the measurement in half an hour. If the measurement is still high, a message will appear, asking her to access medical help via her local maternity unit.

All participants will be made aware that the information collected by the home monitoring phase of the study is not reviewed in real time and that it remains their responsibility to seek assistance if they feel unwell.

The research midwife will visit to undertake a final vital sign set and collect the 4P equipment around day 14 post-delivery. During this visit, the participant will be asked to fill a usability questionnaire. The questionnaire will be based on the System Usability Scale questionnaire[10,11] .
If abnormal vital signs are identified at the home visits, the research midwife will refer the participant to her usual midwife, GP or local maternity hospital assessment unit.

7.7 Discontinuation/Withdrawal of Participants from Study

In accordance with the current revision of the Declaration of Helsinki (amended October 2000, with additional footnotes added in 2002 and 2004), woman have the right to withdraw from the study at any time and for any reason, without prejudice to their future medical care. Participants are not obliged to give any reason for this. Should this happen the woman will offered the choice of allowing the data already collected to be used within the study, or to have all her data withdrawn from the study.

The reason for withdrawal will be recorded in the participant tracking log (see below).

7.8 Definition of End of Study

The end of the study is defined as when the database has been completed, cleaned and deemed final and ready for analysis.

8. STATISTICS AND ANALYSIS

8.1. The Number of Participants

1000 patients will provide a convenience sample which, allowing for a 10% dropout rate and a 10% development of abnormality rate will leave 800 patients from whom to derive centile-based distributions.

8.2. Description of Statistical Methods

For each physiological variable (HR, RR, SpO2, Temp, Systolic BP, Diastolic BP), for each study site and for the study sites combined, normalised histograms (unit area under the curve) and cumulative distribution functions will be plotted.

Where necessary, a kernel-based density estimator will be used to estimate smoothed distributions.

Interim analyses are not required (as there is no intervention).

For peer review only

For peer review only - http://bmjopen.bmj.com/site/about/guidelines.xhtml
© Copyright: The University of Oxford and Oxford University Hospitals NHS Trust 2013

8.3. Analysis of Outcome Measures/Endpoints

An automatic, multi-parameter, centile-based pregnancy-stage specific alerting system will be created using a data-driven probabilistic model of normality. An early warning system calculator will be developed which will show the effect of centile threshold changes for each vital sign for a given observation rate. An expert panel will then balance the expected alert rate against the acceptable degree of abnormality for each physiological variable. Each considered threshold will be given with an estimate frequency of that threshold in the "normal" population.

Additional variables will be used; to describe the population under study, to assess whether the inclusion of values from patients with known factors, which may affect the readings, alters the distributions significantly.

9. DATA MANAGEMENT

9.1. Access to Data

Direct access will be granted to authorised representatives from the Sponsor or host institution for monitoring and/or audit of the study to ensure compliance with regulations.

9.2. Data Recording and Record Keeping

.

For the purposes of analysis each participant will be given an individual study number when they consent to take part in the study. The study number will be kept with the patient's name, hospital number and demographic data on the master log. This will be held on the secure NHS server farm. The master log will be held separately from the 4P database and will only be accessible within the secure research facility. The server stores sensitive data (Names, addresses, DOB) using data encryption as an additional level of protection. Access to the master log will be restricted (by username and 8 digit password, including alphanumeric and special characters) to the Chief Investigator, the local Principal Investigator and research staff directly involved with the project. The master log will be destroyed 21 years after the study has ended.

All study data (other than that contained in the master log) will be entered into the secure 4P database through one of two separate routes; vital sign information will be submitted through tablet computers running the 4P Android application. All other information, such as clinical outcomes, will be entered through the secure 4P web interface, which is only accessible with a username and password. Within the database, each participant's data will be linked to their study identification number. No other patient-identifiable information will be held in the database. Each data transfer is pseudonymised.

The system architecture is shown in figure 1 The database and web interfaces will be held on a secure virtual server hosted by Oxfordshire Health Informatics Service which resides within the NHS network and behind the NHS firewall. The virtual server will run Windows 2008 Server edition or an appropriate upgrade. The webserver will be Microsoft IIS and the database will be MySQL (or appropriate upgrades). The server will handle every communication using a valid SSL certificate authenticated by DigiCert (High

For peer review only - http://bmjopen.bmj.com/site/about/guidelines.xhtml
© Copyright: The University of Oxford and Oxford University Hospitals NHS Trust 2013

For peer review only

Assurance certificate) with 128 bit encryption. Access to the 4P secure web interfaces will be restricted (by username and 8 digit password, including alphanumeric and special characters) to the Chief Investigator, the local Principal Investigator and research staff directly involved with the project at each study site. Researchers from each site can only view and access data of participants from that hospital site. Access to the secure web interface will allow research staff to support participants in undertaking home monitoring. All activity and attempts to access the 4P secure interface, as well as all communication from the tablet computers will be logged.

No data will be stored on the tablet computer. At the end of each data recording session, the file containing the vital sign information is transmitted to the main 4P database and deleted from the tablet memory. If at any point transmission of files from the tablets computers fail, the data is temporarily stored on the tablet and automatically transmitted (and deleted) as soon as a 3G/4G or secure Wi-Fi connection is restored. Should this happen, the research midwives will be able to detect that temporary storage has occurred when they access the secure 4P Web interface. Once transmission has occurred the data will be automatically deleted. All tablet computers will have the facility for the memory to be remotely wiped in the case of loss of the equipment.

A participant tracking log including patient identifiable data will be kept within the secure research area. The tracking log will only be used by researchers for the 4P study. The tracking log will be kept securely for 21 years after the study has ended, after which it will be destroyed.

All paperwork will be stored in a secure research environment behind a security locked door in a locked cabinet throughout the duration of the study.

Antenatal data

At each patient encounter during pregnancy, data recorded on a tablet computer running the 4P Android application will be transmitted via a secure NHS Wi-Fi connection during clinic visits, or via 3G/4G during visits to the participant's home. After the data has been transmitted, it is automatically deleted from the tablet computer so that no data can be viewed on the tablet computer.

The same principles apply to accelerometry data recorded on the smartphone (use to estimate respiratory rate).

In Hospital Data Collection

All in hospital data for each participant will be photocopied from their maternity notes before the notes are sent to the hospital storage facility. Photocopying the notes is necessary both to allow efficient data entry and to ensure that the notes remain easily available for clinical use.

The photocopied notes will be anonymised by obscuring all patient identifiers at the point of photocopying or cutting it off the copies and shredding it. The study number will be recorded on each photocopy. Two copies will be made in case one copy is destroyed by adverse events such as fire or water damage. The copies will be held separately in locked cabinets within secure research areas.

For peer review only

For peer review only - http://bmjopen.bmj.com/site/about/guidelines.xhtml
© Copyright: The University of Oxford and Oxford University Hospitals NHS Trust 2013

Notes photocopied are:

The partogram (labour data record from which blood pressure, heart rate and temperature will be extracted)

The anaesthetic chart (from which instrumental deliveries, caesarean sections and perineal repair, heart rate, temperature and oxygen saturations are extracted)

The MEOWS charts (from which heart rate, temperature, oxygen saturations and respiratory rate are extracted)

All chart data above are entered onto the 4P database through the secure web interface by the 4P midwives, from a computer connected to each hospital's secure network within the secure research area. The study participants are only identified by their study number in the study database. Chart data will be entered twice, by separate researchers to ensure accuracy. The second time the data is entered it will automatically be compared with the first and discrepancies highlighted. Access to the secure web interface for purposes of data entry and comparison will be restricted (by user log in with a user name and passwords to be a minimum of 8 characters including both upper and lower case alphanumeric characters).

The anonymised photocopies will be kept securely for 10 years, after which they will be destroyed by shredding.

Post-partum (at home) data collection

Post-partum data will be collected by the participants using the home monitoring equipment described above. Before the equipment is given to the patient, the tablet computer will be configured with the participant's unique study ID which is hidden from the user in the application (viewing and changing the study ID will require username and 8 digit password access, including alphanumeric and special characters and will be restricted to research staff). The vital signs data will be recorded on the tablet through a 4P Android application. Completed recordings which contain the vital sign recordings (purely numerical), study ID (pseudonymised) and the date of the recording will be transferred securely via 3G/4G to the secure 4P study database. Recordings will be automatically deleted from the tablet after transmission and no vital sign data will be stored on the home monitoring tablet. The participant ID will be removed from the tablet at the end of the two week recording period and the tablet memory will be wiped. There will be no access to the secure 4P research database from the home monitoring secure web interface.

When a research midwife visits a participant at home during the postnatal period, she will use the same tablet application and data security procedures as those described under the section for antenatal data collection.

Once data collection is complete, the secure pseudonymised 4P database will be transferred to the University of Oxford high compliance system, which allows secure storage and access to sensitive data.

The database will be destroyed after 21 years.

For peer review only

For peer review only - http://bmjopen.bmj.com/site/about/guidelines.xhtml
© Copyright: The University of Oxford and Oxford University Hospitals NHS Trust 2013

In summary, participant identifiable data and the 4P database will be stored for 21 years after the end of the study and then destroyed.

The anonymised/pseudonymised photocopies will be stored securely for 10 years and then destroyed.

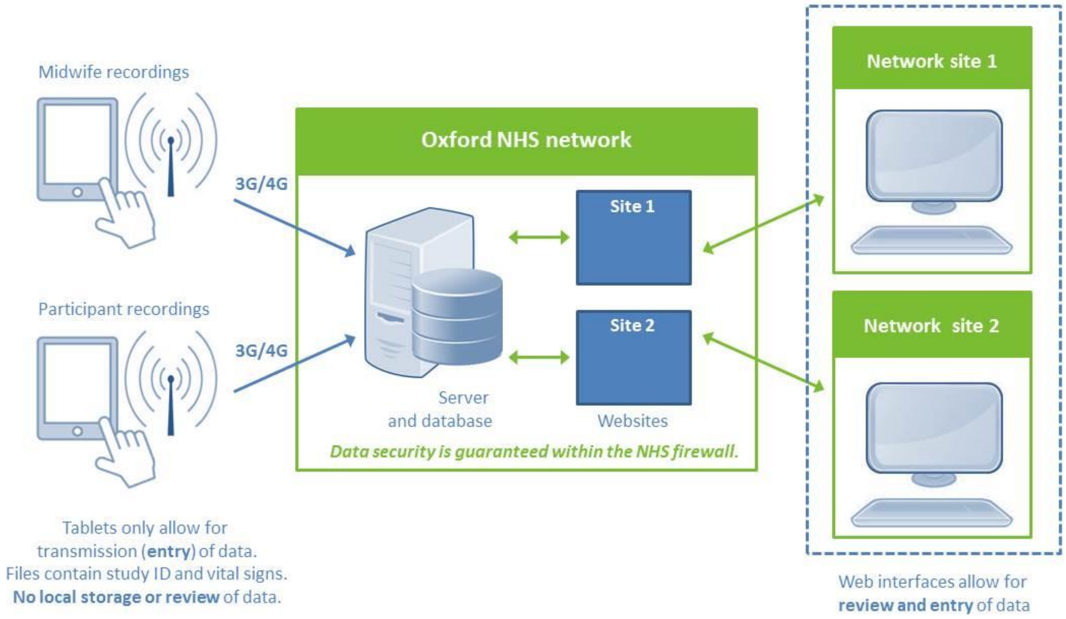

Figure 1. N.B. Whilst there is no tablet local storage of data under normal circumstances, temporary storage may occur if there is transmission failure (see text above). Access from clients to the 4P secure web interface is based on filtering of pre-approved IP addresses at each hospital site.

10. QUALITY ASSURANCE PROCEDURES

The study may be monitored, or audited in accordance with the current approved protocol, ICH GCP, relevant regulations and standard operating procedures.

11. ETHICAL AND REGULATORY CONSIDERATIONS

Participants in the 4P study will be observed more frequently than they would be as per usual maternal health care. However, the opportunity to meet with health professionals more frequently can also be beneficial and participants in the Interbio research study in Oxford (Ethics reference REC 08/HO6O/139) have not found the study visit schedule to be onerous.

For peer review only - http://bmjopen.bmj.com/site/about/guidelines.xhtml
© Copyright: The University of Oxford and Oxford University Hospitals NHS Trust 2013

Participants in the study will be asked to take their own physiological measures at home. There is the possibility that some measures will be abnormal. The software on the home monitoring equipment will advise study participants to contact their local maternity unit if elevated blood pressure values are recorded.

11.1. Declaration of Helsinki

The Investigator will ensure that this study is conducted in accordance the principles of with the Declaration of Helsinki (last amended October 2013).

11.2. Guidelines for Good Clinical Practice

The Investigator will ensure that this study is conducted in accordance with relevant regulations and with Good Clinical Practice.

11.3. Approvals

The protocol, informed consent form, participant information sheet and any proposed advertising material will be submitted to an appropriate Research Ethics Committee (REC), and host institution(s) for written approval.

The Investigator will submit and, where necessary, obtain approval from the above parties for all substantial amendments to the original approved documents.

11.4. Reporting

The CI shall submit once a year throughout the study, or on request, an Annual Progress report to the REC Committee, host organisation and Sponsor. In addition, an End of Study notification and final report will be submitted to the same parties.

11.5. Participant Confidentiality

Personal data and sensitive information required for the study will be collected directly from trial participants and hospital notes. All personal information received in paper format for the trial will be held securely and treated as strictly confidential. All staff involved in the study share the same duty of care to prevent unauthorised disclosure of personal information. No data that could be used to identify an individual will be published. Data will be entered and stored on a password protected, access restricted, secure server at the University of Oxford under the provisions of the Data Protection Act and/or applicable laws and regulations. Patient information collected electronically as part of the study will be held securely and treated as confidential. This information can also be requested at any time following usual Trust protocols for the requesting of patient's notes.

The study staff will ensure that the participants' anonymity is maintained. The participants will be identified only a participants ID number on the CRF and any electronic database. All documents will be stored securely and only accessible by study staff and authorised personnel. The study will comply with the Data Protection Act, which requires data to be anonymised as soon as it is practical to do so.

For peer review only - http://bmjopen.bmj.com/site/about/guidelines.xhtml
© Copyright: The University of Oxford and Oxford University Hospitals NHS Trust 2013

11.6. Expenses and Benefits

Reasonable travel expenses for any visits additional to normal care will be reimbursed on production of receipts, or a mileage allowance provided as appropriate.

A £20 gift voucher will be issued on completion of the home monitoring phase.

12. FINANCE AND INSURANCE

12.1. Funding

This study is supported by the NIHR Biomedical Research Council.

14.2 Insurance

The University has a specialist insurance policy in place which would operate in the event of any participant suffering harm as a result of their involvement in the research (Newline Underwriting Management Ltd at, Lloyd's of London). NHS Indemnity operates in respect of the clinical treatment which is provided.

13. PUBLICATION POLICY

The Investigators will be involved in reviewing drafts of the manuscripts, abstracts, press releases and any other publications arising from the study. Authors will acknowledge that the study was funded by the NIHR BRC. Authorship will be determined in accordance with the ICMJE guidelines and other contributors will be acknowledged.

For peer review only

For peer review only - http://bmjopen.bmj.com/site/about/guidelines.xhtml
© Copyright: The University of Oxford and Oxford University Hospitals NHS Trust 2013

14. REFERENCES

1. Saving Mothers' Lives: Reviewing maternal deaths to make motherhood safer - 2003-2005. The Seventh Report of the Confidential Enquiries into Maternal Deaths in the United Kingdom. 2007.
2. Saving Mothers' Lives: Reviewing maternal deaths to make motherhood safer: 2006–2008. The Eighth Report of the Confidential Enquiries into Maternal Deaths in the United Kingdom. 2011.
3. Swanton RD, Al-Rawi S, Wee MY. A national survey of obstetric early warning systems in the United Kingdom. International journal of obstetric anesthesia 2009;18:253-7.
4. Carle C, Alexander P, Columb M, Johal J. Design and internal validation of an obstetric early warning score: secondary analysis of the Intensive Care National Audit and Research Centre Case Mix Programme database. Anaesthesia 2013;68:354-67.
5. Smith GB, Prytherch DR, Meredith P, Schmidt PE, Featherstone PI. The ability of the National Early Warning Score (NEWS) to discriminate patients at risk of early cardiac arrest, unanticipated intensive care unit admission, and death. Resuscitation 2013;84:465-70.
6. Macdonald-Wallis C, Lawlor DA, Fraser A, May M, Nelson SM, Tilling K. Blood pressure change in normotensive, gestational hypertensive, preeclamptic, and essential hypertensive pregnancies. Hypertension 2012;59:1241-8.
7. Magriples U, Boynton MH, Kershaw TS, Duffany KO, Rising SS, Ickovics JR. Blood pressure changes during pregnancy: impact of race, body mass index, and weight gain. American journal of perinatology 2013;30:415-24.
8. Tarassenko L, Clifton DA, Pinsky MR, Hravnak MT, Woods JR, Watkinson PJ. Centile-based early warning scores derived from statistical distributions of vital signs. Resuscitation 2011;82:1013-8.
9. King S, Bannister PR, Clifton DA, Tarassenko L. Probabilistic Approaches to Condition Monitoring of Aerospace Engines. Journal of Aerospace Engineering 2009;223:533-41.

10 Brooke, J. (2013) SUS: a retrospective. Journal of Usability Studies. 8(2):29-40

11. Bangor, A, Kortum, K. Miller, J. (2009) Determining what individual SUS scores mean:adding an adjective rating scale. Journal of Usability Studies 4(3):114-123

12. Royal College of Nursing, "Standards for assessing, measuring and monitoring vital signs in infants, children and young people."[Accessed online, 10 July 2015] http://www.rcn.org.uk/__data/assets/pdf_file/0004/114484/003196.pdf, 2013

13 E. O'Brien, R. Asmar, L. Beilin, Y. Imai, J.-M. Mallion, G. Mancia, T. Mengden, M. Myers, P. Padfield, P. Palatini, et al.,"European society of hypertension recommendations for conventional, ambulatory and home blood pressure measurement,"Journal of hypertension, vol. 21, no. 5, pp. 821–848, 2003.

For peer review only

For peer review only - http://bmjopen.bmj.com/site/about/guidelines.xhtml
© Copyright: The University of Oxford and Oxford University Hospitals NHS Trust 2013

15. APPENDIX A: STUDY FLOW CHART

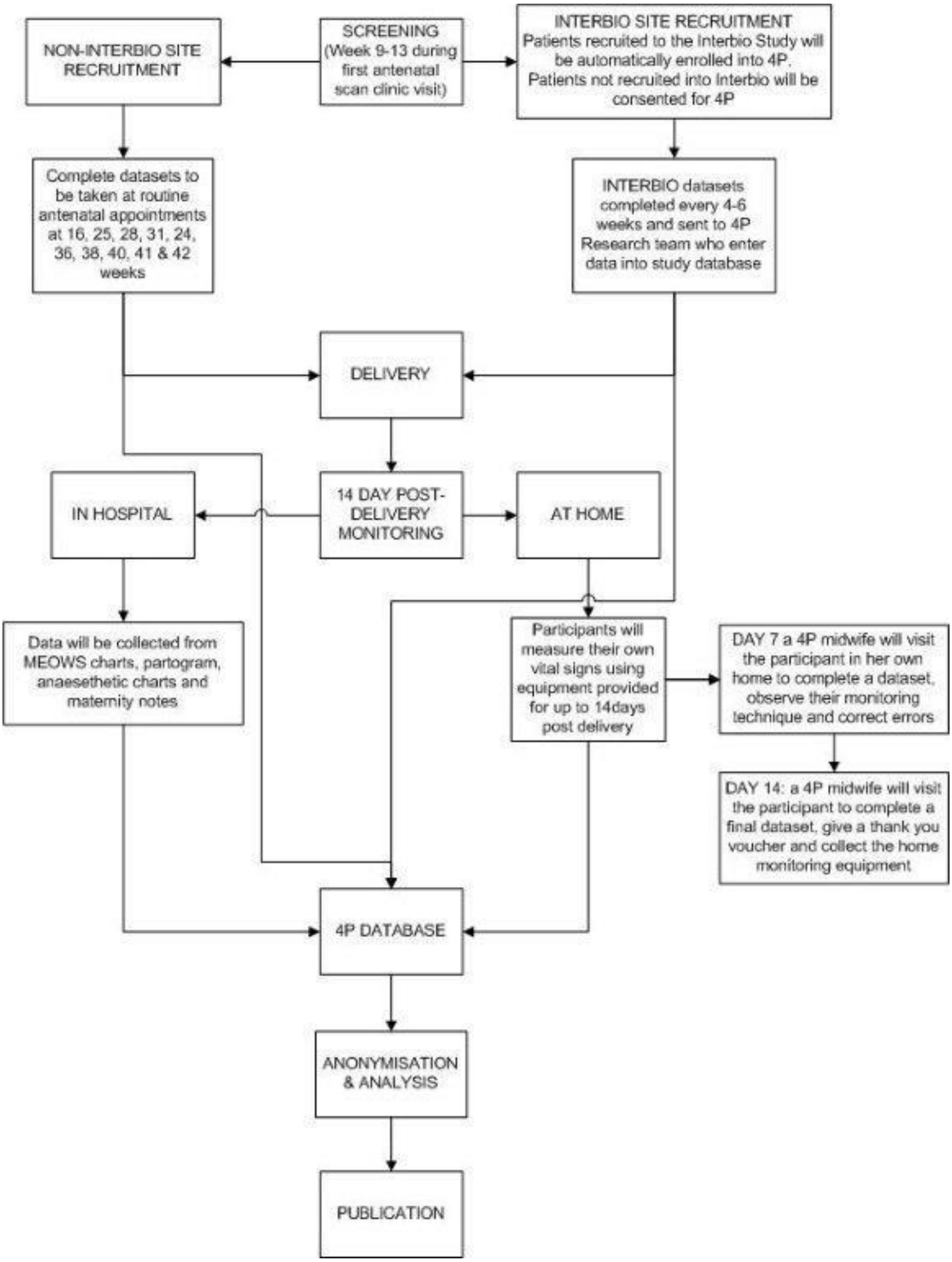

For peer review only - http://bmjopen.bmj.com/site/about/guidelines.xhtml
© Copyright: The University of Oxford and Oxford University Hospitals NHS Trust 2013

16. APPENDIX B: SCHEDULE OF STUDY PROCEDURES

**Procedures**	**Visits**				
	**Screening**	**Baseline**	**Follow ups visits in pregnancy**	**Home Monitoring of vital signs**	**Postnatal home visits by research midwives**
Informed consent	1	1			
Demographics		1			
Medical history	1		8		2
Physical examination	0				
ECG	0				
Laboratory tests	0				
Eligibility assessment	1				
Randomisation	N/A				
Assessment 1 -8 (Antenatal vital signs recording by research midwife)			8		
Assessment 9-23 (Postnatal vital signs recordings by participant for 2 weeks)				14	
Assessment 24+25 (Home visits by research midwife)					2

For peer review only - http://bmjopen.bmj.com/site/about/guidelines.xhtml
© Copyright: The University of Oxford and Oxford University Hospitals NHS Trust 2013

For peer review only

17. APPENDIX C: AMENDMENT HISTORY

Amendment No.	Protocol Version No.	Date issued	Author(s) of changes	Details of Changes made
1	2.0	12/01/2015	Dr Peter Watkinson	Page numbering due to additional text<br>To include ethnicity as part of demographic information recorded<br>Section 6.3 to clarify that exclusion criteria only applicable at recruitment visit<br>Data managing details amended to reflect lack of access to NHS computers at other sites.<br>Inclusion of updated system architecture diagram<br>Addition of Usability Questionnaire
2	3.0	14/09/2015	Dr Peter Watkinson	Inserted details of vital signs measurements including a one minute manual pulse palpation and repetition of all vital signs measurements

For peer review only

For peer review only - http://bmjopen.bmj.com/site/about/guidelines.xhtml
© Copyright: The University of Oxford and Oxford University Hospitals NHS Trust 2013

BMJ Open

The Pregnancy Physiology Pattern Prediction Study (4P Study): protocol of an observational cohort study collecting vital sign information to inform the development of an accurate centile-based obstetric early warning score

Journal:	*BMJ Open*
Manuscript ID	bmjopen-2017-016034.R1
Article Type:	Protocol
Date Submitted by the Author:	21-Jun-2017

Complete List of Authors:	Kumar, Fiona; Oxford University Hospitals NHS Foundation Trust, Nuffield Department of Obstetrics and Gynaecology<br>Kemp, Jude; Oxford University Hospitals NHS Foundation Trust, Nuffield Department of Obstetrics and Gynaecology<br>Edwards, Clare; Oxford University Hospitals NHS Foundation Trust, Nuffield Department of Obstetrics and Gynaecology<br>Pullon, Rebecca; University of Oxford, Engineering Science<br>Loerup, Lise; University of Oxford, Engineering Science<br>Triantafyllidis, Andreas; University of Oxford, Engineering Science<br>Salvi, Dario; University of Oxford, Engineering Science<br>Gibson, Oliver; University of Oxford, Engineering Science<br>Gerry, Stephen; University of Oxford, Centre for Statistics in Medicine<br>MacKillop, Lucy; Oxford University Hospitals NHS Trust, Nuffield Department of Obstetrics & Gynaecology<br>Tarassenko, Lionel; University of Oxford, Engineering Science<br>Watkinson, Peter; Oxford University Hospitals NHS Trust, Nuffield Department of Clinical Neurosciences

**Primary Subject Heading**:	Obstetrics and gynaecology
Secondary Subject Heading:	Evidence based practice, Emergency medicine, Intensive care
Keywords:	pregnancy, vital signs, early warning score

SCHOLARONE™
Manuscripts

The Pregnancy Physiology Pattern Prediction Study (4P Study): protocol of an observational cohort study collecting vital sign information to inform the development of an accurate centile-based obstetric early warning score

Fiona Kumar[1], Jude Kemp[1], Clare Edwards[1], Rebecca M. Pullon[2], Lise Loerup[2], Andreas Triantafyllidis[2], Dario Salvi[2], Oliver Gibson[2], Stephen Gerry[3], Lucy H. Mackillop[1], Lionel Tarassenko[2] and Peter J. Watkinson[4]

[1]Nuffield Department of Obstetrics & Gynaecology, Oxford University Hospitals NHS Trust, Oxford, UK

[2]Department of Engineering Science, University of Oxford, Oxford, UK

[3]Centre for Statistics in medicine, Nuffield Department of Orthopaedics, Rheumatology and Musculoskeletal Sciences, University of Oxford, Oxford, UK

[4] Kadoorie Centre for Critical Care Research and Education, Nuffield Department of Clinical Neurosciences, Oxford University Hospitals NHS Trust, Oxford, UK

Corresponding author:

Peter J Watkinson

Postal address: Kadoorie Centre for Critical Care Research and Education, Level 3, John Radcliffe Hospital, Headley Way Oxford, OX3 9DU.

Email: peter.watkinson@ndcn.ox.ac.uk

Phone: 01865 617720

Key words: pregnancy, vital signs, early warning score

Word count: 3300 (main text, excluding references and appendices)

For peer review only

ABSTRACT

Introduction: Successive Confidential Enquiries into Maternal Deaths in the UK have identified an urgent need to develop a national Early Warning Score (EWS) specifically for pregnant or recently pregnant women to aid more timely recognition, referral and treatment of women who are developing life-threatening complications in pregnancy or the puerperium. Although many local EWS are in use in obstetrics, most have been developed heuristically. No current obstetric EWS has defined the thresholds at which an alert should be triggered using evidence-based normal ranges, nor do they reflect the changing physiology that occurs with gestation during pregnancy.

Methods and analysis: An observational cohort study involving 1000 participants across three UK sites in Oxford, London and Newcastle. Pregnant women will be recruited at approximately 14 weeks gestation and have their vital signs (heart rate, blood pressure, respiratory rate, oxygen saturation and temperature) measured at 4-6 week intervals during pregnancy. Vital signs recorded during labour and delivery will be extracted from hospital records. After delivery, participants will measure and record their own vital signs daily for two weeks. During the antenatal and postnatal periods, vital signs will be recorded on an Android tablet computer through a custom software application and transferred via mobile internet connection to a secure database. The data collected will be used to define reference ranges of vital signs across normal pregnancy, labour and the immediate post-natal period. This will inform the design of an evidence-based obstetric early warning score.

Ethics and dissemination: The study has been approved by the NRES committee South East Coast – Brighton and Sussex (14/LO/1312) and is registered with the ISRCTN (10838017). All participants will provide written informed consent and can withdraw from the study at any point. All data collected will be managed anonymously. The findings will be disseminated in international peer-reviewed journals and through research conferences.

For peer review only

For peer review only - http://bmjopen.bmj.com/site/about/guidelines.xhtml

STRENGTHS AND LIMITATIONS OF THIS STUDY

- This will be the first study to describe normal ranges of all five vital signs (blood pressure, heart rate, temperature, respiratory rate and oxygen saturation) gathered contemporaneously across gestation in the antepartum, intrapartum and in the postpartum periods.
- Participants will all be within UK healthcare systems. This may limit translation to other healthcare settings.

For peer review only

For peer review only - http://bmjopen.bmj.com/site/about/guidelines.xhtml

BACKGROUND

Early warning scoring systems (EWS) for obstetric patients assign increasing scores (or colours) to each individual physiological measurement (blood pressure, heart rate, temperature, respiratory rate and oxygen saturation) as they become more abnormal. If either the sum of scores for several physiological measurements, or a score for a single physiological measurement exceed set thresholds, an alert is triggered. Successive Confidential Enquiries into Maternal Deaths in the UK have highlighted an urgent need to develop a national obstetric EWS to aid the more timely recognition, treatment and referral of women who are developing life-threatening complications of pregnancy[1, 2, 3]. This recommendation was strongly supported by a national survey of obstetric anaesthetists[4]. It is acknowledged that recognising physiological deterioration is complicated by the normal changes in maternal physiology that occur both during pregnancy and immediately after delivery[2].

An evidence-based approach is required for widespread acceptance of a national obstetric early warning scoring system[5]. Recognising normality is the first step to understanding abnormality[6]. To understand normal ranges at different gestational ages of "normal" pregnancy, the intrapartum and the post-partum period, large numbers of measurements at each point are required. These will allow normal ranges and their associated centiles throughout pregnancy to be defined in the modern era. It is important that these observations are measured at the same time to allow their interactions to be understood. Longitudinal data recorded from the same patients, where possible with the same equipment, across the entire pregnancy and the first two weeks after delivery is particularly important to ensure that step-changes between the stages of pregnancy are not missed.

Large amounts of longitudinal data for blood pressure during pregnancy already exist (for example from the longitudinal AVON[7] and BOSHI[8] studies). However much of this is now more than two decades old. We found less data for heart rate in pregnancy (for example[9, 10, 11]). Relatively few data have been collected during the intrapartum period or for the first two weeks postpartum (for example if postnatal data are collected, this only occurs at one or two occasions several weeks after delivery, such as at six weeks in[12, 13], and 6 months in[14]). Furthermore, there are very few data available for temperature (for example[15, 16]), respiratory rate (for example[17, 18]) and oxygen saturation (for example[17, 19]) in pregnancy, intrapartum or postpartum, from which to define modern normal-range centiles. The data that are available suggest clear trends occur during pregnancy which may need to be taken into account to ensure appropriate identification of abnormality. For example systolic blood pressure appears to change by around 6mmHg over the course of pregnancy[7, 8]. However, while clear vital sign trends are apparent, the range of expected vital sign values during pregnancy are not known, and have not been able to be determined by synthesis of available data sets.

For peer review only - http://bmjopen.bmj.com/site/about/guidelines.xhtml

For peer review only

A centile-based approach to identifying abnormality will allow experts to choose which women should be reviewed, based on strong evidence of how far one or more of their vital-sign observations lie from normality. The approach is particularly suited to the analysis of data recorded during pregnancy, as well as intrapartum and postpartum, during which the event rate is relatively low. We have previously used such an approach for both the recognition of physiological deterioration in hospitalised adults [6] and the maintenance of safety in the aerospace industry [20].

The primary objective of this study is to develop a database of vital sign measurements from pregnancy, labour and the postpartum period (Table 1). Estimates of population distributions and associated centiles can be derived from this database. The secondary objective of this study is to use this information to develop a centile-based early warning scoring system for pregnancy, labour and the postpartum period, that will guide clinical experts to provide the most appropriate care.

	**Objectives**	**Endpoints**
**Primary**	• To develop a database of vital sign measurements during pregnancy, labour and the postpartum period	• Estimates of distributions and associated centiles for five vital signs throughout pregnancy, labour and the postpartum period
**Secondary**	• To develop a centile-based early-warning scoring system for pregnancy, labour and the post-partum period<br>• To investigate new patterns within vital sign data in pregnancy	• Centile-based thresholds for alerting for five vital signs throughout pregnancy, labour and the postpartum period<br>• Trend analyses of five vital signs and inter-relations between these trends

Table 1: A summary of the primary and secondary objectives and endpoints

For peer review only - http://bmjopen.bmj.com/site/about/guidelines.xhtml

METHODS AND ANALYSIS

We prepared this protocol following the Strobe and SPIRIT guidelines, where appropriate.

Study design: A UK three-centre longitudinal observational study. The study commenced August 2012. We will continue the study until 1000 women contribute vital sign data and the last participant is 14 days post-partum (estimated completion September 2017). The study will take place in two stages. Stage 1, in a single centre (Oxford) as a sub-study of the Interbio-21st study (REC:08/H0606/139)[21]. Stage 2 will allow two additional centres and the continuation of the study in the first centre after the completion of the Interbio-21st study. Study procedures will be unchanged between phases.

Study setting: Women will be approached to take part when they attend the ultrasound department or antenatal clinics at three university hospitals (Appendix 1). Data will be collected from antenatal visits, hospital admissions and the community.

Study population: We aim to recruit 1000 women aged 16 or over, with a singleton pregnancy of less than 20 weeks gestation, who fall within category 1 of the American Society of Anaesthesiologists' classification of physical status at enrolment ("A normal healthy patient without any clinically important co-morbidity and without a clinically significant past/present medical history"). Gestational age was determined using a measurement of crown-rump length obtained prior to 14 weeks of gestation[22]. We will exclude women with a known condition expected by the recruiting clinician to alter maternal vital signs. The full inclusion and exclusion criteria are detailed in Appendix 2.

Vital sign data collected: Five vital signs will be collected: blood pressure, heart rate, oxygen saturation, temperature and respiratory rate. Blood pressure will be measured with a blood pressure monitor approved for use in pregnancy (Microlife 3BT0-A(2)/WatchBP Home, Microlife, Taipei, Taiwan). Heart rate and oxygen saturation will be measured with a Bluetooth-enabled pulse oximeter (WristOx2 3150, Nonin Medical, Minnesota, USA). Temperature is measured with a tympanic thermometer (Genius™ 2, Covidien (Medtronic), Dublin, Ireland). In a subgroup, temperature will be measured with an additional Bluetooth thermometer (Fora IRb 20b, ForaCare, Taipei, Taiwan). The device allows for automatic transfer of readings (thus eliminating the risk of user input errors) but is not commonly used in UK clinical practice. Trained midwives will estimate respiratory rate by observing chest wall movement following a standard operating procedure. A software application on an Android smartphone will also be used to estimate respiratory rate through the detection of chest-wall movements with the in-built accelerometer and gyroscope. The equipment used for data collection is shown in Figure 1.

Vital sign data will be entered (or in the case of Bluetooth-enabled devices, automatically transmitted), onto a tablet computer (Samsung Galaxy Tab 4.0) and sent to a secure database on the NHS network using

For peer review only - http://bmjopen.bmj.com/site/about/guidelines.xhtml

3G/4G. The tablet computer will run a custom Android application that will guide the user through the measurement sequence. Once sent, the data will be automatically deleted from the tablet. All data will be entered, transferred and stored using a unique study number.

Vital sign data recorded during delivery will be extracted retrospectively from the participants' hospital notes once they have been discharged from hospital. All vital signs recorded on existing obstetric EWS charts, anaesthetic charts and labour charts (partogram) will be entered on to the secure 4P study database for analysis. Examples of the intrapartum case report form are included in Appendix 3.

Other data collected: In addition, relevant medical and obstetric history will be extracted from the participants' notes to support analysis of the data. We will collect demographic information (age, height, weight, ethnicity, number of previous pregnancies, smoking status), medical and obstetric history, current health status, pregnancy-related health and current medications, at the initial assessment. At each follow up appointment we will collect assessments of smoking status, current health status, pregnancy-related health and current medications. The information will be collected onto standardized case report forms (for example, see Appendix 4). Where required, our definitions of variables included in the 4P study are detailed in Appendix 5.

At the discharge appointment, participants will be asked to fill out the standardised System Usability Survey (SUS)[23] to assess the usability of the 4P mobile system for home monitoring (Appendix 6).

Follow-up: In the antenatal period, the participants will be followed up at four to six weekly intervals. If they are co-enrolled in the Interbio-21st study, antenatal data will be collected by Interbio-21st staff. During the immediate postpartum period (days 0-14 after delivery), participants will be asked to undertake daily home monitoring. Additional postnatal data will be collected by research midwives on 2-3 occasions. If participants do not respond to contact by research midwives during the study, they will be treated as withdrawn after the third failed contact attempt. Data already collected from withdrawn participants will be included in the final analysis, unless explicitly requested otherwise. If participants complete the study they will receive a £20 gift voucher. A participant timeline for the study is shown in Figure 2.

Personnel: Trained research midwives will undertake all antenatal observation sets, and 1-3 postnatal observation sets, following study Standard Operating Procedures (SOPs) (for example, see Appendix 7). Research midwives will provide training in the use of home monitoring equipment at a suitable time around delivery, as described by SOPs. If abnormal vital signs are identified at the home visits, the research midwife will refer the participant to her usual midwife, GP or local maternity hospital assessment unit. All participants will be made aware that the information collected during the home-monitoring phase is not reviewed in real-time and that it remains their responsibility to seek assistance if they feel unwell. The

For peer review only - http://bmjopen.bmj.com/site/about/guidelines.xhtml

research midwives will extract all hospital data from the notes and observation charts into an electronic study database, following study SOPs. Research midwives will perform frequent site visits to carry out midwife training and address any recruitment and equipment issues.

Sample size: In order to determine the sample size we considered the precision of the data in estimating specific centiles. We assumed data are normally distributed at each gestational age. The standard error of the Pth centile is given using the standard formula[24]:

$$SE_P = SD\sqrt{\left(1+\frac{1}{2}Z_P^2\right)/n}$$

where SE is the standard error, SD is the standard deviation of the measurement of interest (which may change according to gestational age), Z_p is the value of the standard normal distribution corresponding to the Pth centile, and n is the sample size. To create an evidence based early warning score we desired a 95% confidence interval with a standard error of less than 0.10*SD at the boundaries. A sample size of 1000 women will achieve a standard error of 0.05*SD at the 2.5^{th} and 97.5^{th} centiles, and even greater precision at the less extreme centiles. Adequate precision is also met for any subgroup analysis; for example a sample size of 300 women will achieve a standard error of 0.1*SD at the 2.5^{th} and 97.5^{th} centiles. We have been conservative in these estimates as we have not considered the effect of serial measurements from the same women[25]. Using Royston's design factor of 2.3, a longitudinal study of 1000 women could have equivalent precision to a cross sectional study of around 2300 women[26].

Data Management: A custom designed website will be the main portal for manual data entry and vital sign review (Figure 3). Access will be restricted by username and 8 digit password to staff directly involved in the study. The 4P database and interfaces will be held within a secure virtual server protected with an NHS network firewall. For manual chart entry, automatic checks will be performed to ensure sequential chart entry and reasonable vital sign values.

Data quality: Duplicate measurements will be recorded for a subset of participant visits to document the effect of using only the first measurement (as in clinical practice). To minimise participant inconvenience, duplicate measurements will be reviewed by study statisticians to decide when an adequate number of duplicate measurements have been collected. Research midwives will undertake observation sets in the home to allow comparison between participant-taken and midwife-taken observations.

Temperature will be measured with both Bluetooth enabled and non-Bluetooth enabled devices to allow method comparison. Quality of the pulse oximeter recordings (heart rate and oxygen saturation) will be

For peer review only - http://bmjopen.bmj.com/site/about/guidelines.xhtml

assessed in real time by observation of the photoplethysmograph waveform (only visible to midwives). Additionally, the electronic heart rate measurement will be checked with a 60 second pulse palpation[27].

Vital sign data retrospectively extracted from patients' notes will be directly entered into the electronic case report form. The form automatically charts the vital signs onto electronic charts similar in appearance to the paper chart as the vital signs are entered, facilitating reliable data capture. Dual entry will be undertaken in 10% of cases to assess reliability.

Data analysis: Statistical methods to generate gestational age-specific centiles for maternal vital signs will closely follow those methods described and used in the INTERGROWTH-21st Project for foetal growth measurements[28]. Pre-defined exclusion criteria will be applied to make sure only those with a normal pregnancy are included in the analysis. We will explore different statistical methods in order to achieve the best fit to the data, whilst being as simple as possible. In brief, these methods will include: mean and SD using fractional polynomials[29]; Cole's lambda, mu and sigma (LMS) method[30]; the lambda, mu, sigma and Box-Cox t distribution (LMST) method[31]); the lambda, mu, sigma and Box-Cox power exponential (LMSP) method[32]; and multi-level models. To present curves, we will use different smoothing techniques including: fractional polynomial[32]; cubic splines[33], and penalised splines[34]. The INTERGROWTH-21st Project concluded that there was no evidence of a non-normal distribution and therefore no benefit of the more complex modelling techniques and opted to use the mean and SD method[28]. We therefore anticipate that values of each vital sign will be normally distributed at each stage of pregnancy, and that we will also be able to choose the simpler mean and SD method, as the best option. In this case, we will build the model in a multi-level framework, taking account of the correlation in repeated measures within participants.

Goodness of fit will be assessed by visual inspection of empirical centiles versus fitted centiles, quantile-quantile plots of the residuals, plots of residuals versus fitted values, and the distribution of fitted Z scores across gestational ages.

It is expected that some participants will become lost to follow-up or will have some missing measurements. These participants will still be included in the analysis, unless all data are missing. Missing data will be implicitly imputed using the multilevel modelling techniques under the missing at random assumption (MAR).

Centiles will be derived in the following pre-defined subgroups: BMI, maternal age, parity, smoking status, ethnicity; and will be visually compared to assess differences between subgroup strata. Sensitivity analyses will be performed to assess the robustness of the final model to the various assumptions made, including assumptions around missing data, and the choice of participants included in the analysis.

For peer review only - http://bmjopen.bmj.com/site/about/guidelines.xhtml

Study timelines: expected study timelines are shown (appendix 8).

ETHICS AND DISSEMINATION

Ethical considerations: Informed written consent will be sought for all participants and participants can withdraw from the study at any time. The study is non-invasive and presents no significant risk to participants. Data are managed in accordance with the Data Protection Act (1998).

Participants in the 4P study will be observed more frequently than they would be as per usual maternity care. However, the opportunity to meet with health professionals more frequently could be considered beneficial. During home visits, either antenatally or postnatally, the research midwife will refer participants to their usual care team if any abnormal vital sign values are recorded. Participants are asked to monitor their own vital signs at home. There is the possibility that some measurements are abnormal. If elevated blood pressure values are recorded, the software on the Android tablet advise study participants to contact their local maternity unit (a list of telephone numbers is provided). Participants are advised that the research team does not monitor the data sent from home and that they are responsible for acting on the software instructions whenever any abnormally high or low values of vital signs are recorded.

Dissemination: Results from the study will be disseminated through conferences and published in international peer-reviewed journals. Participants will be able to access results through a link on the OSPREA (Oxford Safer Pregnancy Alliance) website. Important protocol modifications will be communicated on the ISRCTN where the study is registered (No. 10838017). The data will be analysed by the University of Oxford (Institute of Biomedical Engineering) in the first instance. Anonymised data will be made available to other researchers on application.

CONCLUSIONS

An evidence-based early warning system will enable clinicians to identify more quickly the ill or deteriorating woman in a hospital setting at any time during pregnancy, delivery and the immediate post-partum period. Currently there are a number of EWS charts for pregnant women being used in the UK with different thresholds for alerts, none of which are evidence-based. The 4P study will enable a standardised evidence-based obstetric EWS chart to be developed from a database of vital signs for gestation of pregnancy, delivery and two weeks postpartum.

For peer review only

For peer review only - http://bmjopen.bmj.com/site/about/guidelines.xhtml

Funding: The 4P study is supported by the NIHR Biomedical Research Centre, Oxford. RP and LL acknowledge the support of the RCUK Digital Economy Programme grant number EP/G036861/1 (Oxford Centre for Doctoral Training in Healthcare Innovation). LL is also supported by the Clarendon Fund.

Study sponsor: University of Oxford. Contact Heather House, Joint Research Office, Block 60, Churchill Hospital, Old Road, Oxford, OX3 7LE. Email: ctrg@admin.ox.ac.uk.

Roles: Study design. Collection, management, analysis, interpretation, write-up and submission are the responsibility of the researchers. Neither sponsor nor funder have ultimate authority over any of these activities.

Competing interests: None.

Ethics approval: NRES committee South East Coast – Brighton and Sussex (Ref: 14/LO/1312)

Contributors: PW is the principal investigator. PW, LM, LL, LT, OG, JK and FK made substantial contributions to the conception and design of the project. SG provided advice on statistical methodology. JK, FK and CE are involved in data collection. OG, AT, DS, LL and RP are involved in software design and data analysis. FK wrote the first draft of the present manuscript. RP and PW reviewed and updated the draft. All authors read and approved the final manuscript.

Figure captions:

Figure 1: Equipment used for vital sign data collection

Figure 2: Timeline of follow up visits during the antenatal and postnatal periods.

Figure 3: Review of vital sign data for participant 0009 on the 4P website (example).

For peer review only

REFERENCES

1. Saving Mothers' Lives: Reviewing maternal deaths to make motherhood safer - 2003-2005. The Seventh Report of the Confidential Enquiries into Maternal Deaths in the United Kingdom. 2007.
2. Saving Mothers' Lives: Reviewing maternal deaths to make motherhood safer: 2006–2008. The Eighth Report of the Confidential Enquiries into Maternal Deaths in the United Kingdom. 2011.
3. Saving Lives, Improving Mothers' Care. Lessons learned to inform future maternity care from the UK and Ireland. Confidential Enquiries into Maternal Deaths and Morbidity 2009-2012
4. Swanton RD, Al-Rawi S, Wee MY. A national survey of obstetric early warning systems in the United Kingdom. *International Journal of Obstetric Anesthesia* 2009;18:253-7.
5. Smith GB, Prytherch DR, Meredith P, Schmidt PE, Featherstone PI. The ability of the National Early Warning Score (NEWS) to discriminate patients at risk of early cardiac arrest, unanticipated intensive care unit admission, and death. *Resuscitation* 2013;84:465-70.
6. Tarassenko L, Clifton DA, Pinsky MR, Hravnak MT, Woods JR, Watkinson PJ. Centile-based early warning scores derived from statistical distributions of vital signs. *Resuscitation* 2011;82:1013-8.
7. Macdonald-Wallis C, Lawlor DA, Fraser A, May M, Nelson SM, Tilling K. Blood pressure change in normotensive, gestational hypertensive, preeclamptic, and essential hypertensive pregnancies. *Hypertension* 2012;59:1241-8.
8. Magriples U, Boynton MH, Kershaw TS, Duffany KO, Rising SS, Ickovics JR. Blood pressure changes during pregnancy: impact of race, body mass index, and weight gain. *American Journal of Perinatology* 2013;30:415-24.
9. Mahendru AA, Everett TR, Wilkinson IB, Lees CC, McEniery CM. A longitudinal study of maternal cardiovascular function from preconception to the postpartum period. *Journal of Hypertension*, 2014;32(4):849-856. doi: 10.1097/HJH.0000000000000090
10. Gu H, Zhang S, Qiao Y, Luo Z, Zeng Y, Wang Q. A study of maternal hemodynamic change during healthy pregnancy and women with gestation hypertension. *Bio-Medical Materials & Engineering*. 2006;16(1):77-82.
11. van Oppen ACC, van der Tweel I, Alsback GPJ, Heetharr RM, Bruinse HW. A Longitudinal study of maternal hemodynamics during normal pregnancy. *Obstetrics and Gynecology*, 1996;88(1):40-46
12. Halligan A, O'Brien E, O'Malley K, Mee F, Atkins N, Conroy R, Walshe JJ, Darling M. Twenty-four-hour ambulatory blood pressure measurement in a primigravid population. *Journal of Hypertension* 1993;11:869-873
13. Blake MJ, Martin A, Manktelow BN, Armstrong C, Halligan AW, Panerai RB, et al. Changes in baroreceptor sensitivity for heart rate during normotensive pregnancy and the puerperium. *Clinical Science* 2000;98(3):259-68.
14. Grindheim G, Estensen ME, Langesaeter E, Rosseland LA, Toska K. Changes in blood pressure during healthy pregnancy: a longitudinal cohort study. *Journal of Hypertension* 2012;30(2):342-50.
15. Clapp JF, Capeless E. Cardiovascular Function before, during and after the first and subsequent pregnancies. *The American Journal of Cardiology* 1997;80:1469-1473.
16. Tuffnell DJ, Buchan PC, Albert D, Tyndale-Biscoe S. Fetal heart rate responses to maternal exercise, increased maternal temperature and maternal circadian variation. *Journal of Obstetrics and Gynaecology* 1990;10(5):387-91.
17. Templeton A, Kelman GR. Maternal blood-gasses, physiological shunt and VD/VT in normal pregnancy. *British Journal of Anaethesiology* 1976;48:1001-1004
18. Ekholm EMK, Erkkola RU, Piha SJ, Jaolnen JO, Metsala TH, Antila KJ. Changes in autonomic cardiovascular control in mid-pregnancy. *Clinical Physiology* 1992;12:527-536
19. Deckhardt R, Fembacher PM, Schneider KTM, Graeff H. Maternal arterial oxygen saturation during labour and delivery: Pain-dependent alterations and effects on the newborn. Obstetrics and Gynecology. 1987;70(1):21-5.
20. King S, Bannister PR, Clifton DA, Tarassenko L. Probabilistic Approaches to Condition Monitoring of Aerospace Engines. *Journal of Aerospace Engineering* 2009;223:533-41.
21. INTERBIO-21st, 2016. Retreived 15-06-2016 from www.interbio21.org.uk

For peer review only

For peer review only - http://bmjopen.bmj.com/site/about/guidelines.xhtml

22. Papageorghiou AT, Kennedy SH, Salomon LJ, Ohuma EO, Cheikh Ismail L, Barros FC, Lambert A, Carvalho M, Jaffer YA, Bertino E, Gravett MG, Altman DG, Purwar M, Noble JA, Pang R, Victoria CG, Bhutta ZA, Villar J. International standards for early fetal size and pregnancy dating based on ultrasound measurement of crown-rump length in the first trimester of pregnancy. *Ultrasound Obstetrics and Gynecology* 2014;44(6):641-648
23. Brooke. SUS – A quick and dirty usability scale. *Usability evaluation in industry* 1996;189(194):4-7
24. Healy MJ. Notes on the statistics of growth standards. *Annals of Human Biology* 1974;1(1):41-7
25. Altman DG, Ohuma EO. Statistical considerations for the development of prescriptive fetal and newborn growth standards in the INTERGROWTH-21st Project. *British Journal of Obstetrics and Gynaecology* 2013;120 Suppl 2:71-6
26. Royston P. Calculation of unconditional and conditional reference intervals for foetal size and growth from longitudinal measurements. *Statistics in Medicine* 1995;14(13):1417-36.
27. Royal College od Nursing. Standards for assessing, measuring and monitoring vital signs in infants, children and young people. Retrieved 12-09-2016 from: https://www2.rcn.org.uk/__data/assets/pdf_file/0004/114484/003196.pdf
28. Papageorghiou AT, Ohuma EO, Altman DG et al International standards for fetal growth based on serial ultrasound measurements: the Fetal Growth Longitudinal Study of the INTERGROWTH-21st Project. *Lancet* 2014;384:869-79
29. Royston P, Altman DG. Regression using fractional polynomials of continuous covariates: parsimonious parametric modelling. *Journal of the Royal Statistical Society* 1994;43:429–67.
30. Cole TJ, Green PJ. Smoothing reference centile curves: the LMS method and penalized likelihood. *Statistics in Medicine* 1992;11:1305–19.
31. Rigby RA, Stasinopoulos DM. Using the Box-Cox *t* distribution in GAMLSS to model skewness and kurtosis. *Statiscial Modelling* 2006;6**:** 209–29.
32. Rigby RA, Stasinopoulos DM. Smooth centile curves for skew and kurtotic data modelled using the Box–Cox power exponential distribution. *Statistics in Medicine* 2004;23:3053–76.
33. Green PJ, Silverman BW. Nonparametric regression and generalized linear models: a roughness penalty approach. London:Chapman & Hall, 1994.
34. Eilers PHC, Marx BD. Flexible smoothing with B-splines and penalties. *Statistical Science* 1996;11:89–158.

For peer review only

For peer review only - http://bmjopen.bmj.com/site/about/guidelines.xhtml

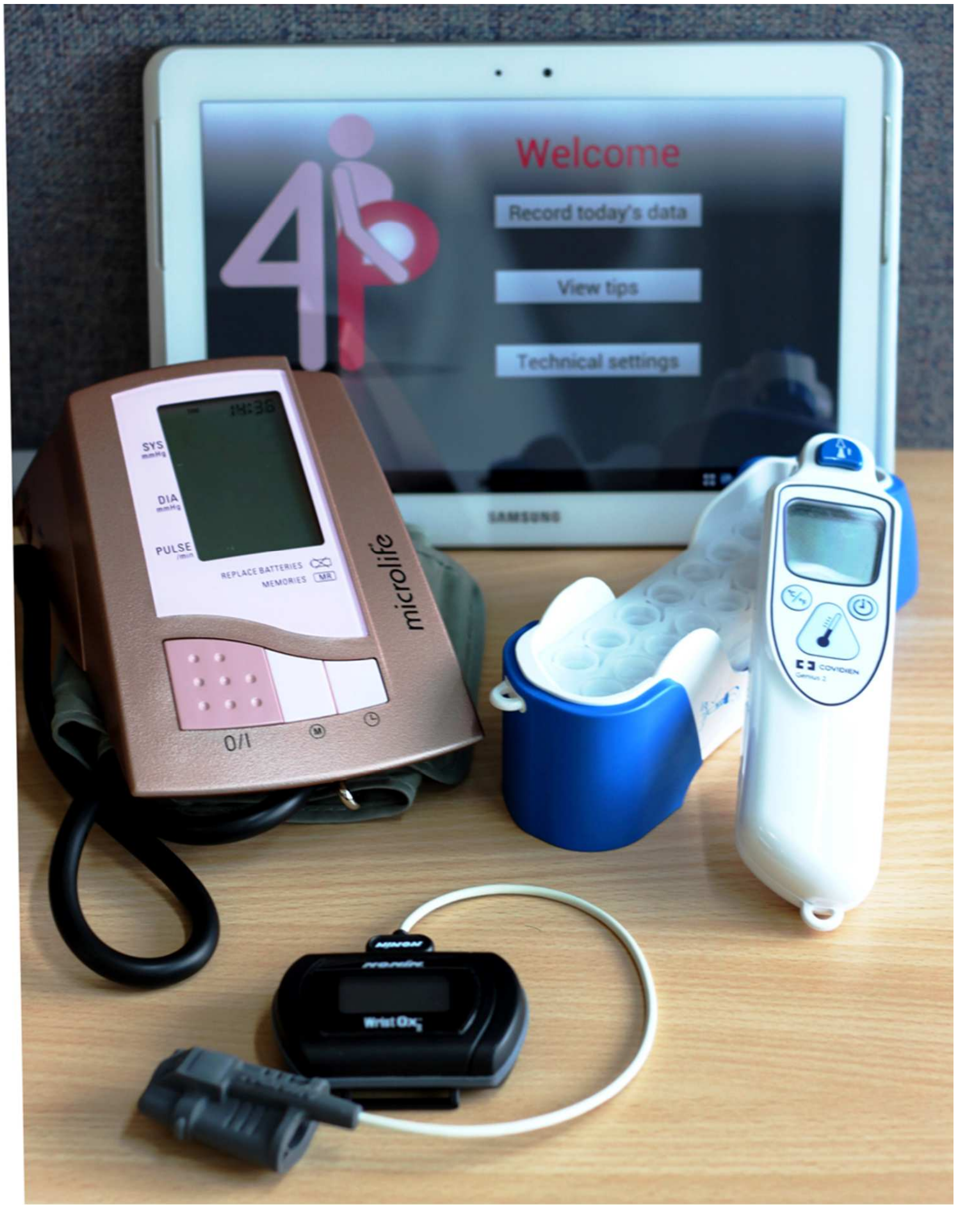

Equipment used for vital sign data collection

92x117mm (300 x 300 DPI)

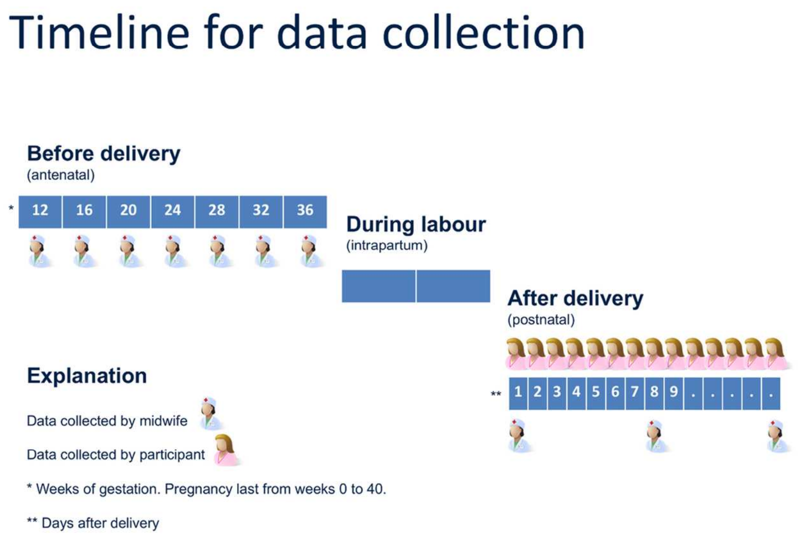

Timeline of follow up visits during the antenatal and postnatal periods.

121x85mm (300 x 300 DPI)

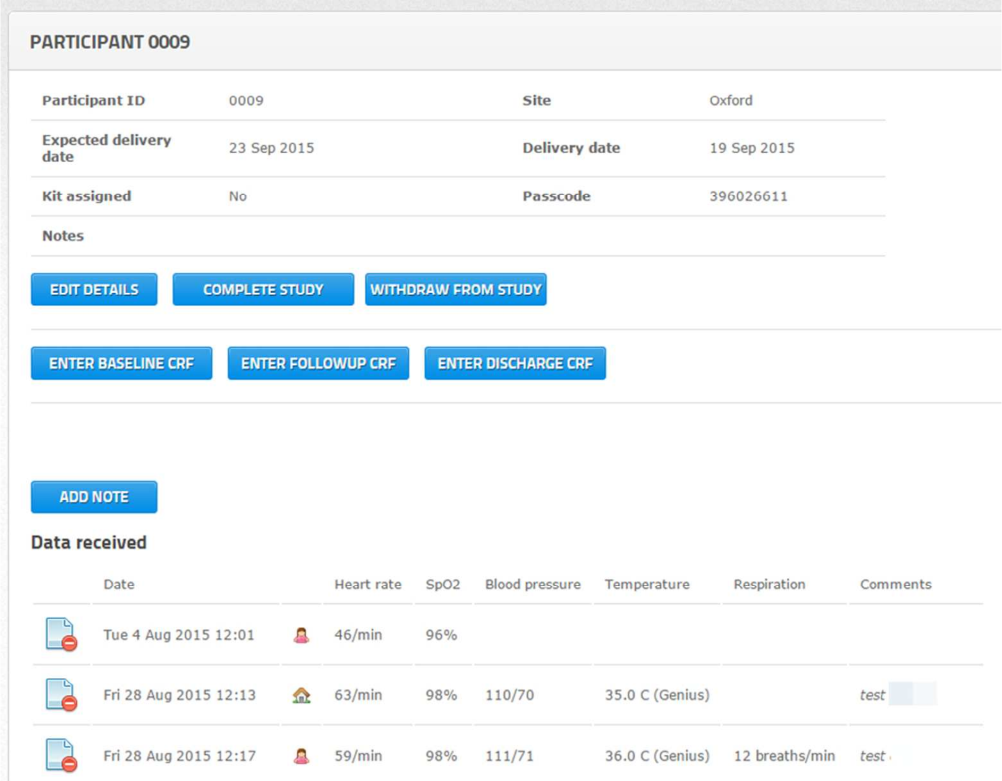

Review of vital sign data for participant 0009 on the 4P website (example).

87x67mm (300 x 300 DPI)

APPENDICES

Appendix 1: Sites in study

John Radcliffe Hospital, Oxford University Hospitals NHS Foundation Trust, UK

Royal Victoria Infirmary, The Newcastle Upon Tyne Hospitals NHS Foundation Trust, UK

St Thomas' Hospital, Guy's and St Thomas' NHS Foundation Trust, UK

Appendix 2: Inclusion/Exclusion criteria

Inclusion Criteria

a) ≥16 years of age (parental consent to be obtained for participants <18 years old)

b) BMI < 35 kg/m2

c) Ultrasound dating scan < 14 weeks

d) Naturally conceived pregnancy

e) Singleton pregnancy

f) Pregnant with an intent to deliver in hospital

g) Able to understand written or spoken English or has their own interpreter willing to translate

h) Willing and able to use the home monitoring equipment

i) Within category 1 of the American Society of Anaesthesiologists' classification of physical status at enrolment ("A normal healthy patient without any clinically important co-morbidity and without a clinically significant past/present medical history"). Women with the following conditions would be included in the 4P study, under the above definition:

- Obesity
- Smoking
- Diabetes
- Renal disease without hypertension
- High BP at recruitment (defined as SBP > 140mmHg and DBP > 90mmHg, not previously diagnosed with hypertension)
- Gestational hypertension or pre-eclampsia diagnosed in a previous pregnancy (even if treated, as long as essential hypertension was not developed)

Exclusion Criteria

Any known medical condition expected by the recruiting clinician to alter maternal vital signs, such as:

- Hypertension (defined as SBP > 140mmHg and DBP > 90mmHg), ever requiring treatment
- Congenital cardiac disease
- Ischaemic cardiac disease

For peer review only

- Bronchiectasis
- Fibrotic lung disease
- Malignant disease

Appendix 3: Intrapartum case report form (electronic)

Intrapartum data is transcribed from patient notes through electronic case report forms. Screenshots of these intrapartum forms are presented in the following four figures.

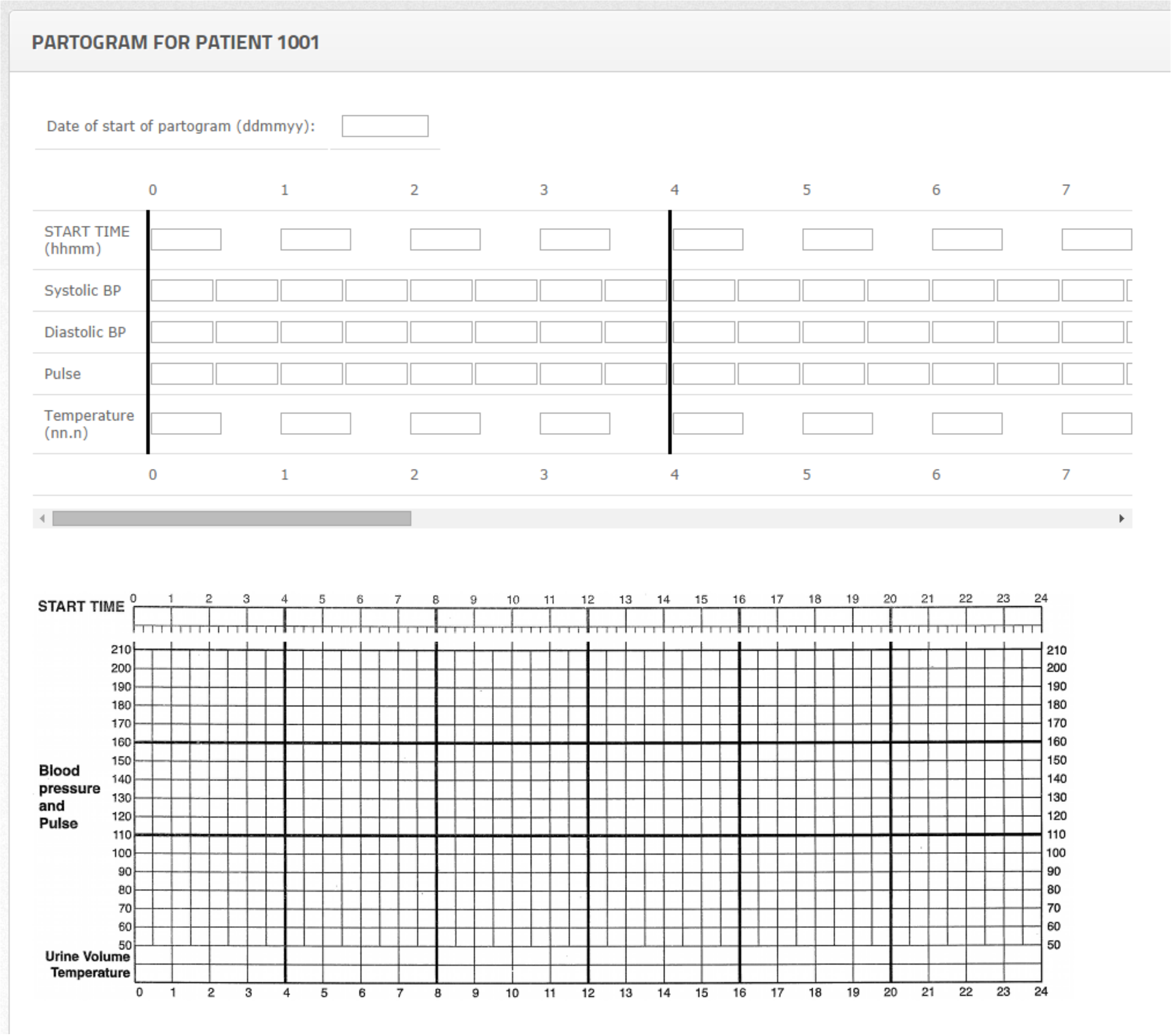

Partogram chart

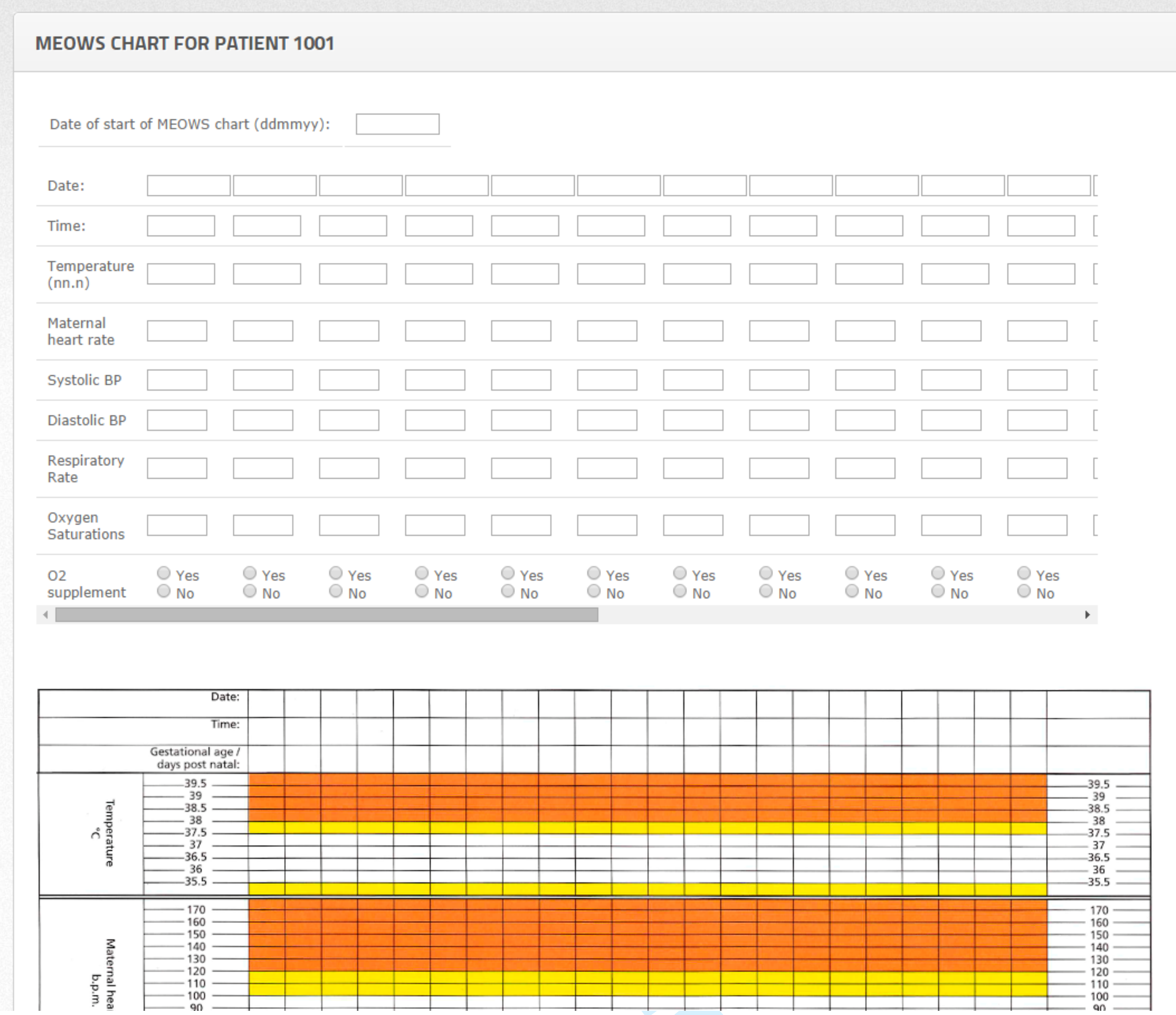

Maternal early warning score chart (referred to as a MEOWS chart in OUHs).

For peer review only

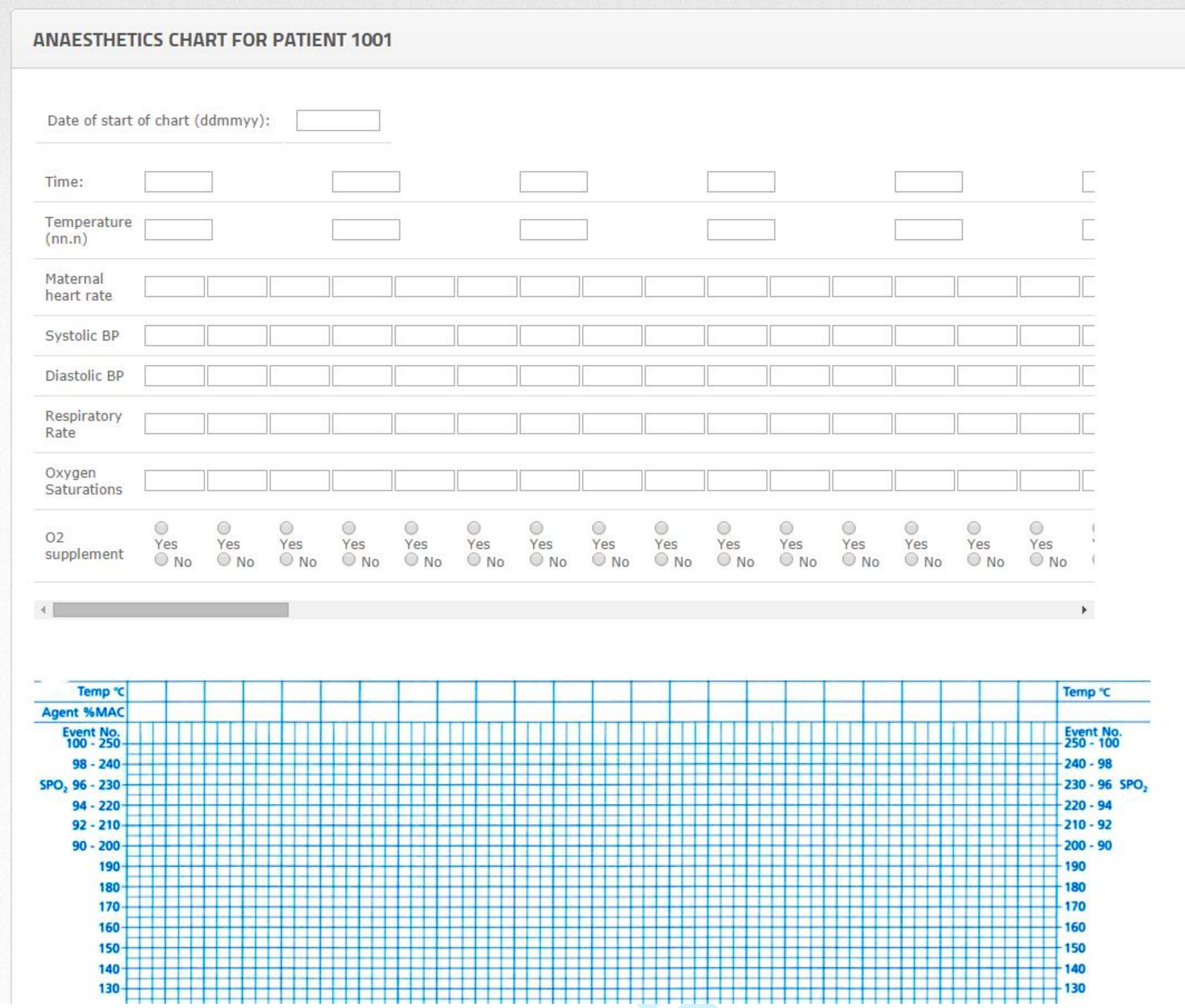

Anaesthetics chart

ew only

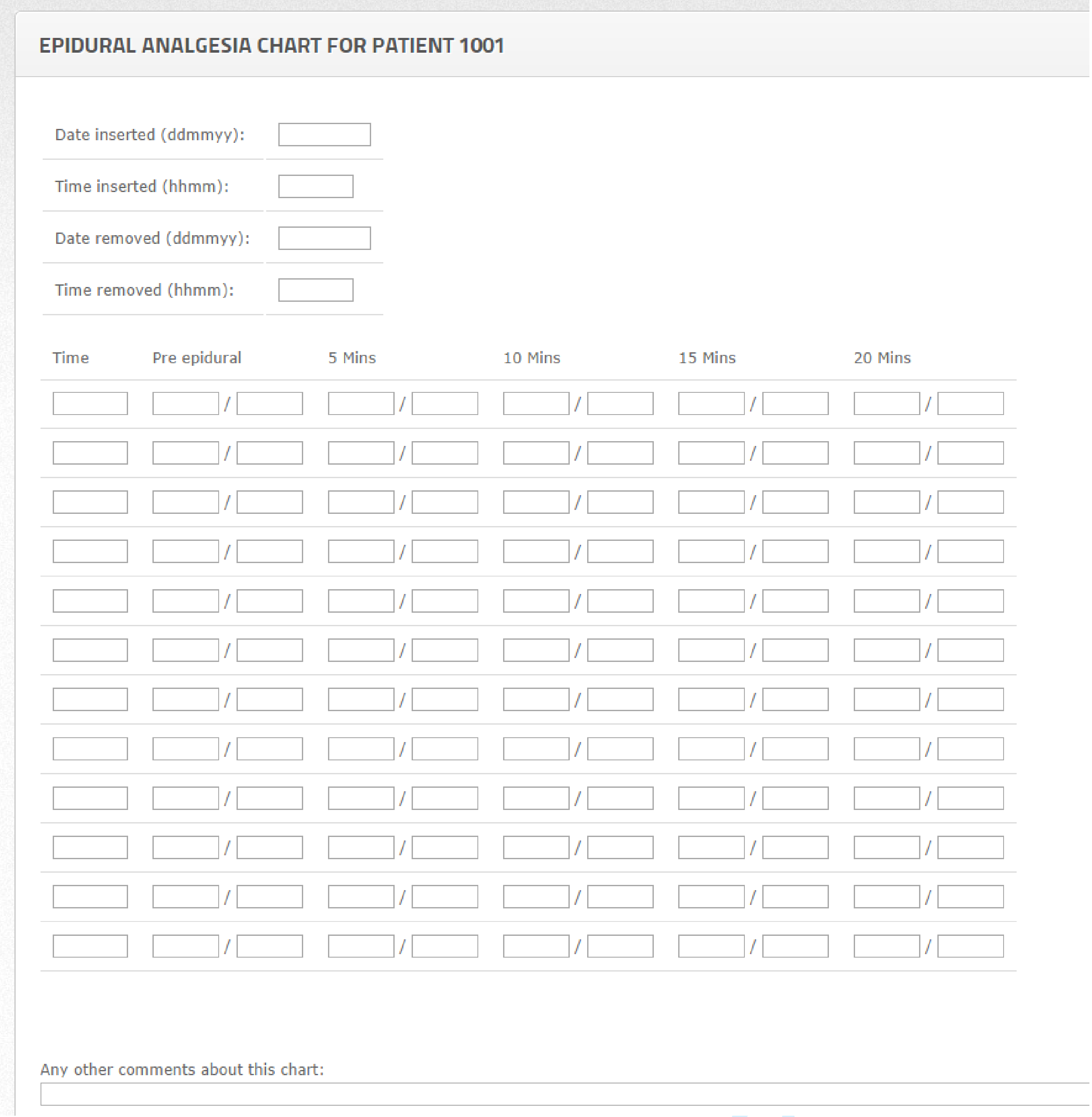

EPIDURAL ANALGESIA CHART FOR PATIENT 1001

Date inserted (ddmmyy):	
Time inserted (hhmm):	
Date removed (ddmmyy):	
Time removed (hhmm):	

Time	Pre epidural	5 Mins	10 Mins	15 Mins	20 Mins
	/	/	/	/	/
	/	/	/	/	/
	/	/	/	/	/
	/	/	/	/	/
	/	/	/	/	/
	/	/	/	/	/
	/	/	/	/	/
	/	/	/	/	/
	/	/	/	/	/
	/	/	/	/	/
	/	/	/	/	/
	/	/	/	/	/

Any other comments about this chart:

Epidural chart

only

Appendix 4: Case Report Forms

Supporting information are extracted from the participant's notes at each appointment. For example, the form used to collect demographic information, medical and obstetric history, current health status and pregnancy-related health and current medications at the initial baseline assessment, is shown below.

(1) BASELINE INFORMATION

Participant ID:	___ ___ ___ ___
Date:	___ ___ - ___ ___ - ___ ___ ___ ___ (D D M M Y Y Y Y)

SECTION 1: DEMOGRAPHIC INFORMATION

**1.1** Date of Birth	___ ___ - ___ ___ - ___ ___ ___ ___	**1.7** Haemoglobin Level	___ ___ . ___ g/dl
**1.2** Height	___ ___ ___ . ___ cm	**1.8** OR Haematocrit	___ ___ . ___ %
**1.3** Weight	___ ___ ___ . ___ kg	**1.9** Date of blood test:	___ ___ - ___ ___ - ___ ___ ___ ___
**1.4** In the last three months, has she smoked?	☐ Yes ☐ No	**1.10** Proteinuria (by dipstick)	☐ 0/trace ☐ + ☐ ++
**1.5** If yes, how many cigarettes/cigars per day?	___ ___	☐ +++ ☐ ++++	☐ No urine test available
**1.6** Has she lived in the same household as someone who smokes?	☐ Yes ☐ No	**1.11** Date of urine test:	___ ___ - ___ ___ - ___ ___ ___ ___

**1.12** Ethnicity	White	Asian or Asian British	Black or Black British
	☐ A : White - British	☐ H : Indian	☐ M : Black - Caribbean
	☐ B : White - Irish	☐ J : Pakistani	☐ N : Black - African
	☐ C : Any other White background	☐ K : Bangladeshi	☐ P : Any other Black background
	Mixed	☐ L : Any other Asian background	Other ethnic group
	☐ D : Mixed - White and Black Caribbean		☐ R : Chinese
	☐ E : Mixed - White and Black African		☐ S : Any other Ethnic Group
	☐ F : Mixed - White and Asian		☐ Z : Does not want to give Ethnic Group
	☐ G : Any other mixed background		

SECTION 2: MEDICAL HISTORY

Has she ever been diagnosed with or treated for any of the following medical conditions?

**2.1** Diabetes	☐ Yes ☐ No	**2.9** Hepatic disease	☐ Yes ☐ No
**2.2** Thyroid disease	☐ Yes ☐ No	**2.10** Gastrointestinal disease	☐ Yes ☐ No
**2.3** Other endocrinological condition	☐ Yes ☐ No	**2.11** Autoimmune conditions	☐ Yes ☐ No
**2.4** Any type of malignancy/cancer (including leukaemia or lymphoma)	☐ Yes ☐ No	**2.12** Haematological conditions	☐ Yes ☐ No
**2.5** Cardiac disease	☐ Yes ☐ No	**2.13** Venous thromboembolism (VTE)	☐ Yes ☐ No
**2.6** Hypertension without treatment	☐ Yes ☐ No	**2.14** Any congenital abnormality or genetic disease	☐ Yes ☐ No
**2.7** A chronic respiratory disease (including chronic asthma)	☐ Yes ☐ No	If yes, specify: ______________________________	
**2.8** Proteinuria, kidney disease or chronic renal disease	☐ Yes ☐ No	**2.15** Any other clinically relevant condition	☐ Yes ☐ No
		If yes, specify: ______________________________	

(Continued on next page)

For peer review only - http://bmjopen.bmj.com/site/about/guidelines.xhtml

SECTION 3: OBSTETRIC AND GYNAECOLOGICAL HISTORY

3.1 Date of first day of last menstrual period (LMP) ___ ___ - ___ ___ - ___ ___ ___ ___ (D D M M Y Y Y Y)

3.2 Number of previous pregnancies, excluding this pregnancy ___ ___

3.3 Number of previous births ___ ___

SECTION 4: CURRENT PREGNANCY-RELATED HEALTH

4.1 CRL (Crown Rump Length) measurement: ___ ___ ___ . ___ mm

4.2 Date of CRL measurement: ___ ___ - ___ ___ - ___ ___ ___ ___ (D D M M Y Y Y Y)

During this pregnancy, has she been diagnosed with or treated for any of the following conditions?

4.3 Severe vomiting requiring hospitalisation ☐ Yes ☐ No

4.5 Anaemia ☐ Yes ☐ No

4.4 Hypertension ☐ Yes ☐ No

4.6 Infection/febrile illness ☐ Yes ☐ No

SECTION 5: CURRENT MEDICATIONS

Is she routinely taking any of the following medication?

5.1 Aspirin ☐ Yes ☐ No

5.2 Non-steroidal anti-inflammatories ☐ Yes ☐ No

5.3 Insulin ☐ Yes ☐ No

5.4 Antihypertensives ☐ Yes ☐ No

Which antihypertensives? ☐ ACE inhibitors ☐ Beta-blockers ☐ Calcium channel blockers

5.5 Any other antibiotics/antivirals ☐ Yes ☐ No

If yes, specify: ______________________

5.6 Any other treatment ☐ Yes ☐ No

If yes, specify: ______________________

For peer review only - http://bmjopen.bmj.com/site/about/guidelines.xhtml

Appendix 5: Definitions

Definitions of variables collected in the 4P study, mostly taken from the National Institute for Health and Care Excellence (NICE) guidelines and quality standards.

**Baseline information**	
Ethnicity	Categories from the 2011 UK Census<br>A White - British<br>B White - Irish<br>C Any other White background<br>D Mixed - White and Black Caribbean<br>E Mixed - White and Black African<br>F Mixed - White and Asian<br>G Any other mixed background<br>H Indian<br>J Pakistani<br>K Bangladeshi<br>L Any other Asian background<br>M Black - Caribbean<br>N Black - African<br>P Any other Black background<br>R Chinese<br>S Any other Ethnic Group<br>Z Does not want to give Ethnic Group
Weight	Measured in kilograms (kg) to 1 decimal place.
Height	Measured in centimetres (cm) to 1 decimal place.
Crown Rump Length	Ultrasound measurement of crown rump length in millimetres (mm) to 1 decimal place.
Gestational age	Calculated from Crown Rump Length to the nearest day, using the algorithm developed by the Interbio study [1].
Smoker	Has smoked in the three months before recruitment.
Proteinuria	Presence of proteinuria in the urine, measured in milligrams/decilitre (mg/dl) to 1 decimal place.
**Medical Conditions**	
Diabetes	Glucose intolerance. Any type of diabetes is recorded.
Thyroid disease	A medical condition impairing the function of the thyroid. For example: hypo- or hyper-thyroidism, arathyroidism (PTH).

For peer review only

Other endocrinological condition	Diseases related to the endocrine glands of the body. For example: Addison's disease, adrenal gland disorders, hypophysitis
Malignancy/cancer	A malignant tumour, where cells proliferate out of control and inhibit the normal function of an organ system. Any type of cancer recorded, including leukaemia or lymphoma.
Cardiac disease	Disease of the heart or blood vessels. For example: arrhythmias, murmurs, valve diseases, atherosclerosis, atrial fibrillation, sarcoma, pericarditis, or cardiomyopathy.
Respiratory disease	Diseases of the airways and other parts of the lung. For example: asthma, chronic obstructive pulmonary disease (COPD), lung cancer, cystic fibrosis, sleep apnoea or occupational lung diseases.
Pyelonephritis or kidney disease	Disease of the kidneys. For example: chronic kidney disease, polycystic kidney disease, or kidney failure.
Hepatic disease	Disease of the liver. For example: hepatitis, haemochromatosis, or alcohol-related liver disease.
Gastrointestinal disease	Diseases involving the gastrointestinal tract (oesophagus, stomach, small intestine, large intestine and rectum), and the accessory organs of digestion (liver, gallbladder, and pancreas). For example: Crohn's disease, coeliac disease, ulcerative colitis or any severe malabsorption condition (requiring special diet)
Autoimmune conditions	The body's immune system attacks and destroys healthy body tissue by mistake. There are more than 80 types of autoimmune disorders. For example: Addison's disease, multiple sclerosis, rheumatoid arthritis.
Haematological conditions	Disorder of the blood. For example: anaemia, bleeding disorders such as haemophilia, blood clots, and blood cancers such as leukaemia, lymphoma, and myeloma.
Venous thromboembolism	A blood clot in a vein.
Infection	The invasion and multiplication of microorganisms such as bacteria, viruses, and parasites that are not normally present within the body.
Febrile illness	A nonspecific term for any illness of sudden onset accompanied by fever.
**Pregnancy complications**	
Gestational diabetes	Any degree of glucose intolerance with onset or first recognition during pregnancy.
Gestational hypertension	Blood pressure is ≥ 140/90mmHg after 20 weeks gestation in a previously normotensive pregnancy [2]
Preeclampsia	Gestational hypertension with the presence of proteinuria (excessive protein substance, chiefly albumin, in the urine)

For peer review only

Severe preeclampsia	Blood pressure is ≥ 160/110mmHg on two occasions, between 4 and 168 hours apart, or if the first measurement was immediately followed by treatment with an antihypertensive, either of these scenarios being associated with the presence of proteinuria.
Eclampsia	The occurrence of convulsions and/or coma unrelated to her cerebral conditions in a woman with signs and symptoms of pre-eclampsia. Seizures are of grand mal type and may first appear before labour, during labour, or up to 48 hours postpartum.
HELLP syndrome	A group of symptoms that occur in pregnant women who have pre-eclampsia or eclampsia and who also show signs of liver damage and abnormalities in blood clotting. It is characterised by: **H**aemolysis, **EL** (elevated) liver enzymes and **LP** (low platelet) count.
Primary postpartum haemorrhage	Blood loss from the genital tract >500mls (vaginal birth) or >1000mls (caesarean birth) within 24 hours of the birth of a baby. [3]
Secondary postpartum haemorrhage	Excessive or abnormal bleeding from the genital tract between 24 hours and 6 weeks in the postnatal period. [4]
Anaemia	Iron deficiency. Anaemia in pregnancy is defined as first trimester haemoglobin (Hb) less than 110 g/l, second/third trimester Hb less than 105 g/l, and postpartum Hb less than 100 g/l, in line with British Committee for Standards in Haematology (BCSH) guidance. [5]
**Delivery and labour**	
Spontaneous vaginal delivery	Spontaneous initiation of labour and delivery with no intervention.
Vaginal assisted	Delivery assisted with forceps or vacuum. Vacuum extraction uses a suction cup that is placed over the baby's head, which allows the physician to pull the child through the birth canal.
Caesarean section	An operative delivery of baby through a small insertion to the mother's abdomen. Including elective (planned, performed at or after 39 weeks), and emergency (unplanned) caesarean sections.
Assisted breech or breech extraction	The baby is born feet-first or buttocks first and requires assistance.
Preterm delivery	Delivery before 37 weeks.
Preterm pre-labour rupture of membranes (PPROM)	Spontaneous rupture of the membranes at less than 37 weeks gestation and prior to onset of labour.
Foetal distress	Abnormal foetal heart rate (FHR) or biophysical profile (BPP) (as a reason for labour being induced or caesarean section performed)

For peer review only

References for Appendix 5:

1. Papageorghiou AT, Kennedy SH, Salomon LJ, Ohuma EO, Chiekh Ismail L, Barros FC, Lambert A, Carvalho, M, Jaffer YA, Bertino E, Gravett MG, Altman DG, Purwar M, Noble JA, Pang R, Victoria CG, Bhutta ZA, Villar J. International standards for early fetal size and pregnancy dating based on ultrasound measurement of crown-rump length in the first trimester of pregnancy. *Ultrasound in Obstetrics and Gynecology* Published Online First: 2 November 2014. doi: 10.1002/uog.13448.
2. National Institute for Health and Care Excellence (NICE). Hypertension in pregnancy: diagnosis and management (Clinical guideline 107). 2010. Retrieved 01-08-2016 from: nice.org.uk/guidance/cg107
3. Mousa HA, Alfirevic Z. Treatment for primary postpartum haemorrhage. *Cochrane Database of Systematic Reviews* Published First Online 13 February 2014. doi: 10.1002/14651858.CD003249.pub3
4. Alexander J,Thomas PW, Sanghera J. Treatments for secondary postpartum haemorrhage. *Cochrane Database of Systematic Reviews* Published Online First: 21 January 2002. doi: 10.1002/14651858.CD002867
5. Royal College of Obstetricians and Gynaecologists (RCOG). Blood transfusion in obstetrics (Green-top guideline 47). 2015. Retrieved 01-08-2016 from: https://www.rcog.org.uk/globalassets/documents/guidelines/gtg-47.pdf

For peer review only

Appendix 6: Usability survey

Oxford University Hospitals NHS
NHS Trust

UNIVERSITY OF OXFORD

The 4P Participants' Usability Survey

Please answer each question 1-10 by placing a cross (X) on the scale to indicate your experience of using the 4P m-Health system.	*Strongly disagree 0*	*1*	*2*	*3*	*Strongly agree 4*
1. I think I would like to use the 4P system frequently	☐	☐	☐	☐	☐
2. I find the 4P system unnecessarily complex	☐	☐	☐	☐	☐
3. I think the 4P system is easy to use	☐	☐	☐	☐	☐
4. I think that I would need the ongoing support of a technical person or research midwife to be able to use the 4P system	☐	☐	☐	☐	☐
5. I find that the various functions within the 4P system are well integrated	☐	☐	☐	☐	☐
6. I think that there is too much inconsistency in the design of the 4P system	☐	☐	☐	☐	☐
7. I would imagine that most people would learn to use the 4P system very quickly	☐	☐	☐	☐	☐
8. I find the 4P system very cumbersome to use	☐	☐	☐	☐	☐
9. I feel very confident using the 4P system	☐	☐	☐	☐	☐
10. I needed to learn a lot of things before I could competently use the 4P system	☐	☐	☐	☐	☐
Additional feedback or comments (optional):					

Thank you for taking the time to complete this questionnaire

Appendix 7: Standard Operating Procedures (SOPs)

Three examples of SOPs used in the 4P study are presented in the following pages

- 001 How participants are taught to use the correct use of all the equipment
- 016 Extraction of patient notes onto the database
- 017 Measurement of each vital sign by midwives

Appendix 8: Timeline

- 1st August 2012: Stage 1 of 4P study commences (as a sub-study of the Interbio-21st study, amendment approval 6th June 2012).
- 8th July 2015: Stage 2 of 4P study commences (Ethical approval 1st May 2015)
- 31st December 2016: full recruitment.
- Expected study completion (last vital sign data set submitted) September 2017

For peer review only

STANDARD OPERATING PROCEDURE

Nuffield Department of Obstetrics and Gynaecology

SOP No: 001-V1

SOP Title: Teaching Kit Use To 4P Participants

SOP Number 001-V1

SOP Title Teaching Kit Use To 4P Participants

	NAME	TITLE	SIGNATURE	DATE
**Author**	**Jude Kemp**	**Research Midwife**		**27/02/2013**
**Reviewer**				
**Authoriser**				

**Effective Date:**	
**Review Date:**	

READ BY			
**NAME**	**TITLE**	**SIGNATURE**	**DATE**

Adapted from CTRG Template SOP Version 2.1
© Copyright: The University of Oxford 2009

For peer review only - http://bmjopen.bmj.com/site/about/guidelines.xhtml

STANDARD OPERATING PROCEDURE

Nuffield Department of Obstetrics and Gynaecology

SOP No: 001-V1

SOP Title: Teaching Kit Use To 4P Participants

1. PURPOSE

The purpose of this SOP is to describe a standardised method of teaching the use of the 4P home monitoring equipment.

Training all participants to the same standard will help eliminate user error as much as possible and ensure a high quality data set.

2. INTRODUCTION

The 4P study aims to capture 14 days' worth of physiological readings from participating women using a standardised home monitoring equipment kit. The readings consist of resting pulse oximetry, resting blood pressure, pulse and temperature.

The readings are collected onto a tablet computer by the participant and transmitted to the study database (hosted on a secure NHS server) at the end of each recording session. At the end of the 14 days, any un-submitted data are retrieved by 4P engineers and added to the study database for analysis. The data will be used to devise an obstetric early warning system.

It is important to ensure that the 4P participants are confident in using their home monitoring equipment before the start of the home monitoring phase.

The ideal teaching opportunity is at the participant's final two INTERBIO visits at approximately 36 and 38 weeks gestation.

If this is not possible, or the participant delivers prior to these appointments, the teaching should take place prior to discharge from hospital.

3. SCOPE

This SOP applies only to the 4P research midwives teaching the use of home monitoring equipment.

4. DEFINITIONS

The 4P home monitoring equipment consists of the following items:

- Tablet computer (Samsung Galaxy Tab) with charger and charging lead.
- Pulse oximeter (Nonin 3150 WristOx) with finger sensor (To ease understanding, it will be referred to as a finger meter)
- Thermometer (Genius 2) with a supply of lens caps (36 lens caps per unit)
- Blood pressure monitor (Microlife 3BTO-A(2)) with appropriate size cuff.
- 4P study instruction manual for parents describing set up of equipment.

Adapted from CTRG Template SOP Version 2.1
© Copyright: The University of Oxford 2009

For peer review only - http://bmjopen.bmj.com/site/about/guidelines.xhtml

5. RESPONSIBILITIES

The 4P midwife is responsible for teaching the correct use of the home monitoring equipment and ensuring the participant feels confident in its use.

6. SPECIFIC PROCEDURE

6.1 Before teaching the use of the home monitoring equipment, the 4P midwife should confirm that the 4P participant is still willing to take part in the 4P study. Where possible, arrange for the participant's partner or relative to be present and teach him/her the use of the equipment too.

6.2 Teaching should take place in a private room if possible to provide a calm environment for learning and asking questions. If a private room is not available (e.g. on a ward) the curtains should be drawn around the bed to provide privacy.

6.3 The 4 P midwife must explain the rationale behind the data collection and give the participant an opportunity to ask questions.

6.4 The 4P midwife will teach the use of the following:

a. Pulse oximeter

b. blood pressure cuff positioning and measurement

c. thermometer use

To ensure the participant has rested prior to the blood pressure reading, it is important that the recordings are always carried out in the following order:

Pulse Oximetry, Blood Pressure, Temperature

Adapted from CTRG Template SOP Version 2.1
© Copyright: The University of Oxford 2009

For peer review only - http://bmjopen.bmj.com/site/about/guidelines.xhtml

a. Demonstration of correct pulse oximeter use:

Tell the participant:

It is important that the following advice is always followed in order to obtain a correct reading.

Ask the participant to

- Switch on the Nonin Pulse oximeter and place the grey Nonin pulse oximeter finger meter over the index finger of the left hand with the cable side upper most.
- Place the oximeter on a flat surface and sit comfortably while it is recording.
- Refrain from speaking during the reading.

The pulse oximeter will not work if the participant is wearing false nails or dark nail varnish. Ask the participant to remove nail varnish. If she is wearing or intends to wear false nails, an ear clip will be supplied.

b. Demonstration of correct blood pressure cuff positioning

The appropriate cuff size should be used:

Standard cuff bladder 12-26 cm for the majority of adult arms (arm circumference 33-50 cm)

Large cuff bladder 12-40 cm for obese arms (arm circumference > 50 cm)

Small cuff bladder 12-18 cm for lean adult arms (arm circumference 23-33 cm)

(Combined European Society/Nice/British Hypertension Society recommendations, 2012) http://www.bhsoc.org/frequently-asked-questions/#BPM4

Adapted from CTRG Template SOP Version 2.1
© Copyright: The University of Oxford 2009

For peer review only - http://bmjopen.bmj.com/site/about/guidelines.xhtml

Tell the participant:

It is important that the following advice is always followed in order to obtain a correct reading.

Ask the participant to

- Sit with her arm stretched out and supported, for example on the arm of a chair or a cushion.
- The feet should be flat on the floor.
- Sit quietly and relax.
- Wear clothing with a loose sleeve as she will need to push it up. If the sleeve is tight, ask the participant to slip her arm out of the sleeve.
- Do not push tight sleeves up, as she will not be able to position the cuff correctly.
- Slip her arm into the cuff with the black tubing at the front and move it up the arm, 3cm above the elbow.
- Fasten the Velcro and adjust the position of the cuff if needed.
- Press the 'on' button. Explain that the cuff will go tight, which may be slightly uncomfortable.
- Encourage the participant not to talk during the measurement.
- Enter the BP value on the tablet computer.

IMPORTANT

- Explain that a high BP reading will cause a message to appear onscreen, asking her to repeat the measurement in half an hour.
- If the measurement is still high, a message will appear, asking her to access medical help via the Maternity Assessment Unit or Day Assessment Unit at the John Radcliffe Hospital or the Horton Hospital in Banbury. (see SOP 005 for details)
- Explain to the participant that it is her responsibility to access medical assistance and that the data she is recording are NOT monitored by a doctor or midwife.

Adapted from CTRG Template SOP Version 2.1
© Copyright: The University of Oxford 2009

For peer review only - http://bmjopen.bmj.com/site/about/guidelines.xhtml

c. Demonstration of correct use of the thermometer

Tell the participant:

It is very important that the following advice is always followed in order to obtain a correct temperature reading:

Ask the participant to:

- Lift the thermometer out of its cradle. See photo.

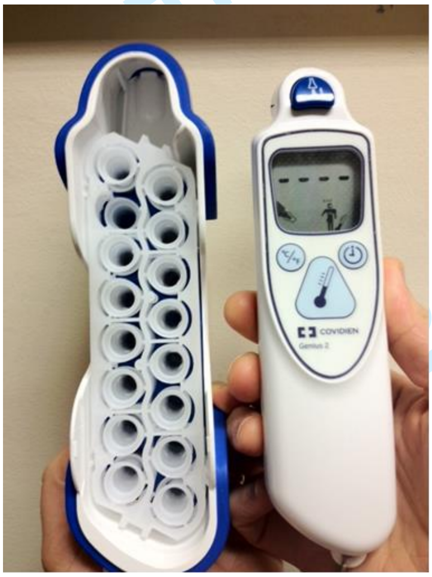

- Push the thermometer tip into a new lens cover from the pack, so that the lens cover clips onto the thermometer.
- Place the tip gently into the ear canal. See photo.

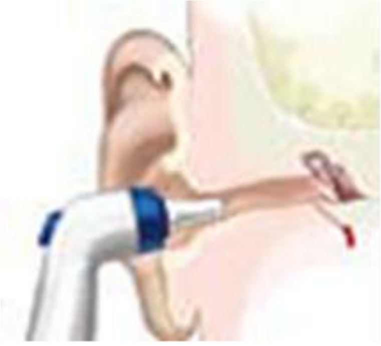

- Press the triangular button on the back of the thermometer.
- Wait for the triple beep before removing from the ear.
- Enter the number into the box on the tablet where indicated.

Adapted from CTRG Template SOP Version 2.1
© Copyright: The University of Oxford 2009

For peer review only - http://bmjopen.bmj.com/site/about/guidelines.xhtml

7. EXTERNAL REFERENCES

http://www.bhsoc.org/frequently-asked-questions/#BPM4

8. CHANGE HISTORY

SOP No 001, version 1

Effective Date: see page 1

Initial Version

Previous version: N/A

SOP no.	Effective Date	Significant Changes	Previous SOP no.

For peer review only

© Copyright: The University of Oxford 2009

STANDARD OPERATING PROCEDURE

Nuffield Department of Obstetrics and Gynaecology

SOP No: 016-V1

SOP Title: Data Entry

SOP Number **016-V1**

SOP Title **Data Entry**

	NAME	TITLE	SIGNATURE	DATE
**Author**	Jude Kemp<br>Fiona Kumar	Data Entry		
**Reviewer**				
**Authoriser**				

**Effective Date:**	
**Review Date:**	

READ BY			
**NAME**	**TITLE**	**SIGNATURE**	**DATE**

For peer review only

Adapted from CTRG Template SOP Version 2.1
© Copyright: The University of Oxford 2009

For peer review only - http://bmjopen.bmj.com/site/about/guidelines.xhtml

STANDARD OPERATING PROCEDURE

Nuffield Department of Obstetrics and Gynaecology

SOP No: 016-V1

SOP Title: Data Entry

1. PURPOSE

The purpose of this SOP is to create clear guidelines on entering data from maternal anaesthetic charts, the partogram and MEOWS charts onto the 4P data base. This will help reduce errors in data entry and ensure high quality data is collected.

2. INTRODUCTION

The 4P study is collecting vital sign (blood pressure, pulse, oxygen saturations, temperature, and respiratory rate) readings from 1000 women throughout pregnancy, labour and the immediate post-partum period. Antenatally and postnatally the data are submitted to the database via a tablet computer.

The data from labour and delivery are manually entered by the research midwives onto the 4P study data base, copying each value form the handwritten charts used for vital sign recording.

When entering data manually from paper records onto an electronic database, there is scope for error by the person transcribing the data.

Some common causes of errors when transcribing data from paper charts onto electronic databases have been identified by Clifton and Watkinson (2015).

These are:

- The chart had illegible handwriting
- The incorrect row in the chart was transcribed
- The incorrect column in the chart was transcribed
- Vital signs were mismatched on the chart
- Typographical errors
- Errors of some other kind

Illegible handwriting was the most common cause of errors, with errors interpreting the grid like structure of the chart being second and third largest.

Whilst it is not possible to eliminate all errors occurring during the transcription of data, this SOP aims to minimise errors by describing in detail the methods used by the research midwives, with information on how to minimise the most commonly occurring errors described above.

Adapted from CTRG Template SOP Version 2.1
© Copyright: The University of Oxford 2009

For peer review only - http://bmjopen.bmj.com/site/about/guidelines.xhtml

STANDARD OPERATING PROCEDURE

Nuffield Department of Obstetrics and Gynaecology

SOP No: 016-V1

SOP Title: Data Entry

3. SCOPE

This SOP applies to all 4P Study midwives and research staff who will be entering data from charts used in labour and delivery onto the 4P database.

4. DEFINITIONS

Anaesthetic chart: A paper chart used by anaesthetists to document all maternal vital signs throughout surgical procedures

Partogram: A paper chart used by midwives to document the progress of labour, including timings of each stage of labour, rupture of amniotic membranes, cervical dilatation, fetal condition and maternal vital signs.

MEOWS (Modified Obstetric Early Warning System) chart: A paper chart used by midwives and doctors to document maternal vital signs during the in-hospital stay. The vital signs are assigned a score, action on these is decided according to this score.

5. RESPONSIBILITIES

It is the responsibility of each research midwife who will be entering data onto the 4P database to familiarise themselves with this SOP and follow the guidance in order to minimise. The SOP must be signed once it has been read.

6. SPECIFIC PROCEDURE

6.1 Entering an Anaesthetic chart:

On the anaesthetic chart, time is represented by boxes and columns. Each box represents five minutes, with each column representing 15 minutes.

Most anaesthetists enter blood pressure and pulse values onto the chart every 5 minutes.

Blood pressure readings are represented by arrow heads, a downward facing one for the systolic value, an upward facing one for the diastolic value.

Heart rate is represented by a dot on the relevant value.

For peer review only

© Copyright: The University of Oxford 2009

For peer review only - http://bmjopen.bmj.com/site/about/guidelines.xhtml

STANDARD OPERATING PROCEDURE

Nuffield Department of Obstetrics and Gynaecology

SOP No: 016-V1

SOP Title: Data Entry

When entering the readings from the chart, the starting time is identified (see photo)

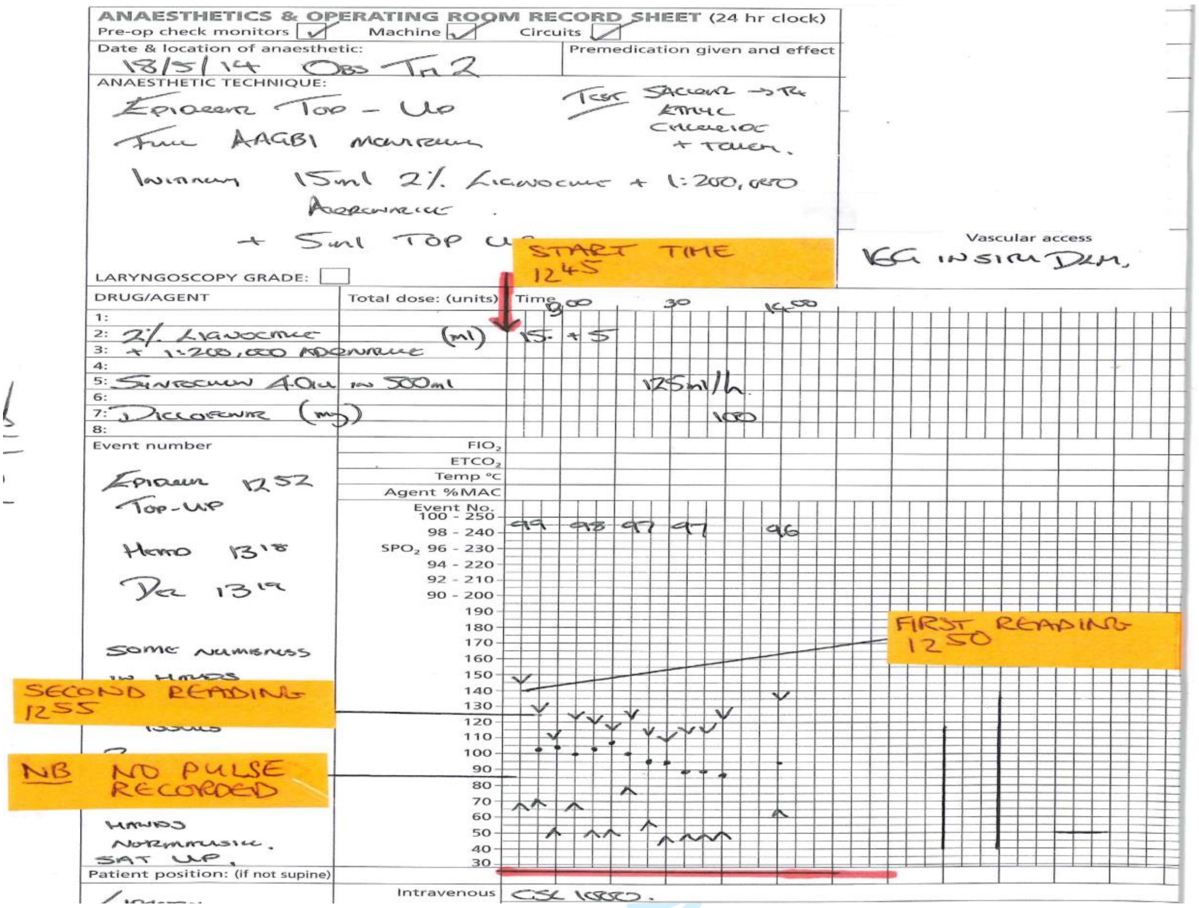

The start time of the chart may not be the time of the first reading to be entered, so it is important to ensure that the value is entered at the correct time, leaving a blank space if necessary.

On the above example, the start time is 12.45, but the first entry by the anaesthetist is 12.50. Readings are then entered every 5 minutes until 13.45, with one last one taken at 14.00.

© Copyright: The University of Oxford 2009

For peer review only - http://bmjopen.bmj.com/site/about/guidelines.xhtml

STANDARD OPERATING PROCEDURE

Nuffield Department of Obstetrics and Gynaecology

SOP No: 016-V1

SOP Title: Data Entry

The chart entry webpage through which the anaesthetic chart is entered (see image below) asks for the date and time to be entered first.

Next it asks for maternal heart rates. These are entered into each box at the corresponding time as seen on the chart above.

Note how the first column is left blank, as there is no reading for 12.45 and the first reading is entered at 12.50.

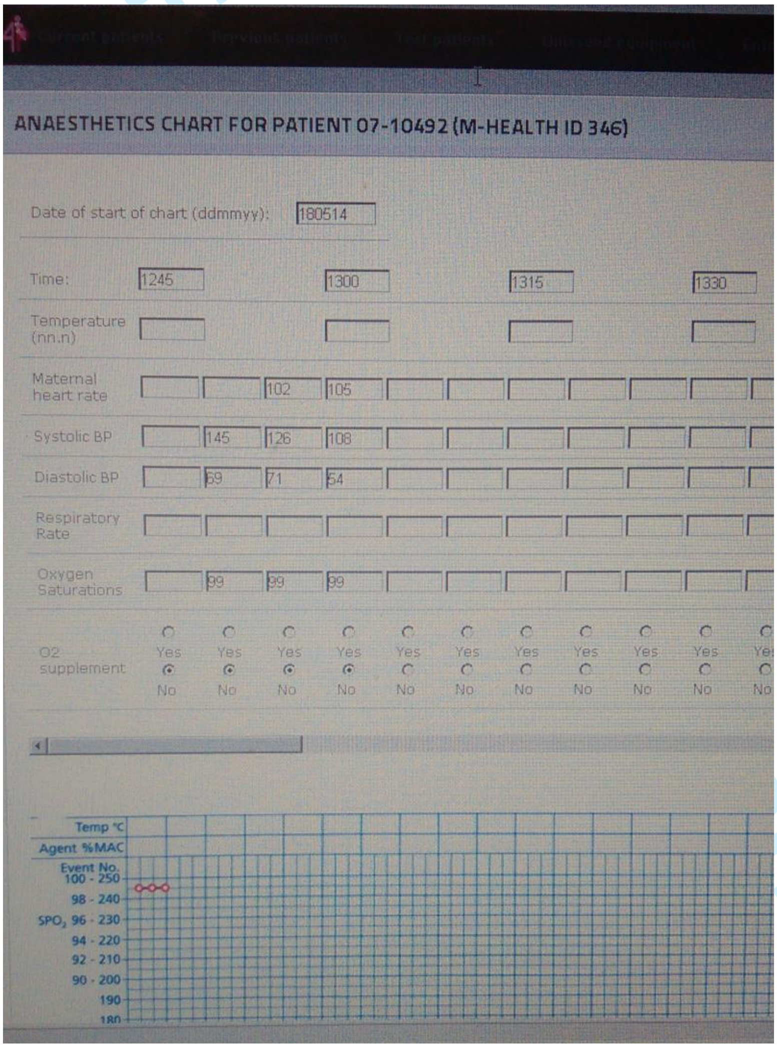

Adapted from CTRG Template SOP Version 2.1
© Copyright: The University of Oxford 2009

For peer review only - http://bmjopen.bmj.com/site/about/guidelines.xhtml

In order to avoid errors, it is easiest to enter all maternal pulses in sequence and then to move on to all systolic blood pressure values, before finishing off with the diastolic blood pressures.

The web page creates a visual representation, which aids with spotting errors as the data are entered (see picture below)

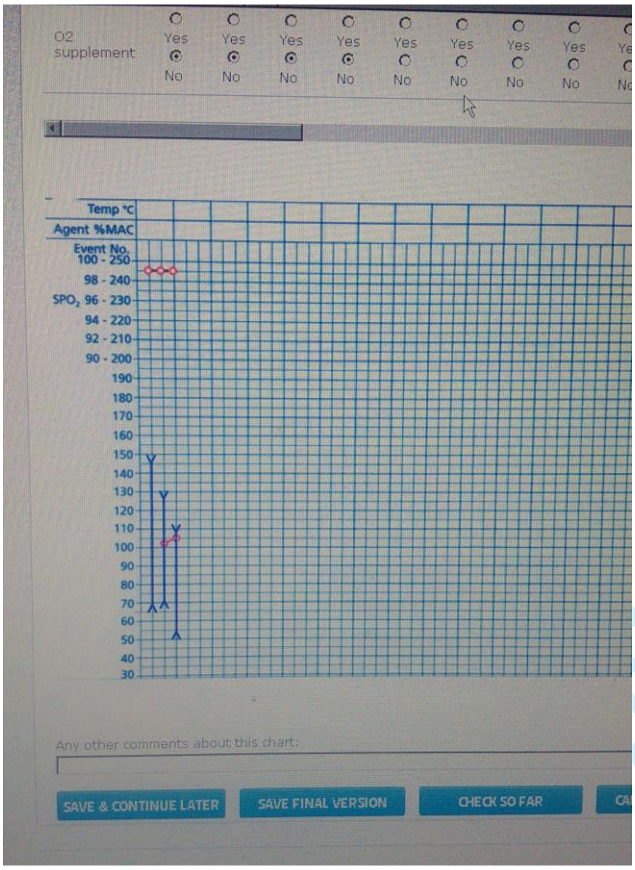

COMMON ERRORS. WRONG COLUMN:

On the above example, there is no pulse recorded at 12.50, so it is important to notice this and not enter the 12.55 pulse into the 12.50 box by mistake.

This section is the main text of the SOP. It details the procedure for the task to be performed.

There should be sufficient detail, clearly expressed, to enable a trained person to perform the procedure without supervision.

There should also be sufficient detail to enable a trained person to use the document to train others to perform the task.

The use of flow diagrams may be useful, especially in complex procedures.

Adapted from CTRG Template SOP Version 2.1
© Copyright: The University of Oxford 2009

For peer review only - http://bmjopen.bmj.com/site/about/guidelines.xhtml

7. FORMS/TEMPLATES TO BE USED

Where Forms/Templates are referenced in the text, the numbers and titles are listed under this section.

8. INTERNAL AND EXTERNAL REFERENCES

This section is used to list all controlled internal references (e.g. SOPs) and external references referred to within the text of the SOP only.

8.1 Internal References

Insert relevant references as required, sufficient for the user to find the source document.

8.2 External References

Insert relevant references as required, sufficient for the user to find the source document. Web references should be included were possible.

9. CHANGE HISTORY

Where the SOP is the initial version:

- SOP No: Record the SOP and version number
- Effective Date: Record effective date of the SOP or “see page 1”
- Significant Changes: State, “Initial version” or “new SOP”
- Previous SOP no.: State “NA”.

Where replacing a previous SOP:

- SOP No: Record the SOP and new version number
- Effective Date: Record effective date of the SOP or “see page 1”
- Significant Changes: Record the main changes from previous SOP
- Previous SOP no.: Record SOP and previous version number

SOP no.	Effective Date	Significant Changes	Previous SOP no.

© Copyright: The University of Oxford 2009

For peer review only - http://bmjopen.bmj.com/site/about/guidelines.xhtml

STANDARD OPERATING PROCEDURE

Nuffield Department of Obstetrics and Gynaecology

SOP No: 017-V1

SOP Title: Home Visit Data Collection by 4P Midwife

SOP Number 017-V1

SOP Title Home Data Collection by 4P Midwife

	NAME	TITLE	SIGNATURE	DATE
**Author**	**Jude Kemp**<br>**Fiona Kumar**	**Research Midwife**		**15 April 2015**
**Reviewer**				
**Authoriser**				

**Effective Date:**	
**Review Date:**	

**READ BY**			
**NAME**	**TITLE**	**SIGNATURE**	**DATE**

For peer review only

Adapted from CTRG Template SOP Version 2.1
© Copyright: The University of Oxford 2009

STANDARD OPERATING PROCEDURE

Nuffield Department of Obstetrics and Gynaecology

SOP No: 017-V1

SOP Title: Home Visit Data Collection by 4P Midwife

1. PURPOSE

The purpose of this SOP is to ensure that the 4P midwives are carrying out home visits to 4P participants in a standardised way. This is to ensure that a full set of high quality data is collected.

2. INTRODUCTION

Research Midwives will be recording medical and obstetric history as well as measuring participant's heart rate, blood pressure, oxygen saturation, respiratory rate and temperature at the booking visit, follow-up visits and one week and two weeks post-delivery.

3. SCOPE

This SOP applies to the 4P research midwives visiting the 4P participants to obtain a full data set.

4. DEFINITIONS

Full data set taken by 4P midwife:

- Medical and obstetric history
- Blood pressure
- Pulse
- Temperature using 4P midwives' 'gold standard' thermometer (Genius 2)
- Pulse oximetry
- Respiration count using accelerometer in smart phone and chest wall movement count

5. RESPONSIBILITIES

It is the responsibility of the 4P midwives to become familiar with the content of this SOP as they will be recording data during home visits.

Adapted from CTRG Template SOP Version 2.1
© Copyright: The University of Oxford 2009

For peer review only - http://bmjopen.bmj.com/site/about/guidelines.xhtml

6. SPECIFIC PROCEDURE

- Access the 4P website via 3/4G or wifi and open the electronic crf that corresponds with the participant's ID to complete the relevant medical and obstetric history sections
- Exit the application and open the android app for 4P data input and enter the participant's ID
- Connect the participant's finger to the pulse oximeter.
- Press the 'connect to finger probe' button on the app and ensure that the Nonin pulse oximeter is connected to the tablet.
- Explain to participant that her heart rate will be taken by palpating the radial pulse for 60 seconds.
- Palpate participant's radial pulse for 60 seconds and enter the measurement into the appropriate box on the app.
- Explain that the respiratory rate will be obtained using a smart phone placed on the participant's chest.
- Open the breathing rate app on the smart phone, enter the participant ID, attach the smart phone around the participant's neck and ask her to sit back in a chair so that the phone is resting on her chest.
- Ask participant to refrain from speaking during the measurement.
- Press the 'start counting breaths' button on the smartphone. A bleep will be heard.
- Wait for a second bleep. The word 'count' will appear on the screen. Press the 'count respirations' button on the tablet.
- For every breath, at the peak of inspiration, press the on-screen button on the tablet. The count takes 60 seconds.
- Count the participant's chest wall movements until a third bleep is heard from the smartphone and the word 'recording' appears on the screen.
- Press 'stop recording' on the screen and remove the smart phone from the participant's neck.
- Take the participant's temperature using the 4P Genius thermometer and enter it onto the tablet
- Take the participant's blood pressure;

For peer review only

© Copyright: The University of Oxford 2009

For peer review only - http://bmjopen.bmj.com/site/about/guidelines.xhtml

The appropriate cuff size should be used:

Standard cuff bladder 12-26 cm for the majority of adult arms (arm circumference 33-50 cm)

Large cuff bladder 12-40 cm for obese arms (arm circumference > 50 cm)

Small cuff bladder 12-18 cm for lean adult arms (arm circumference 23-33 cm)

(Combined European Society/Nice/British Hypertension Society recommendations, 2012) http://www.bhsoc.org/frequently-asked-questions/#BPM4

Ask the participant to

- Sit with her arm stretched out and supported, for example on the arm of a chair or a cushion.
- The feet should be flat on the floor.
- Sit quietly and relax.
- Wear clothing with a loose sleeve as she will need to push it up. If the sleeve is tight, ask the participant to slip her arm out of the sleeve.
- Do not push tight sleeves up, as she will not be able to position the cuff correctly.
- Slip her arm into the cuff with the black tubing at the front and move it up the arm, 3cm above the elbow.
- Fasten the Velcro and adjust the position of the cuff if needed.
- Press the 'on' button. Explain that the cuff will go tight, which may be slightly uncomfortable.
- Encourage the participant not to talk during the measurement.
- Enter the BP value on the tablet computer.

IMPORTANT

Explain that a high BP reading will cause a message to appear onscreen, asking her to repeat the measurement.

If the measurement is still high, a message will appear, asking her to access medical help Explain to the participant that it is her responsibility to access medical assistance and that the data she is recording are NOT monitored by a doctor or midwife.

© Copyright: The University of Oxford 2009

For peer review only - http://bmjopen.bmj.com/site/about/guidelines.xhtml

- Enter the respiration count onto the tablet.
- Explain to the participant that the measurements will be taken a second time and carry them out as above, beginning with the palpation of the radial pulse.

7. CHANGE HISTORY

Where the SOP is the initial version:

- SOP No: 0017
- Effective Date: see page 1
- Significant Changes: Initial version
- Previous SOP no.: NA

SOP no.	Effective Date	Significant Changes	Previous SOP no.

Adapted from CTRG Template SOP Version 2.1
© Copyright: The University of Oxford 2009

For peer review only - http://bmjopen.bmj.com/site/about/guidelines.xhtml
